# A neuropsychosocial signature predicts longitudinal symptom changes in women with irritable bowel syndrome

**DOI:** 10.1038/s41380-021-01375-9

**Published:** 2021-11-24

**Authors:** Ravi R. Bhatt, Arpana Gupta, Jennifer S. Labus, Cathy Liu, Priten P. Vora, Jean Stains, Bruce D. Naliboff, Emeran A. Mayer

**Affiliations:** 1 Gail and Gerald Oppenheimer Family Center for Neurobiology of Stress and Resilience, Vatche and Tamar Manoukian Division of Digestive Diseases, David Geffen School of Medicine at UCLA, Los Angeles, USA; 2 Imaging Genetics Center, Mark and Mary Stevens Neuroimaging and Informatics Institute, Keck School of Medicine at USC, University of Southern California, Los Angeles, USA

**Keywords:** Predictive markers, Neuroscience

## Abstract

Irritable bowel syndrome (IBS) is a common disorder of brain-gut interactions characterized by chronic abdominal pain, altered bowel movements, often accompanied by somatic and psychiatric comorbidities. We aimed to test the hypothesis that a baseline phenotype composed of multi-modal neuroimaging and clinical features predicts clinical improvement on the IBS Symptom Severity Scale (IBS-SSS) at 3 and 12 months without any targeted intervention. Female participants (*N* = 60) were identified as “improvers” (50-point decrease on IBS-SSS from baseline) or “non-improvers.” Data integration analysis using latent components (DIABLO) was applied to a training and test dataset to determine whether a limited number of sets of multiple correlated baseline’omics data types, including brain morphometry, anatomical connectivity, resting-state functional connectivity, and clinical features could accurately predict improver status. The derived predictive models predicted improvement status at 3-months and 12-months with 91% and 83% accuracy, respectively. Across both time points, non-improvers were classified as having greater correlated morphometry, anatomical connectivity and resting-state functional connectivity characteristics within salience and sensorimotor networks associated with greater pain unpleasantness, but lower default mode network integrity and connectivity. This suggests that non-improvers have a greater engagement of attentional systems to perseverate on painful visceral stimuli, predicting IBS exacerbation. The ability of baseline multimodal brain-clinical signatures to predict symptom trajectories may have implications in guiding integrative treatment in the age of precision medicine, such as treatments targeted at changing attentional systems such as mindfulness or cognitive behavioral therapy.

## Introduction

Irritable bowel syndrome (IBS) is characterized by altered brain-gut interactions, chronic abdominal pain, altered bowel movements, and on occasion, psychiatric symptoms, (e.g., anxiety) [1]. In 2012, an estimated 11.3% of the global population was diagnosed with IBS, 65% being female [2, 3]. For a subset of patients, IBS results in debilitating symptoms and greatly impacts quality of life [4]. Effective management of IBS requires an integrative approach which may include a combination of educational, pharmacological, psychological, and behavioral treatments [5]. Identifying biomarkers to predict symptom improvement can assist specialists in the practice of precision medicine. Tailored and multifaceted treatments are critical when symptoms vary from patient to patient, such as with IBS and chronic pain [1, 6**–**8].

IBS pathophysiology has been informed by neuroimaging studies that reveal affected innate and elicited brain structure and activity. Brain networks involved in central processing and modulation of IBS-related visceral pain include the salience, sensorimotor, default mode, emotional arousal, central executive, and central autonomic networks [9, 10]. Evidence shows dynamic interactions between brain networks are altered in IBS. These adaptations may influence key information processing systems in terms of attention, memory, perceptions, problem solving, and planning, as well as, autonomic hyperarousal [10]. Efferent signals from the periaqueductal gray and raphe nuclei play crucial roles in descending endogenous pain modulation via limbic and cortical input, regulating dorsal horn excitability in response to visceral afferent input [11]. Vagal and sympathetic efferent projections from the central autonomic network through the brainstem modulate the activity of the enteric nervous system (ENS), and afferents from the ENS send viscerosensory signals back to the brain. A variety of mechanisms contributing to IBS symptoms have been identified which are involved in brain-gut microbiome interactions, including the gut microbiome, metabolome, neuroimmune interactions, genetics, as well as psychological and behavioral history [1, 10, 12].

Moreover, many psychosocial and environmental factors are known to play a large role in the susceptibility, development, symptom flares, and chronicity of IBS [1, 10]. These include, but are not limited to, environmental aspects such as early life adversity [13**–**15], personality traits such as neuroticism [16, 17], coping strategies such as catastrophizing behavior [18, 19], and negative emotions and psychiatric disorders such as anxiety, depression, and somatoform disorders [1, 20, 21]. Throughout life, all of these factors can influence dysregulation of the brain–gut axis through neural, neuroimmune, and neuroendocrine pathways which connect the brain and the gastrointestinal tract [1, 10]. Considering psychosocial factors when assessing IBS pathophysiology in addition to the biological factors underpinning IBS leads to more targeted and efficacious pharmacologic and non-pharmacologic treatments [1, 10, 12].

Based on the possibility of using neuroimaging and behavioral data to estimate disease trajectory in brain–gut interactions [12], identification of a signature from longitudinal data could help identify those with a more negative disease trajectory and responsiveness to treatment. In other chronic pain syndromes such as chronic pelvic pain [22] and low back pain [23**–**25], baseline neuroimaging data have been shown to predict symptom changes longitudinally, but to date no multi-modal brain and clinical phenotype has been identified which can predict symptom trajectories in IBS.

This observational cohort study reports the results of an ongoing deep phenotyping study (NCT02693730) in patients with IBS. We aimed to predict a baseline phenotype using patient data composed of multi-modal neuroimaging, behavioral testing, and clinical self-report questionnaires and hypothesized that an integrated neuropsychosocial signature is able to classify IBS patients into those that improve over time and those who do not. To test this hypothesis, we examined changes across these variables at 3 and 12 months by IBS symptom severity. We analyzed three whole-brain imaging modalities: structural morphometry, resting-state functional connectivity, and anatomical connectivity assessed by diffusion imaging and used a multi-omics integrative approach to identify distinct signatures [26]. Additionally, behavioral data and clinical questionnaires were used as an additional “-omic” type to understand how relationships between the brain and behavior could predict longitudinal outcomes. This signature makes it possible to connect specific psychosocial factors (including early life adversity, resilience, symptom severity and perceived pain), with whole-brain neurobiological patterns of morphometry, resting-state functional connectivity, and anatomical connectivity.

## Methods

### Participants

Sixty female participants were recruited by the Center for Neurobiology of Stress and Resilience at UCLA starting in 2016 (mean age = 29.08, SD = 11.46). All met Rome III criteria for IBS, including subtypes of bowel habit predominance [27]. Those reporting histories of the following were excluded: other gastrointestinal illnesses; eating disorders; rectal prolapse; severe hemorrhoids; gastric, abdominal, or colon surgery; recent steroid use; insulin-dependent diabetes; kidney disease; heart disease; hypertension; cancer; significant lung disease and/or neurological condition (TBI, seizures); major surgery within 6 months of study onset, other chronic illness and/or pain condition; nicotine use; alcohol or drug misuse, psychiatric or developmental disorders impairing self-report, use of HIV and/or SSRI medications, recent clinical trial participation (i.e., 28 days), and variables obstructing MRI testing (e.g., metal implants, whole-limb tattoos, claustrophobia, untreated anxiety, or panic attacks). All participants received informed consent about the procedures of the study.

### Study design

In this natural observation study, patients with IBS participated in multimodal brain imaging, quantitative sensory testing, and a broad range of psychosocial questionnaires at baseline, 3 and 12 months of follow-up. There were 122 patients screened for eligibility; 78 confirmed eligible; 76 decided to enroll in the study. The sample size dropped to 60 at 3 months, and to 43 at 12 months due to attrition. There were 21 subjects who dropped from the study across 12 months, resulting in 55 distinct subjects completing the study. As this analysis is part of a larger, ongoing deep phenotyping study, the full procedures are in the Supplementary Methods: Study Design. Briefly, the initial screening visit consisted of the following in order; informed consent, assessing eligibility, reviewing contaminant medications, taking vital signs, a medical history and exam, a psychological interview, a blood sample, thermal quantitative sensory testing (QST), a battery of psychosocial questionnaires, and finally a urine pregnancy test. The following visit (visit 2) was the first MRI visit and consisted of the following in order; contaminant medication conditions were reviewed, adverse events reviewed, a battery of psychosocial questionnaires, brain imaging procedures, a urine pregnancy test, and a stool sample. The following MRI visits at 3 and 12 months included the following in order; eligibility assessment, contaminant conditions review, weight and vital signs, blood sample, adverse events review, thermal QST, psychosocial questionnaires, brain imaging, a urine pregnancy test, and a stool sample.

### Behavioral data/Clinical questionnaires

Behavioral and clinical variables taken at baseline are detailed in the “Supplementary Methods: Questionnaires”. The IBS Symptom Severity Scale (IBS-SSS) measured patients’ self-report of IBS symptom severity. Scores less than 75 indicate controls, between 75 and 175 indicate mild IBS, between 175 and 300 indicate moderate IBS, and greater than 300 indicates severe IBS. It has good reproducibility and is sensitive to change [28]. Over 12 months at 3-month intervals, each participant completed the IBS-SSS. IBS-SSS scores were used to separate participants into two distinct symptom groups. Patient symptoms were defined as “improving” if IBS-SSS scores were reduced by 50-points or more. For ease of discussion, this symptom group was labeled, “Improvers.” Patient symptoms were otherwise defined as persistent or worsening (i.e., no change, increases, or decreases of less than 50-points in IBS-SSS score), and placed into the “Non-Improvers” group [28]. As hormonal status can also play a role in abdominal symptoms, psychological state, and pain perception [29], the use of hormonal contraceptives was also recorded (*N* = 15, “Supplementary Methods: Hormonal Status and use of Contraceptives” Table S1). Fifty-five women were premenopausal, one woman was perimenopausal, and four women were postmenopausal.

Other self-report variables used in our analyses included the *Bowel Symptom Questionnaire* (BSQ) [30], Hospital Anxiety and Depression Scale (HADS) [31], State-Trait Anxiety Inventory (STAI) [32], Perceived Stress Scale (PSS) [33], International Personality Item Pool (IPIP) [34], Early Trauma Inventory (ETI) [35], Complex Multi-Symptom Inventory (CMSI) [36], and the Connor-Davidson Resilience Scale (CD-RISC) [37].

### Behavioral data

Quantitative sensory testing (QST) at baseline involved a brief thermal pain sensitivity test using an electrically heated circular thermode placed on the forearm. Thermode surface area was 3 cm × 3 cm. Testing consisted of six randomized trials. The thermode temperature increased from 32 °C by 0.5 °C/s with each trial, and returned to 32 °C by 10 °C/s. In three trials, participants spoke when pain was perceived. This was recorded as “pain threshold”. In the other three trials, participants spoke when pain was intolerable. This was recorded as “pain tolerance”. A delay of 20 s occurred between trials. The thermode placement was slightly altered between each trial to avoid the sensitization or habituation of cutaneous receptors [38]. Pain threshold and tolerance scores were averaged to compute final scores. Pain intensity and unpleasantness of the tasks were then rated on a 0–20 visual analog scale using the Gracely Pain Scale [39].

### Neuroimaging acquisition and overview of multimodal image processing

See Supplementary Material: Neuroimaging Acquisition and Structural/Functional/Diffusion Image Processing for neuroimaging acquisition parameters and processing methods for each neuroimaging modality. A whole-brain region of interest (ROI) approach was used to create the datasets used in the DIABLO analysis. For cortical regions, the Human Connectome Project atlas [40] (HCP-MMP), which has shown to have greater amounts of reproducibility, parcellation reliability, agreement with task activation, and greatest overlap over Broadman areas compared to other major brain atlases [41], was used. For subcortical regions, the Harvard-Oxford subcortical atlas was used [42**–**45]. Based on these ROIs (Table 1), measures of brain morphometry, resting-state functional connectivity, and anatomical connectivity were derived. Participants’ neuroimaging data were treated as separate datasets for each modality, and each dataset went through separate preprocessing methods prior to DIABLO. See “Supplementary Material: Neuroimaging data preparation for DIABLO”*.*

**Table 1:** Regions of interest.

Parcel index	Area name	Area description	HCP network	Destrieux/lHarvard-Oxford full name	Destrieux/Harvard-Oxford short name
1	V1	Primary visual cortex	Primary visual cortex (V1)	Pole_occipital	OcPo
2	MST	Medial superior temporal area	MT+ Complex and neighboring areas	S_occipital_ant	AOcS
3	V6	Sixth visual area	Dorsal stream visual cortex	G_occipital_sup	SupOcG
				G_cuneus	Cun
4	V2	Second visual area	Early visual cortex	Pole_occipital	OcPo
5	V3	Third visual area	Early visual cortex	G_occipital_middle	MOcG
				G_occipital_sup	SupOcG
				Pole_occipital	OcPo
				G_and_S_occipital_inf	InfOcG_S
				S_oc_middle_and_Lunatus	MOcS_LuS
6	V4	Fourth visual area	Early visual cortex	S_oc_middle_and_Lunatus	MOcS_LuS
				G_occipital_middle	MOcG
7	V8	Eighth visual area	Ventral stream visual cortex	G_oc_temp_lat_fusifor	FuG
				G_and_S_occipital_inf	InfOcG_S
8	4	Primary motor cortex	Somatosensory and motor cortex	G_precentral	PRCG
				S_central	CS
				G_and_S_subcentral	SbCG_S
9	3b	Primary sensory cortex	Somatosensory and motor cortex	S_central	CS
				G_and_S_paracentral	PaCL_S
				G_postcentral	PosCG
10	FEF	Frontal eye fields	Premotor cortex	S_precentral_sup_part	SupPrCs
				G_precentral	PRCG
				G_front_middle	MFG
11	PEF	Premotor eye field	Premotor cortex	S_precentral_inf_part	InfPrCS
				G_precentral	PRCG
12	55b	Area 55b	Premotor cortex	S_precentral_inf_part	InfPrCS
				G_precentral	PRCG
13	V3A	Area V3A	Dorsal stream visual cortex	G_occipital_sup	SupOcG
				S_oc_sup_and_transversal	SupOcS_TrOcS
14	RSC	RetroSplenial complex	Posterior cingulate cortex	G_cingul_Post_dorsal	PosDCgG
				G_cingul_Post_ventral	PosVCgG
				S_pericallosal	PerCaS
15	POS2	Parieto-occipital Sulcus area 2	Posterior cingulate cortex	S_parieto_occipital	POcS
				G_precuneus	PrCun
16	V7	Seventh visual area	Dorsal stream visual cortex	G_occipital_sup	SupOcG
				S_oc_sup_and_transversal	SupOcS_TrOcS
17	IPS1	IntraParietal sulcus area 1	Dorsal stream visual cortex	S_oc_sup_and_transversal	SupOcS_TrOcS
				S_intrapariet_and_P_trans	IntPS_TrPS
				G_occipital_sup	SupOcG
18	FFC	Fusiform face complex	Ventral stream visual cortex	G_oc_temp_lat_fusifor	FuG
				G_and_S_occipital_inf	InfOcG_S
				S_oc_temp_lat	LOcTS
19	V3B	Area V3B	Dorsal stream visual cortex	S_oc_sup_and_transversal	SupOcS_TrOcS
20	LO1	Area lateral occipital 1	MT+ complex and neighboring areas	S_oc_middle_and_Lunatus	MOcS_LuS
				G_occipital_middle	MOcG
21	LO2	Area lateral occipital 2	MT+ complex and neighboring areas	G_occipital_middle	MOcG
				G_and_S_occipital_inf	InfOcG_S
22	PIT	Posterior inferotemporal complex	Ventral stream visual cortex	G_and_S_occipital_inf	InfOcG_S
23	MT	Middle temporal area	MT+ complex and neighboring areas	S_occipital_ant	AOcS
				G_occipital_middle	MOcG
24	A1	Primary auditory cortex	Early auditory cortex	G_temp_sup_G_T_transv	HG
				S_temporal_transverse	TrTs
25	PSL	PeriSylvian language area	Temporo-parieto-occipital junction	G_pariet_inf_Supramar	SuMarG
				G_temp_sup_Plan_tempo	TPl
26	SFL	Superior frontal language area	Dorsolateral prefronal cortex	G_front_sup	SupFG
27	PCV	PreCuneus visual area	Posterior cingulate cortex	G_precuneus	PrCun
				S_subparietal	SbPS
28	STV	Superior temporal visual area	Temporo-parieto-occipital junction	G_pariet_inf_Supramar	SuMarG
				S_temporal_sup	SupTS
				G_temp_sup_Lateral	SupTGLp
29	7Pm	Medial area 7P	Superior parietal cortex	G_precuneus	PrCun
30	7m	Area 7m	Posterior cingulate cortex	G_precuneus	PrCun
				S_subparietal	SbPS
31	POS1	Parieto-occipital sulcus area 1	Posterior cingulate cortex	G_precuneus	PrCun
				S_parieto_occipital	POcS
				S_calcarine	CcS
				G_cingul_Post_ventral	PosVCgG
32	23d	Area 23d	Posterior cingulate cortex	G_cingul_Post_dorsal	PosDCgG
				G_and_S_cingul_Mid_Post	MPosCgG_S
33	v23ab	Area ventral 23 a+b	Posterior cingulate cortex	G_cingul_Post_dorsal	PosDCgG
				G_cingul_Post_ventral	PosVCgG
				G_precuneus	PrCun
				S_subparietal	SbPS
34	d23ab	Area dorsal 23 a+b	Posterior cingulate cortex	G_cingul_Post_dorsal	PosDCgG
35	31pv	Area 31p ventral	Posterior cingulate cortex	S_subparietal	SbPS
36	5m	Area 5m	Paracentral lobular and mid-cingulate cortex	G_and_S_paracentral	PaCL_S
37	5mv	Area 5m ventral	Paracentral lobular and mid-cingulate cortex	S_cingul_Marginalis	CgSMarp
38	23c	Area 23c	Posterior cingulate cortex	G_and_S_cingul_Mid_Post	MPosCgG_S
				S_cingul_Marginalis	CgSMarp
39	5L	Area 5L	Paracentral lobular and mid-cingulate cortex	G_precuneus	PrCun
				G_and_S_paracentral	PaCL_S
				G_parietal_sup	SupPL
40	24dd	Dorsal area 24d	Paracentral lobular and mid-cingulate cortex	G_front_sup	SupFG
				S_cingul_Marginalis	CgSMarp
				G_and_S_cingul_Mid_Post	MPosCgG_S
				G_and_S_paracentral	PaCL_S
41	24dv	Ventral area 24d	Paracentral lobular and mid-cingulate cortex	G_and_S_cingul_Mid_Post	MPosCgG_S
42	7AL	Lateral area 7A	Superior parietal cortex	G_parietal_sup	SupPL
43	SCEF	Supplementary and cingulate eye field	Paracentral lobular and mid-cingulate cortex	G_front_sup	SupFG
				G_and_S_cingul_Mid_Ant	MACgG_S
44	6ma	Area 6m anterior	Paracentral lobular and mid-cingulate cortex	S_front_sup	SupFS
				G_front_sup	SupFG
45	7Am	Medial area 7A	Superior parietal cortex	G_parietal_sup	SupPL
				G_precuneus	PrCun
46	7Pl	Lateral area 7P	Superior parietal cortex	G_parietal_sup	SupPL
47	7PC	Area 7PC	Superior parietal cortex	G_parietal_sup	SupPL
				S_intrapariet_and_P_trans	IntPS_TrPS
48	LIPv	Area lateral intraparietal ventral	Superior parietal cortex	S_intrapariet_and_P_trans	IntPS_TrPS
				G_parietal_sup	SupPL
49	VIP	Ventral intraparietal complex	Superior parietal cortex	G_parietal_sup	SupPL
				S_intrapariet_and_P_trans	IntPS_TrPS
50	MIP	Medial intraparietal area	Superior parietal cortex	G_parietal_sup	SupPL
				S_intrapariet_and_P_trans	IntPS_TrPS
51	1	Area 1	Somatosensory and motor cortex	G_postcentral	PosCG
				G_and_S_subcentral	SbCG_S
52	2	Area 2	Somatosensory and motor cortex	S_postcentral	PosCS
				G_postcentral	PosCG
53	3a	Area 3a	Somatosensory and motor cortex	S_central	CS
				G_and_S_subcentral	SbCG_S
54	6d	Dorsal area 6	Premotor cortex	G_precentral	PRCG
55	6mp	Area 6mp	Paracentral lobular and mid-cingulate cortex	S_precentral_sup_part	SupPrCs
56	6v	Ventral area 6	Premotor cortex	G_precentral	PRCG
57	p24pr	Area posterior 24 prime	Anterior mid-cingulate and medial prefrontal cortex	G_and_S_cingul_Mid_Post	MPosCgG_S
58	33pr	Area 33 prime	Anterior mid-cingulate and medial prefrontal cortex	G_and_S_cingul_Mid_Ant	MACgG_S
				S_pericallosal	PerCaS
59	a24pr	Anterior 24 prime	Anterior mid-cingulate and medial prefrontal cortex	G_and_S_cingul_Mid_Ant	MACgG_S
60	p32pr	Area p32 prime	Anterior mid-cingulate and medial prefrontal cortex	G_and_S_cingul_Mid_Ant	MACgG_S
61	a24	Area a24	Anterior mid-cingulate and medial prefrontal cortex	G_and_S_cingul_Ant	ACgG_S
62	d32	Area dorsal 32	Anterior mid-cingulate and medial prefrontal cortex	G_and_S_cingul_Ant	ACgG_S
63	8BM	Area 8BM	Anterior mid-cingulate and medial prefrontal cortex	G_front_sup	SupFG
64	p32	Area p32	Anterior mid-cingulate and medial prefrontal cortex	G_and_S_cingul_Ant	ACgG_S
65	10r	Area 10r	Anterior mid-cingulate and medial prefrontal cortex	G_and_S_cingul_Ant	ACgG_S
				S_suborbital	SbOrS
66	47m	Area 47m	Orbital and polar frontal cortex	S_orbital_H_Shaped	OrS
				G_orbital	OrG
67	8Av	Area 8Av	Dorsolateral prefronal cortex	G_front_middle	MFG
68	8Ad	Area 8Ad	Dorsolateral prefronal cortex	S_front_sup	SupFS
69	9m	Area 9 middle	Anterior mid-cingulate and medial prefrontal cortex	G_front_sup	SupFG
				G_and_S_cingul_Ant	ACgG_S
70	8BL	Area 8B lateral	Dorsolateral prefronal cortex	G_front_sup	SupFG
71	9p	Area 9 posterior	Dorsolateral prefronal cortex	G_front_sup	SupFG
				S_front_sup	SupFS
72	10d	Area 10d	Orbital and polar frontal cortex	G_and_S_transv_frontopol	TrFPoG_S
				G_front_sup	SupFG
73	8C	Area 8C	Dorsolateral prefronal cortex	G_front_middle	MFG
				S_front_inf	InfFS
				S_precentral_inf_part	InfPrCS
74	44	Area 44	Inferior frontal cortex	G_front_inf_Opercular	InfFGOpp
				G_front_inf_Triangul	InfFGTrip
				Lat_Fis_ant_Vertical	ALSVerp
75	45	Area 45	Inferior frontal cortex	G_front_inf_Triangul	InfFGTrip
				Lat_Fis_ant_Horizont	ALSHorp
76	47l	Area 47l (47 lateral)	Inferior frontal cortex	G_orbital	OrG
				G_front_inf_Orbital	InfFGOrp
				S_orbital_H_Shaped	OrS
77	a47r	Area anterior 47r	Orbital and polar frontal cortex	S_orbital_H_Shaped	OrS
				G_orbital	OrG
				G_front_middle	MFG
				G_and_S_frontomargin	FMarG_S
78	6r	Rostral area 6	Premotor cortex	S_precentral_inf_part	InfPrCS
				G_precentral	PRCG
				G_front_inf_Opercular	InfFGOpp
79	IFJa	Area IFJa	Inferior frontal cortex	S_front_inf	InfFS
				G_front_inf_Opercular	InfFGOpp
80	IFJp	Area IFJp	Inferior frontal cortex	S_front_inf	InfFS
				S_precentral_inf_part	InfPrCS
81	IFSp	Area IFSp	Inferior frontal cortex	S_front_inf	InfFS
				G_front_inf_Triangul	InfFGTrip
82	IFSa	Area IFSa	Inferior frontal cortex	S_front_inf	InfFS
				G_front_inf_Triangul	InfFGTrip
				S_orbital_lateral	LORs
83	p9-46v	Area posterior 9-46v	Dorsolateral prefronal cortex	G_front_middle	MFG
				S_front_inf	InfFS
84	46	Area 46	Dorsolateral prefronal cortex	S_front_inf	InfFS
				G_front_middle	MFG
				S_front_sup	SupFS
				S_front_middle	MFS
85	a9-46v	Area anterior 9-46v	Dorsolateral prefronal cortex	G_front_middle	MFG
				S_front_inf	InfFS
86	9-46d	Area 9-46d	Dorsolateral prefronal cortex	S_front_middle	MFS
				S_front_sup	SupFS
				G_front_middle	MFG
87	9a	Area 9 anterior	Dorsolateral prefronal cortex	G_front_middle	MFG
				G_front_sup	SupFG
				S_front_sup	SupFS
				G_and_S_transv_frontopol	TrFPoG_S
88	10v	Area 10v	Anterior mid-cingulate and medial prefrontal cortex	G_rectus	RG
				G_and_S_frontomargin	FMarG_S
89	a10p	Area anterior 10p	Orbital and polar frontal cortex	G_and_S_frontomargin	FMarG_S
				G_and_S_transv_frontopol	TrFPoG_S
90	10pp	Polar 10p	Orbital and polar frontal cortex	G_and_S_transv_frontopol	TrFPoG_S
				G_and_S_frontomargin	FMarG_S
91	11l	Area 11l	Orbital and polar frontal cortex	G_orbital	OrG
				S_orbital_H_Shaped	OrS
92	13l	Area 13l	Orbital and polar frontal cortex	S_orbital_H_Shaped	OrS
				G_orbital	OrG
93	OFC	Orbital frontal complex	Orbital and polar frontal cortex	G_orbital	OrG
				S_orbital_med_olfact	MedOrS
				G_rectus	RG
94	47s	Area 47s	Orbital and polar frontal cortex	G_orbital	OrG
				S_circular_insula_ant	ACirIns
95	LIPd	Area lateral intraparietal dorsal	Superior parietal cortex	S_intrapariet_and_P_trans	IntPS_TrPS
96	6a	Area 6 anterior	Premotor cortex	S_front_sup	SupFS
				S_precentral_sup_part	SupPrCs
				G_front_middle	MFG
				G_precentral	PRCG
97	i6-8	Inferior 6-8 transitional area	Dorsolateral prefronal cortex	G_front_middle	MFG
				S_front_sup	SupFS
98	s6-8	Superior 6-8 transitional area	Dorsolateral prefronal cortex	G_front_sup	SupFG
				S_front_sup	SupFS
99	43	Area 43	Posterior opercular cortex	G_and_S_subcentral	SbCG_S
				G_front_inf_Opercular	InfFGOpp
100	OP4	Area OP4/PV	Posterior opercular cortex	G_and_S_subcentral	SbCG_S
				G_pariet_inf_Supramar	SuMarG
101	OP1	Area OP1/SII	Posterior opercular cortex	G_pariet_inf_Supramar	SuMarG
				G_and_S_subcentral	SbCG_S
				Lat_Fis_post	PosLS
102	OP2-3	Area OP2-3/VS	Posterior opercular cortex	Lat_Fis_post	PosLS
				G_and_S_subcentral	SbCG_S
				S_circular_insula_sup	SupCirInS
103	52	Area 52	Insular and frontal opercular cortex	S_circular_insula_inf	InfCirIns
				Lat_Fis_post	PosLS
104	RI	RetroInsular cortex	Early auditory cortex	Lat_Fis_post	PosLS
				G_temp_sup_Plan_tempo	TPl
105	PFcm	Area PFcm	Posterior opercular cortex	G_pariet_inf_Supramar	SuMarG
				Lat_Fis_post	PosLS
106	PoI2	Posterior insular area 2	Insular and frontal opercular cortex	G_Ins_lg_and_S_cent_ins	LoInG_CInS
				S_circular_insula_inf	InfCirIns
107	TA2	Area TA2	Auditory association cortex	G_temp_sup_Plan_polar	PoPl
				S_circular_insula_inf	InfCirIns
108	FOP4	Frontal OPercular area 4	Insular and frontal opercular cortex	G_front_inf_Opercular	InfFGOpp
				S_circular_insula_sup	SupCirInS
109	MI	Middle insular area	Insular and frontal opercular cortex	S_circular_insula_sup	SupCirInS
				G_insular_short	ShoInG
110	Pir	Pirform cortex	Insular and frontal opercular cortex	G_Ins_lg_and_S_cent_ins	LoInG_CInS
				S_circular_insula_inf	InfCirIns
				G_temp_sup_Plan_polar	PoPl
111	AVI	Anterior ventral insular area	Insular and frontal opercular cortex	G_insular_short	ShoInG
				S_circular_insula_ant	ACirIns
				G_orbital	OrG
112	AAIC	Anterior agranular insula complex	Insular and frontal opercular cortex	S_circular_insula_ant	ACirIns
				G_insular_short	ShoInG
				G_orbital	OrG
113	FOP1	Frontal OPercular area 1	Posterior opercular cortex	G_front_inf_Opercular	InfFGOpp
				S_circular_insula_sup	SupCirInS
114	FOP3	Frontal OPercular area 3	Insular and frontal opercular cortex	S_circular_insula_sup	SupCirInS
115	FOP2	Frontal OPercular area 2	Insular and frontal opercular cortex	S_circular_insula_sup	SupCirInS
				G_front_inf_Opercular	InfFGOpp
116	PFt	Area PFt	Inferior parietal cortex	G_pariet_inf_Supramar	SuMarG
				S_postcentral	PosCS
117	AIP	Anterior intraparietal area	Superior parietal cortex	S_intrapariet_and_P_trans	IntPS_TrPS
				S_postcentral	PosCS
				G_parietal_sup	SupPL
118	EC	Entorhinal cortex	Medial temporal cortex	G_oc_temp_med_Parahip	PaHipG
119	PreS	PreSubiculum	Medial temporal cortex	G_oc_temp_med_Parahip	PaHipG
120	H	Hippocampus	Medial temporal cortex	Hippocampus	Hip
121	ProS	ProStriate Area	Posterior cingulate cortex	S_calcarine	CcS
				G_cingul_Post_ventral	PosVCgG
122	PeEc	Perirhinal ectorhinal cortex	Medial temporal cortex	G_oc_temp_med_Parahip	PaHipG
				Pole_temporal	Tpo
123	STGa	Area STGa	Auditory association cortex	G_temp_sup_Lateral	SupTGLp
124	PBelt	ParaBelt complex	Early auditory cortex	G_temp_sup_G_T_transv	HG
				G_temp_sup_Lateral	SupTGLp
				G_temp_sup_Plan_tempo	TPl
125	A5	Auditory 5 complex	Auditory association cortex	G_temp_sup_Lateral	SupTGLp
126	PHA1	ParaHippocampal area 1	Medial temporal cortex	G_oc_temp_med_Parahip	PaHipG
				S_oc_temp_med_and_Lingual	CoS_LinS
127	PHA3	ParaHippocampal area 3	Medial temporal cortex	S_oc_temp_med_and_Lingual	CoS_LinS
128	STSda	Area STSd anterior	Auditory association cortex	S_temporal_sup	SupTS
				G_temp_sup_Lateral	SupTGLp
129	STSdp	Area STSd posterior	Auditory association cortex	S_temporal_sup	SupTS
130	STSvp	Area STSv posterior	Auditory association cortex	S_temporal_sup	SupTS
				G_temporal_middle	MTG
131	TGd	Area TG dorsal	Lateral temporal cortex	Pole_temporal	Tpo
				G_temp_sup_Plan_polar	PoPl
				G_temp_sup_Lateral	SupTGLp
				G_temporal_middle	MTG
132	TE1a	Area TE1 anterior	Lateral temporal cortex	G_temporal_middle	MTG
133	TE1p	Area TE1 posterior	Lateral temporal cortex	G_temporal_middle	MTG
				S_temporal_inf	InfTS
				G_temporal_inf	InfTG
134	TE2a	Area TE2 anterior	Lateral temporal cortex	G_temporal_inf	InfTG
				S_temporal_inf	InfTS
				Pole_temporal	Tpo
135	TF	Area TF	Lateral temporal cortex	S_collat_transv_ant	ATrCoS
				G_temporal_inf	InfTG
				G_oc_temp_lat_fusifor	FuG
136	TE2p	Area TE2 posterior	Lateral temporal cortex	S_oc_temp_lat	LOcTS
				G_temporal_inf	InfTG
				S_collat_transv_ant	ATrCoS
				G_temporal_inf	InfTG
137	PHT	Area PHT	Lateral temporal cortex	G_temporal_middle	MTG
				S_temporal_inf	InfTS
				G_temporal_inf	InfTG
138	PH	Area PH	MT+ complex and neighboring areas	G_temporal_inf	InfTG
				G_and_S_occipital_inf	InfOcG_S
				S_oc_temp_lat	LOcTS
139	TPOJ1	Area TemporoParietoOccipital Junction 1	Temporo-parieto-occipital junction	S_temporal_sup	SupTS
				G_temp_sup_Lateral	SupTGLp
				G_temporal_middle	MTG
140	TPOJ2	Area TemporoParietoOccipital Junction 2	Temporo-parieto-occipital junction	S_temporal_sup	SupTS
				G_temporal_middle	MTG
141	TPOJ3	Area TemporoParietoOccipital Junction 3	Temporo-parieto-occipital junction	S_temporal_sup	SupTS
				G_occipital_middle	MOcG
142	DVT	Dorsal transitional visual area	Posterior cingulate cortex	G_parietal_sup	SupPL
				G_occipital_sup	SupOcG
				S_parieto_occipital	POcS
				S_calcarine	CcS
143	PGp	Area PGp	Inferior parietal cortex	S_temporal_sup	SupTS
				S_oc_middle_and_Lunatus	MOcS_LuS
				G_occipital_middle	MOcG
144	IP2	Area IntraParietal 2	Inferior parietal cortex	S_intrapariet_and_P_trans	IntPS_TrPS
				S_interm_prim_Jensen	JS
				G_pariet_inf_Supramar	SuMarG
145	IP1	Area IntraParietal 1	Inferior parietal cortex	G_pariet_inf_Angular	AngG
				S_intrapariet_and_P_trans	IntPS_TrPS
146	IP0	Area IntraParietal 0	Inferior parietal cortex	S_intrapariet_and_P_trans	IntPS_TrPS
				S_oc_sup_and_transversal	SupOcS_TrOcS
				G_occipital_middle	MOcG
147	PFop	Area PF opercular	Inferior parietal cortex	G_pariet_inf_Supramar	SuMarG
				G_and_S_subcentral	SbCG_S
				G_postcentral	PosCG
148	PF	Area PF complex	Inferior parietal cortex	G_pariet_inf_Supramar	SuMarG
				Lat_Fis_post	PosLS
				G_temp_sup_Plan_tempo	TPl
149	PFm	Area PFm complex	Inferior parietal cortex	G_pariet_inf_Supramar	SuMarG
				S_interm_prim_Jensen	JS
				S_temporal_sup	SupTS
				G_pariet_inf_Angular	AngG
150	PGi	Area PGi	Inferior parietal cortex	S_temporal_sup	SupTS
				G_pariet_inf_Angular	AngG
151	PGs	Area PGs	Inferior parietal cortex	G_pariet_inf_Angular	AngG
				S_temporal_sup	SupTS
152	V6A	Area V6A	Dorsal stream visual cortex	G_occipital_sup	SupOcG
153	VMV1	Ventromedial visual area 1	Ventral stream visual cortex	G_oc_temp_med_Lingual	LinG
154	VMV3	Ventromedial visual area 3	Ventral stream visual cortex	G_oc_temp_med_Lingual	LinG
155	PHA2	Parahippocampal area 2	Medial temporal cortex	G_oc_temp_med_Lingual	LinG
				G_oc_temp_lat_fusifor	FuG
156	V4t	Area V4t	MT+ complex and neighboring areas	G_occipital_middle	MOcG
				G_and_S_occipital_inf	InfOcG_S
157	FST	Area FST	MT+ complex and neighboring areas	S_temporal_inf	InfTS
				G_temporal_inf	InfTG
				S_occipital_ant	AOcS
				G_and_S_occipital_inf	InfOcG_S
158	V3CD	Area V3CD	MT+ complex and neighboring areas	S_oc_sup_and_transversal	SupOcS_TrOcS
				G_occipital_middle	MOcG
				S_oc_middle_and_Lunatus	MOcS_LuS
159	LO3	Area lateral occipital 3	MT+ complex and neighboring areas	S_oc_middle_and_Lunatus	MOcS_LuS
				G_occipital_middle	MOcG
160	VMV2	VentroMedial visual area 2	Ventral stream visual cortex	G_oc_temp_med_Lingual	LinG
161	31pd	Area 31pd	Posterior cingulate cortex	S_subparietal	SbPS
162	31a	Area 31a	Posterior cingulate cortex	S_subparietal	SbPS
163	VVC	Ventral visual complex	Ventral stream visual cortex	G_oc_temp_lat_fusifor	FuG
				G_oc_temp_med_Lingual	LinG
164	25	Area 25	Anterior mid-cingulate and medial prefrontal cortex	S_pericallosal	PerCaS
				G_and_S_cingul_Ant	ACgG_S
165	s32	Area s32	Anterior mid-cingulate and medial prefrontal cortex	G_and_S_cingul_Ant	ACgG_S
				S_suborbital	SbOrS
166	pOFC	posterior OFC complex	Orbital and polar frontal cortex	S_orbital_med_olfact	MedOrS
				G_rectus	RG
				G_orbital	OrG
167	PoI1	Area posterior insular 1	Insular and frontal opercular cortex	Lat_Fis_post	PosLS
				S_circular_insula_inf	InfCirIns
168	Ig	Insular Granular complex	Insular and frontal opercular cortex	S_circular_insula_sup	SupCirInS
169	FOP5	Area frontal opercular 5	Insular and frontal opercular cortex	G_front_inf_Opercular	InfFGOpp
				S_circular_insula_sup	SupCirInS
				Lat_Fis_ant_Horizont	ALSHorp
170	p10p	Area posterior 10p	Orbital and polar frontal cortex	S_front_middle	MFS
				G_and_S_transv_frontopol	TrFPoG_S
				G_and_S_frontomargin	FMarG_S
171	p47r	Area posterior 47r	Inferior frontal cortex	S_orbital_lateral	LORs
				G_front_inf_Orbital	InfFGOrp
				G_front_middle	MFG
				G_orbital	OrG
172	TGv	Area TG ventral	Lateral temporal cortex	Pole_temporal	Tpo
				G_temporal_inf	InfTG
173	MBelt	Medial belt complex	Early auditory cortex	Lat_Fis_post	PosLS
				G_temp_sup_G_T_transv	HG
				S_circular_insula_inf	InfCirIns
174	LBelt	Lateral belt complex	Early auditory cortex	Lat_Fis_post	PosLS
				S_temporal_transverse	TrTs
				G_temp_sup_Plan_tempo	TPl
175	A4	Auditory 4 complex	Auditory association cortex	G_temp_sup_Plan_tempo	TPl
				G_temp_sup_Lateral	SupTGLp
176	STSva	Area STSv anterior	Auditory association cortex	S_temporal_sup	SupTS
177	TE1m	Area TE1 middle	Lateral temporal cortex	G_temporal_middle	MTG
				S_temporal_inf	InfTS
178	PI	Para-insular area	Insular and frontal opercular cortex	S_circular_insula_inf	InfCirIns
179	a32pr	Area anterior 32 prime	Anterior mid-cingulate and medial prefrontal cortex	G_and_S_cingul_Ant	ACgG_S
				G_and_S_cingul_Mid_Ant	MACgG_S
180	p24	Area posterior 24	Anterior mid-cingulate and medial prefrontal cortex	S_pericallosal	PerCaS
				G_and_S_cingul_Ant	ACgG_S
				G_and_S_cingul_Mid_Ant	MACgG_S
N/A	N/A	N/A	N/A	Cerebellar cortex	Cerebellum_Cortex
				Thalamus proper	Thalamus_Proper
				Caudate nucleus	Caudate
				Putamen	Putamen
				Pallidum	Pallidum
				Amygdala	Amygdala
				(Nucleus) Accumbens area	Accumbens_area
				Cerebellar cortex	Cerebellum_Cortex
				Thalamus proper	Thalamus_Proper
				Caudate nucleus	Caudate
				Putamen	Putamen
				Pallidum	Pallidum
				Amygdala	Amygdala
				(Nucleus) Accumbens area	Accumbens_area
AAN Name	AAN description	AAN hemisphere name			AAN hemisphere
DR	Dorsal raphé nuclei	DR			B
LC	Locus coeruleus	L_LC			L
LC	Locus coeruleus	R_LC			R
MRF	Mesencephalic reticular formation	L_MRF			L
MRF	Mesencephalic reticular formation	R_MRF			R
MR	Median raphé nuclei	MR			B
PAG	Periaqueductal gray	PAG			B
PBC	Parabrachial complex	L_PBC			L
PBC	Parabrachial complex	R_PBC			R
PO	Pontis oralis	L_PO			L
PO	Pontis oralis	R_PO			R
PPN	Pendunculopontine nucleus	L_PPN			L
PPN	Pendunculopontine nucleus	R_PPN			R
VTA	Ventral tegmental area	VTA			B

### DIABLO analysis

Data were split into training and testing datasets (“Supplementary Information: Training and Testing Sets”). A data integration analysis for biomarker discovery using latent components (DIABLO) was conducted to achieve our study aims of predicting groups of improvers vs. non-improvers at 3 and 12 months. The method identifies a limited number of correlated variables from multiple datasets to predict outcome. In this study, the outcome was “symptom status”. The method is an extension of sparse generalized canonical correlation analysis [46], which is a generalization of partial least square for multiple matching data sets (*Q*), to a supervised learning framework [26, 47]. Before proceeding with DIABLO analysis, individual sPLS models were run between pairwise datasets (e.g., morphometric and clinical; morphometric and resting state) to understand major sources of variation in each dataset, and guide the integration process by obtaining correlations to employ in a data-driven weighted design matrix [26]. The design matrix is a *Q* × *Q* matrix representing *if and by how much* each dataset should be correlated for the model’s algorithms in the DIABLO analysis. Values range from 0 to 1. The design matrix was created by taking the correlated values of the first component from each individual sPLS model (Supplementary Information, Figs. 2–13). All correlations were above 0.8; therefore, values were set to 1 in the design matrix **(**Supplementary Information: Supplemental Fig. 1) [26]. Once the design matrix was determined, A DIABLO model with five components was first fit without any variable selection, and global performance was assessed using leave-one-out-cross validation (LOOCV). The number of components chosen based on the lowest balanced error rate (BER) and distance metric (maximum distance vs centroids distance vs. mahalanobis distance) across a number of components. After determining the number of components to use, the optimal number of variables to be kept per component, one component at a time, by defining a grid of values—in this case from 2 to 300—for each component. LOOCV was run with the distance metric defined above to give the lowest BER. The classification error rate was then extracted averaged across every LOOCV model for each tested grid value. The optimal number of components, and features per component was then extracted. Since this process may lead to overfitting, manual tuning of the number of features per component was then conducted to get the lowest balanced error rate and best accuracy on a holdout testing dataset. The main output measures for DIABLO are a set of components (i.e., latent variables) chosen in the model, a set of loading vectors (i.e., coefficients assigned to each variable to define each component), and a list of selected variables from each dataset and associated to each component. Loadings are the coefficients assigned to each variable to define each component, and their absolute value represents the importance of each variable in DIABLO. It is important to note that each loading vector is assigned to a particular component, and the loading vectors are obtained so that the covariance between a linear combination of *X* variables and *Y* is maximized. Individual sample plots represent each individual projected onto a space that is defined by the components. The coordinates for each individual are determined by their component values/scores. Loading plots help visualize each coefficient (i.e., importance) assigned to the variables in each component of each dataset. Circos diagrams are built on a similarity matrix [48] and represent the correlation between variables from different datasets, and a cutoff was chosen as *r* = 0.7 as this is universally considered a “strong” correlation. Relevance networks are graphs where the nodes represent the variables chosen by DIABLO and the edges represent variable associations. Edges between nodes were only drawn if the association was 0.7 or higher. Nodes/variables extracted from DIABLO that did not have any associations were not represented on the network. The area under the receiver operating characteristic (ROC) curve was calculated for each dataset separately by component, and *p*-values were calculated using the Wilcoxon test comparing improvers vs. non-improvers. The area under the ROC curve is a way to summarize the overall diagnostic accuracy of the model. The values range from 0 to 1, where 1 represents a perfectly accurate model. A value of 0.5 represents no discriminatory ability. Values between 0.7 and 0.8 are considered acceptable, while between 0.8 and 0.9 is considered excellent, and greater values are considered outstanding [49]. The model was then tested on the testing data and peformance assessed with the BER, along with confusion matrix statistics such as sensitivity, specificity and the F1 statistic.

## Results

See Supplementary Information, Tables 2 and 3 for descriptive statsitics. An independent *t*-test was done to confirm that age was similar in the train (*M* = 28.96, SD = 10.97) and test (*M* = 29.64, SD = 14.04) datasets (*t* = −0.15, *p* = 0.88, *d* = 0.06).

### Symptom change validation across 12-months

To understand the rate of improvement, 24 participants (40%) showed improvement after 3 months and 17 participants (40%) improved after 12 months. To validate that the 12-month improvers were improving over 12 months compared to non-improvers, the percentage of timepoints (3, 6, 9, and 12 months) where subjects “improved” (e.g., decreased on the IBS-SSS by 50 points) from baseline was first calculated. Over 12 months, compared to non-improvers (*M* = 22%, SD = 23%), improvers (*M* = 71%, SD = 22%) had a much higher percentage of time points showing improvement (*t*_(41)_ = 6.92, *p* < 0.001, *d* = 2.18). Of the 17 patients that improved after 12 months, 11 of them improved after 3 months. In the 26 patients that did not improve after 12 months, eight patients improved after 3 months.

### DIABLO results

DIABLO successfully identified a correlated ‘omics signature from baseline using multimodal imaging and clinical and behavioral assessments by classifying groups of improvers and non-improvers at 3 and 12 months. Based on the balanced error rate (i.e., the average proportion of wrong classifications in each class validated via LOOCV in the training sample), three and two components were selected for the 3 and 12 month models, respectively. Following feature tuning and selection, and validation of the final model with LOOCV, both models achieved an AUROC over 0.88 (*p* < 0.05). (Supplementary Information, Tables 3 and 4).

For the DIABLO model predicting three-month symptom changes, the tuning process on the training set identified a multiomics signature of three components. Component one had five morphometry, four anatomical connectivity, four resting-state functional connectivity, and two clinical features. Component two had four morphometry, three anatomical connectivity, three resting-state functional connectivity, and three clinical features. Component three had two morphometry, two anatomical connectivity, three resting-state functional connectivity, and three clinical features.

For the DIABLO model predicting 12-month symptom changes, the signature was composed of two components. Component 1 had four morphometry, five anatomical connectivity, three resting-state functional connectivity, and three clinical features. Component 2 had two morphometry, three anatomical connectivity, two resting-state functional connectivity, and four clinical features.

**Fig. 1:**
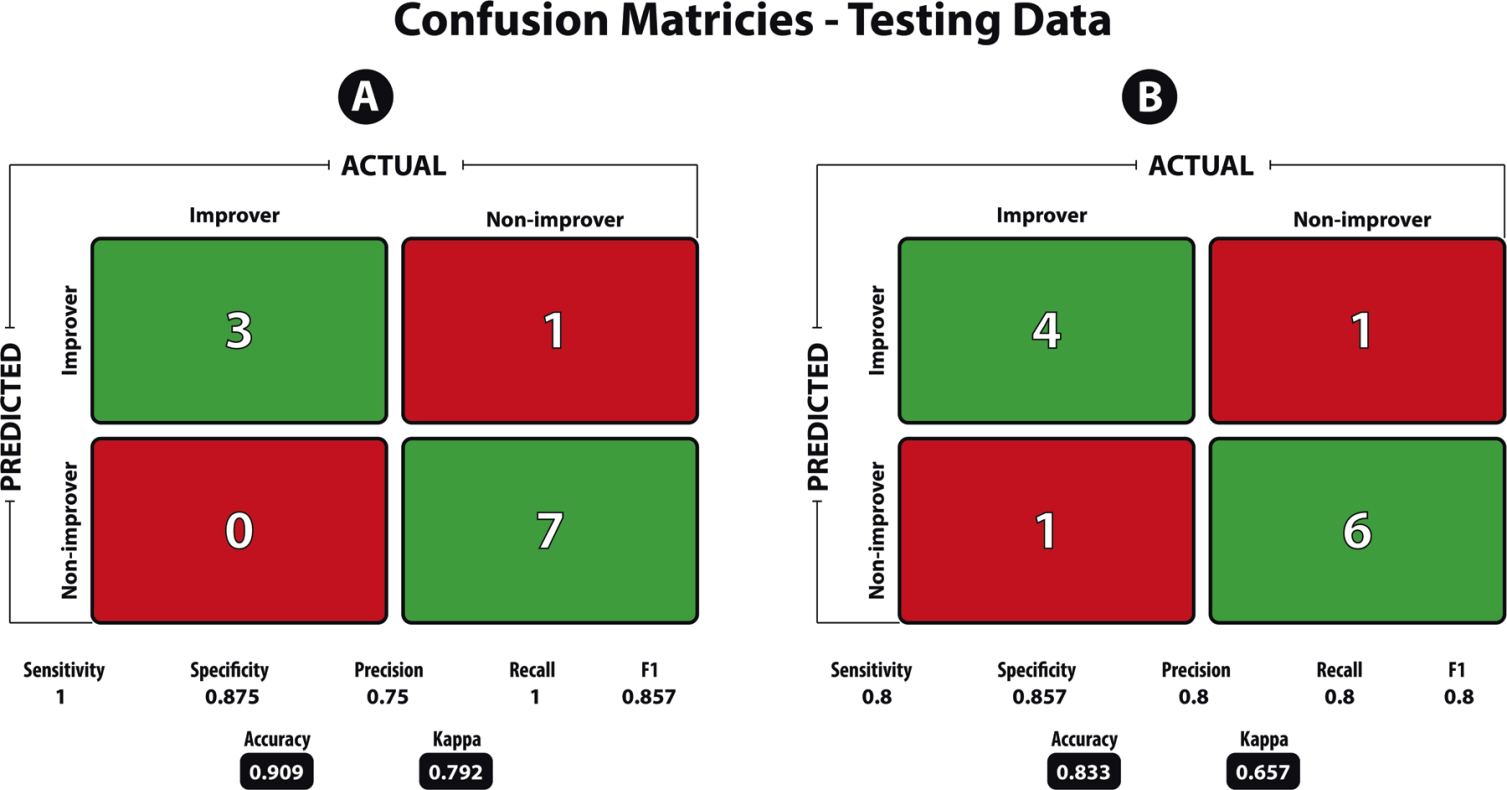
Confusion matrix statistics on the testing dataset for the model predicting 3-month and 12-month improvers and non-improvers. Sensitivity and recall are defined as the true positive rate (i.e., number of predicted improvers divided by the total number of improvers), Specificity is defined as the true negative rate (i.e., number of predicted non-improvers divided by the total number of non-improvers). Precision is the ability of the classifier to not label a true negative as a positive (i.e., the ability to not label a non-improver an improver). The *F*1 score is the harmonic mean of precision and recall, with values closer to 1 being a better score. Accuracy is defined as the number of true positives and true negatives divided by the total population. The Kappa statistic is known to be a better measure compared to accuracy, especially in the case of imbalanced classes. Kappa values between 0.61 and 0.80 are said to be “Substantial” and between 0.81 and 1.0 to be “Almost Perfect”.

Areas under the ROC curve (AUROC) by data type, and of the final DIABLO model show high classification accuracy (Supplementary Information, Tables 3 and 4). Predictions and confusion matrix statistics were then assessed using the final model on an external test dataset (Fig. 1).

### Prediction of 3-month improver model based on external test set and contributing features

**Fig. 2:**
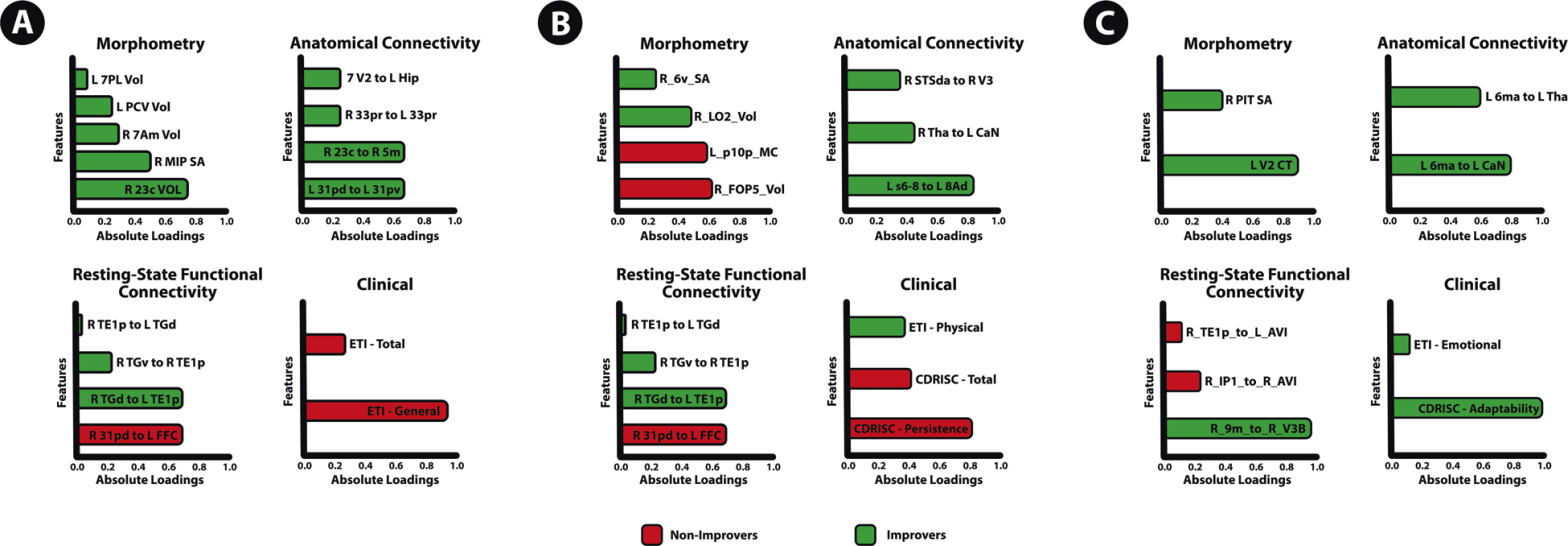
Features contributing to the multi-modal neuropsychosocial signature predicting improvers vs. non-improvers in IBS symptoms after 3 months. Absolute loadings depict the relative importance of each feature. Colors represent which group has the higher mean value for that feature. A Component 1 *Morphometry*: 23c Area 23C (posterior cingulate cortex [PCC]), MIP medial intraparietal area; 7 Am medial area 7A (SPC); PCV precuneus, 7 PL lateral area 7P (superior parietal cortex [SPC]). *Anatomical Connectivity*: 31pd area 31pd (PCC), 31pv area 31p ventral (PCC), 23c area 23c (PCC), 5 m area 5 m (paracentral lobule); 33pr area 33 prime, V2 second visual area, Hip Hippocampus. *Resting-State Functional Connectivity*: 31pd area 31pd (PCC), FFC fusiform face cortex, TGd area TG dorsal (LTC), TE1p area TE1 posterior (LTC), TGv area TG ventral. *Clinical*: ETI general early trauma inventory general score, ETI total early trauma inventory total score B: Component 2 Abbreviations: *Clinical*: CDRISC persistence Connor-Davidson resilience persistence subscale, CDRISC total Connor-Davidson resilience persistence total score, ETI physical early trauma inventory physical score. *Morphometry*: FOP5 area frontal opercular 5, p10p area posterior 10p, LO2 area lateral occipital 2; 6 v ventral area 6. *Anatomical connectivity*: s6–8 superior 6–8 transitional area, 8Ad area 8Ad, Tha. Thalamus, CaN Caudate nucleus, STSda area STSd anterior, V3 third visual area. *Resting-State Functional Connectivity:* IPS1 intraparietal sulcus area 1, V4 fourth visual area, PFt area PFt, area OP1 area OP1/SII, PFop area PF opercular, OP4 area OP4/PV C: Component 3 Abbreviations: *Clinical*: CDRISC adaptability Connor-Davidson resilience adaptability subscale, ETI emotional early trauma inventory emotional score. *Morphometry*: V2 second visual area, PIT posterior inferotemporal cortex. *Anatomical Connectivity*: 6 ma area 6m anterior, CaN Caudate nucleus, Tha Thalamus. *Resting*-*State Functional Connectivity*: 9 m area 9 middle, IP1 area intraparietal 1, AVI anterior ventral insular area, TE1p area TE1 posterior.

**Fig. 3:**
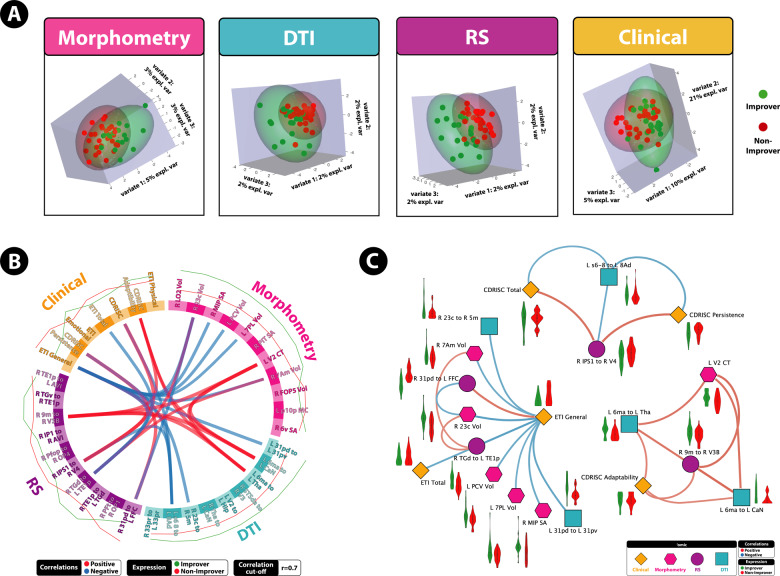
A highly correlated multi-modal, brain-clinical signature predicts 3 month IBS symptom trajectories via DIABLO. **A** Sample plots for morphometry, anatomical connectivity, resting-state functional connectivity, and clinical variables for the 3 month DIABLO analysis. Samples are represented as points according to their projection across three latent variables. **B** Circos plot for the 3 month DIABLO analysis representing all of the features in the DIABLO model, and the correlations between variables of different data types. Correlation cut-off is set to *r* = 0.7. Lines along the outside of the circle represent the mean “expression” levels. Greater levels are in accordance to the line being farther away from the circle. **C** Relevance network for the 3 month DIABLO analysis representing the correlation between variables of different data types. Red lines represent positive correlations and blue lines represent negative correlations. Boxplots with violin plots represent the distribution of each group for each feature. Correlation cut-off is set to *r* = 0.7.

The 3-month DIABLO model on training data was used to predict if groups of patients can be classified as improvers or non-improvers on an independent testing dataset consisting of multimodal brain and clinical data. The model predicted classes on the test dataset with 91% accuracy and *F*1 = 0.85 (Fig. 1A). Features that contribute to the model, and the group expressing the maximal value on components 1–3 can be seen in Fig. 2A–C. Sample plots in the three component space for each data type can be seen in Fig. 3A. The circos diagram in Fig. 3B shows all of the features of importance selected by DIABLO, which features are greatly correlated, and the relative mean values of each feature in each group. The relevance network in Fig. 3C shows only the variables that are highly correlated with each other for easier viewing, along with box-violin plots showing the distribution of each feature per group. “Supplementary Information: Distribution of Selected Markers by DIABLO—3 months”.

On component 1, the morphometry features consisted of surface area and volume of the superior parietal cortex and the posterior cingulate (PCC), both hubs of the default mode network. The anatomical connectivity features consisted of connectivity within the PCC, between the PCC and paracentral lobular cortex, between the bilateral aMCC, and between the hippocampus and visual cortex. The rs-FC features consisted rs-FC within and between the lateral temporal cortices of the default mode network and between the PCC and fusiform face gyrus. Clinical features consisted of the general subscale and total score of the early life trauma questionnaire.

On component 2, the morphometry features consisted of volume of the anterior insula (aINS), mean curvature of the orbitofrontal cortex (OFC), volume in the lateral occipital cortex and surface area in the premotor cortex. Anatomical connectivity features consisted of connectivity within the dorsolateral prefrontal cortex, between the thalamus and caudate nucleus, and between the third visual area and auditory association cortex. The rs-FC features consisted of rs-FC within the visual cortex, between the inferior parietal cortex and secondary somatosensory cortex, and between the subcentral gyrus and operculum. Clinical features included the CD-RISC Persistence and total scales, and lower scores on the ETI physical subscale.

On component 3, the morphometry features consisted of volumes in the second visual area and posterior inferotemporal complex. Anatomical connectivity features consisted of connectivity between the supplementary motor area (SMA) and caudate nucleus, and between the SMA and thalamus. The rs-FC features included rs-FC between the medial prefrontal cortex (mPFC) and visual cortex and between the anterior insula and lateral temporal cortices. Clinical features included the CMSI—adaptability scale and ETI—emotional scale.

### Prediction of 12-month improver model based on external test set and contributing features

**Fig. 4:**
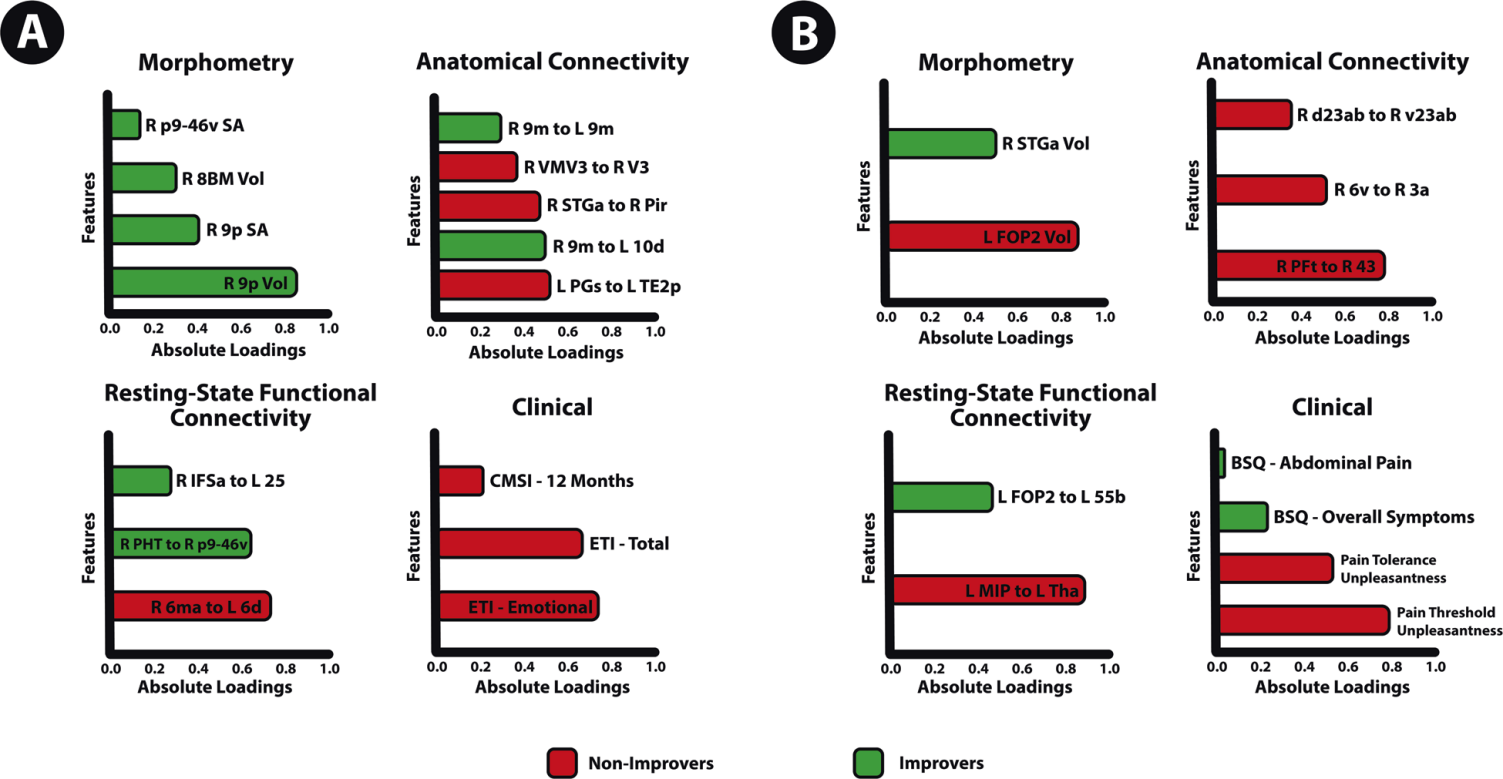
Features contributing to the multi-modal neuropsychosocial signature predicting improvers vs. non-improvers in IBS symptoms after 12 months. Absolute loadings depict the relative importance of each feature. Colors represent which group has the maximal mean value for that feature. Component 1 *Abbreviations: Morphometry:* 9p area 9 posterior (dlPFC), 8BM area 8BM (mPFC), p9-46v area posterior 9-46v (dlPFC). *Anatomical Connectivity:* 9 m area 9 middle (medial prefrontal cortex [mPFC]), VMV3 ventromedial visual area 1, V3 third visual area, STGa area STGa, Pir Pirform cortex (anterior insula [aINS]), 10d area 10d (OFC), PGs area PGs; TE2p area TE2 posterior *Resting-State Functional Connectivity:* 6 ma area 6m anterior, 6d dorsal area 6, PHT area PHT, p9-46v area posterior 9-46v, IFSa area IFSa, 25 Area 25 *Clinical:* ETI total early trauma inventory total score, ETI emotional early trauma inventory emotional subscale score, CMSI 12 months complex multi-symptom inventory in the past 12 months. Component 2: *Abbreviations*: *Morphometry*: FOP2 frontal opercular area 2; STGa area STGa *Anatomical Connectivity:* PFt area Pft; 43 area 43; 6v ventral area 6, d23ab area dorsal 23 a + b, v23ab area ventral dorsal a + b *Resting-State Functional Connectivity:* MIP medial intraparietal area, Tha Thalamus, FOP2 frontal opercular area 2, 55b area 55b *Clinical*: pain threshold unpleasantness, pain tolerance unpleasantness; BSQ overall symptoms Bowel Symptom Questionnaire (overall symptom score), BSQ abdominal pain Bowel Symptom Questionnaire (overall abdominal pain).

**Fig. 5:**
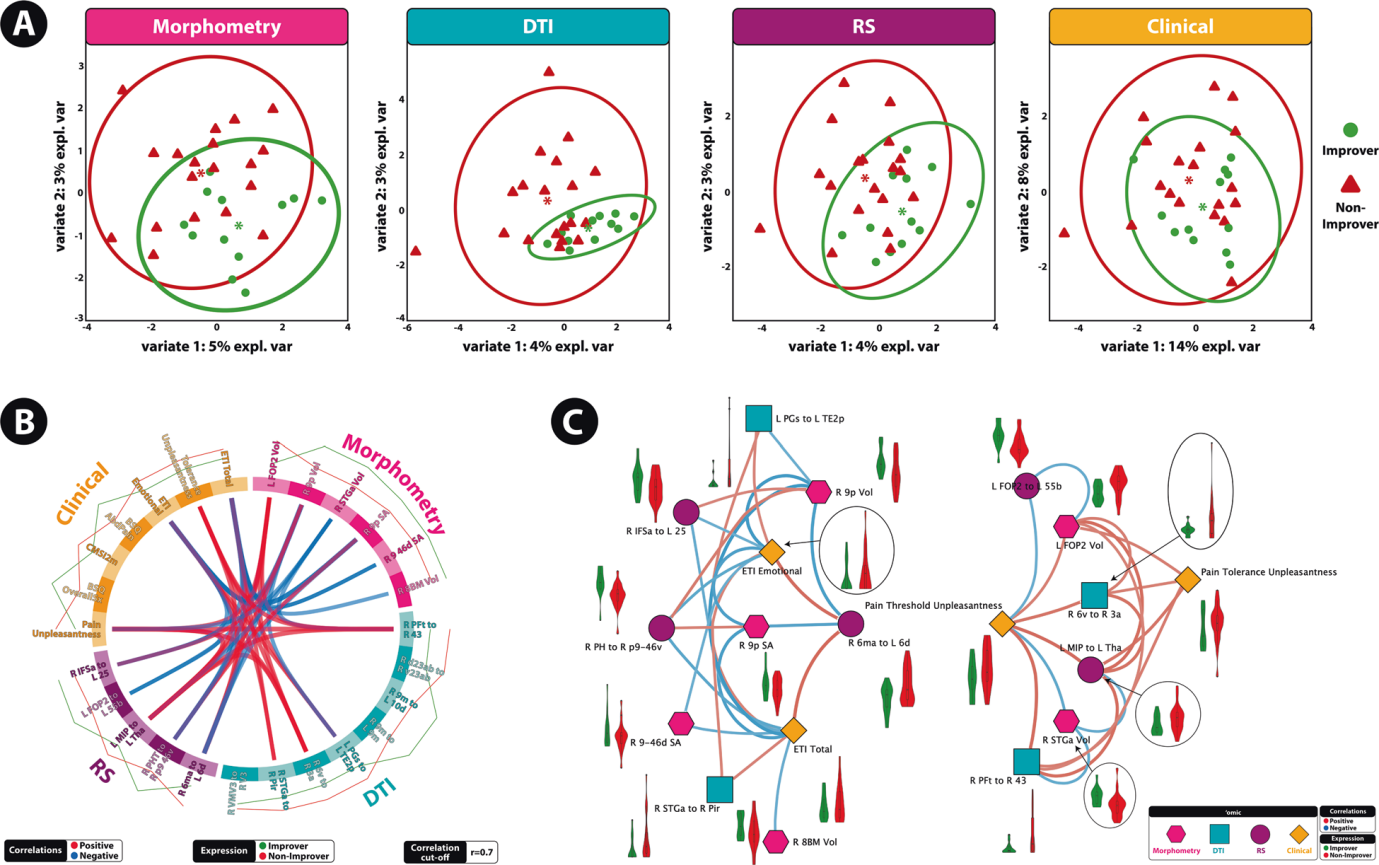
A highly correlated multi-modal, brain-clinical signature predicts 12 month IBS symptom trajectories via DIABLO. **A** Sample plots for morphometry, anatomical connectivity, resting-state functional connectivity, and clinical variables for the 12-month DIABLO analysis. Samples are represented as points according to their projection across two latent variables. Explained variance across each component is listed. **B** Circos plot for the 12-month DIABLO analysis representing all of the features in the DIABLO model, and the correlations between variables of different data types. Correlation cut-off is set to *r* = 0.7. Lines along the outside of the circle represent the mean “expression” levels. Greater levels are in accordance to the line being farther away from the circle. **C** Relevance network for the 12-month DIABLO analysis representing the correlation between variables of different data types. Red lines represent positive correlations and blue lines represent negative correlations. Boxplots with violin plots represent the distribution of each group for each feature. Correlation cut-off is set to *r* = 0.7.

The tuned 12-month DIABLO model on the training data was used to predict if patients can be classified as improvers or non-improvers on an independent test dataset consisting of multimodal brain and clinical data. The model predicted classes on the test dataset with 83% accuracy and *F*1 = 0.80 (Fig. 1B). Features that contribute to the model, and the group expressing the maximal value on components 1–2 can be seen in Fig. 4A, B. Sample plots in the 2 component space for each data type can be seen in Fig. 5A. The circos diagram in Fig. 5B shows all of the features of importance selected by DIABLO, which features are greatly correlated, and the relative mean values of each feature in each group. The relevance network in Fig. 5C shows only the variables that are highly correlated with each other for easier viewing, along with box-violin plots showing the distribution of each feature per group. All distributions are also available in the “Supplementary Information: Distribution of Selected Markers by DIABLO—12 Months”.

On component 1, morphometry features consisted of surface area and volume in the dlPFC and medial prefrontal cortex (mPFC). Anatomical connectivity features consisted of connectivity between the left and right mPFC, and between the OFC and mPFC, between the angular gyrus and lateral temporal cortex, the anterior insula and lateral temporal cortex, within the visual cortex, between the mPFC and OFC, and within the mPFC. The rs-FC features consisted rs-FC between the SMA and premotor cortex, between the lateral temporal cortex and dlPFC, and inferior frontal cortex and subgenual ACC. Clinical features consisted of the ETI Emotional subscale, ETI total score, and the CMSI—12 months scale.

On component 2, morphometry features included volume of the superior temporal gyrus, and volume of the anterior insula. Anatomical connectivity features consisted of connectivity between the inferior parietal cortex and subcentral area, between the premotor cortex and central sulcus, and within the posterior cingulate. The rs-FC features included rs-FC between the superior parietal cortex and thalamus, and between the aINS and premotor cortex. Clinical features consisted of pain threshold, tolerance and unpleasantness ratings, BSQ overall symptoms and abdominal pain.

## Discussion

### A neuropsychosocial signature predicts improvement in patients with IBS after 3 and 12 months

With past research on pain perception and attentional systems [10, 50–52], these results suggest attentional systems are not able to focus away due to a greater signaling and salience of visceral nociception and perception, and lower capability of the default mode network to help take attention away from the present sensory world [52, 53]. Therapies that target these systems, such as cognitive-behavioral therapy and practicing mindfulness [54] may be good candidates for more comprehensive IBS treatments. Specific findings within the DIABLO signature support this hypothesis.

Results showed IBS participants that did not improve in symptom severity over 12 months had lower morphological integrity, anatomical connectivity and resting-state functional connectivity within the default mode network, and reported more early life trauma. They also had lower connectivity in the dlPFC, which is responsible for inhibitory control systems, and were associated with greater persistence scores. These may be indicative of more stressful and traumatic life histories. Sensorimotor and salience systems were more greatly connected and active in patients who did not improve, and positively associated with ratings of experimental pain threshold unpleasantness. The combination of these findings indicates that attentional systems may be compromised to disengage from visceral sensations. Thus, an increase in salience and sensorimotor processing with an increased affective response to pain, and early life trauma may contribute to persistence of pain. DIABLO indicated that a multivariate signature composed of latent variables of structural, anatomical connectivity, resting-state functional connectivity and clinical/behavioral data can predict longitudinal symptom improvement in patients with IBS with 88–91% accuracy.

IBS participants whose symptoms improved—based on a 50 point decrease on the IBS-SSS from baseline after both follow-ups—had distinct neuropsychosocial patterns distinguishing them from participants with persistent or worsening symptoms. The pertinent multimodal features in each DIABLO model, from all modalities, involved key regions of the default mode network (DMN) including the posterior cingulate cortex (PCC) and dorsomedial prefrontal cortex (dmPFC), each are involved in pain perception [50, 53]. These key hubs are part of the “dynamic pain connectome”, and are *activated* when attention is engaged with thoughts away from present sensory stimuli and engaged in mind wandering (i.e., thoughts unrelated to the present sensory environment) [52, 53, 55]. Results indicated that regardless of short-term or long-term improvement, greater early-life trauma was associated with lower morphological integrity, anatomical connectivity, and resting-state functional connectivity within the DMN. Conversely, these hubs are *deactivated* when attention is placed on painful sensations [55]. The lower amount of structural integrity, anatomical connectivity, and resting-state functional connectivity within the DMN may represent deficits in disengagement from unpleasant visceral sensations. These deficits are associated with greater amounts of early life adversity (ELA), which is a risk factor for IBS [13, 56–58]. Children and adolescents with IBS have reduced gray matter and greater resting-state connectivity across networks associated with altered pain sensitivity [59, 60]. This study’s results are consistent with previous research, demonstrating that attentional systems may be less prone to mind-wandering, which has been shown to be a key mechanism of pain inhibition [50, 55]. Past research investigating the default mode network in patients with IBS, has shown rectal lidocaine treatment increases coherence in the default mode network and decreases perceived pain [61], further supporting the current results on how inherent differences and associations with behavioral data can predict IBS symptom trajectory.

There were differences in signatures between short-term vs. long-term predictions. Pertinent discriminations for classifying 3-month improvers were aspects of CDRISC defined resilience. Scores for the CDRISC Persistence subscale were higher in patients without improvement and associated with lower anatomical connectivity within the dlPFC, as well as greater resting-state functional connectivity in circuits responsible for transmitting top-down spatial attention information to the visual cortex during sustained attention [62]. Further, over-engagement of attentional systems and lower inhibitory control from the dlPFC points to more severe traumatic and stressful life histories, as indicated by resilience subscale scores in non-improvers [63, 64]. Mounting evidence has shown the dlPFC acts as an interface between cognitive processing and pain regulation, shows a loss of neuronal tissue with chronic pain which is at least partially reversible [65**–**67]. This identifies the dlPFC as a potential therapeutic target in IBS, using transcranial magnetic stimulation, transcranial direct-current stimulation and mindfulness, all of which have shown promise in other chronic pain populations [65].

Conversely, patients with improvements had greater scores on the CDRISC Adaptability subscale, which was associated with greater default mode resting-state connectivity and corticospinal tract integrity. The hyperconnectivity of these pathways originating in the motor cortex may constitute strengths in active top-down pain modulation. As seen in the current results, stimulation from the motor cortex can result in top-down activation via the thalamus and basal ganglia, can result in activation of the periaqueductal gray (PAG) for pain modulation, which may be a modulatory mechanism pain-relieving in the current study that requires further investigation [66]. Stimulating the motor areas in transcranial direct current stimulation has also been found to reduce pain [67]. This suggests that the brain in improvers was more “adaptative”, and was better able to minimize attention to IBS symptoms and more likely to engage in top-down pain modulation via pyramidal tracts.

Pertinent behavioral variables discriminating long-term improvers included pain threshold and tolerance unpleasantness ratings from sensory testing. Greater unpleasantness ratings were associated with greater anatomical connectivity between the anterior portion of the subcentral gyrus (area 43) and posterior bank of the postcentral sulcus (area Pft)—two regions that are known to be distinctly connected via white matter projections, connected to the somatosensory strip, and play a large role in processing somatosensory information [68]. Greater unpleasantness ratings were also associated with greater S1-SMA anatomical connectivity. All pertinent features discriminating long-term improvers were associated with greater resting-state connectivity between the thalamus and superior parietal cortex, and with greater volume in the posterior insula (SMN), a brain region receiving afferent inputs from the viscera, referred to as viscerosensory cortex [69]. This finding supports the hypothesis that circuitry in sensorimotor systems is more robust, connected and active in IBS patients with persistent or worsening symptom trajectories. Pain has historically been considered a bidimensional experience consisting of the sensory-discriminative dimension (often referred as “intensity”), and the affective-motivational (or “unpleasantness”) dimension [70, 71]. The ascending and descending pain modulation pathways underlying the differences between pain intensity and pain unpleasantness have been well explored, showing that pain intensity is the primary contributor to pain unpleasantness and not vice-versa [72]. Our results consisted of regions known to interact to produce pain intensity, unpleasantness and secondary pain affect [72, 73]. The anterior cingulate cortex (ACC) is known to play a large role in the modulation of unpleasantness [72, 73] but was not identified in the signature by DIABLO. We posit that as the ACC is involved in encoding immediate pain unpleasantness, and our baseline imaging was not conducted in conjunction with an acute pain stimulus, our protocol instead identified brain structures and pathways that are generally responsive to sensations, arousal, autonomic, somatomotor activation, and perceived threat *which underlie pain unpleasantness* rather than the immediate threat of the pain stimulus [72].

Study outcomes also found synergistic effects in brain regions known to be affected by early adverse life events. The greater resting-state functional connectivity between the PCC and fusiform face cortex and its positive association with early life trauma is supported by past research. The fusiform face cortex is structurally connected to the inferior longitudinal fasciculus (ILF), which is known as the visual-limbic pathway [74], and trauma such as domestic violence is associated with decreased integrity of the ILF in the developing brain [75, 76]. Regions connected to the ILF, such as the superior temporal gyrus also had lower volume [67]. Non-improvers also had lower cortical thickness in the second visual area (V2)—also shown to have lower integrity in the abused developing brain [74]—and lower anatomical connectivity between the V2 and hippocampus, which are connected via the ILF. This pattern was accentuated with a greater lifetime history of symptoms that accompany chronic pain, and the ILF is known to have decreased integrity associated with greater symptom severity in other chronic pain populations and the transition to chronic pain [24, 76]. Regions at the anterior end of the ILF such as the hippocampus have been shown to play a key role in the transition from acute to chronic pain [77]. Additionally, brain regions along the ILF that are known to be associated with early-life adversity such as the hippocampus, fusiform face cortex, and visual cortex were also identified in the multi-modal signature. This indicates that neuroplastic changes in the ILF due to early life adversity may be involved in the exacerbation of IBS. For example, areas such as the hippocampus and immediate surrounding regions are known to play a crucial role in developing chronic pain, as hippocampal reorganization is associated with the transition from acute to chronic pain via abnormal learning and emotional processes [77]. Specifically in IBS, abnormal hippocampal glutamatergic transmission [78] and activation of the hippocampus in response to rectal distention [79] have also been observed. Taken together, neuroplastic changes in the ILF and connected brain regions may accentuate the risk for exacerbating IBS symptoms, highlighting the need for preventative intervention in early life when the first symptoms indicative of chronic pain development can be detected.

### Limitations and advantages

The relatively small sample size should be noted as a limitation for the current study; this was due in part to participant attrition at follow-up. Furthermore, because IBS is a life-long illness for some, the study’s 12-month follow-up provides only a snapshot of each patients’ symptom trajectory. The literature provides evidence that early adverse life events, [10] are associated with increased risk of exacerbating IBS; yet, symptom trends over 12 months, as indicated by participants’ self-reports, does not establish causation. A comparison of healthy volunteers or another visceral pain control such as inflammatory bowel disease or interstitial cystitis (IC)/bladder pain syndrome (BPS) would provide further insight into pain-related, brain mechanistic pathways [80]. Functional life impairment, and the impact of daily life due to symptoms were not assessed but would be important in future studies and analyses [20]. Additionally, as this is a difficult population sample to recruit, the time-of-day measures that were assessed can vary from 8 AM to 6 PM. As circadian rhythms can be seen in virtually every physiological process in the body, including the central nervous system, and seen in many brain-related disorders [81, 82], future studies should consider integrating circadian rhythms into the study design and analysis. As one would expect stress, anxiety and depression to be pertinent variables of interest, the reason these likely were not identified by DIABLO is the participants recruited did not have and diagnosable psychiatric conditions. This would result in a limited range of mood variables. Additionally, the short nature of the follow-up in this long-term illness is likely leading to the identification of specific types of predictors. Future studies should also monitor hormonal changes at different phases in the menstrual cycle at the time of testing. Menstrual cycle status can be estimated in our sample by calculating the number of days between the first day of their menstrual period and the day of scan (nine women were in the menstrual phase, 13 in the follicular phase, and 27 in the luteal phase of the data available), but without ovulation kits, exact status is unknown. Strengths of the study included measuring symptom changes longitudinally over 12 months, as opposed to a cross-sectional study. This can also be further strengthened by including multi-modal brain imaging at multiple time points, along with other potentially related biomarkers from areas such as the microbiome, genetics and the immune system. This is also the first study to use multi-modal brain imaging and clinical data in an integrative manner to predict symptom trajectory in a sample of patients with IBS.

## Supplementary information

Supplemental Material

## Data Availability

All analyses after data processing were done using the mixOmics package [47] version 6.17.27 in R version 4.1.0. Code and data are available on request.
