# Supplementary material for: A neuropsychosocial signature predicts longitudinal symptom changes in women with irritable bowel syndrome
Source: Mol Psychiatry. 2021 Nov 24;27(3):1774–91. doi: 10.1038/s41380-021-01375-9 (PMC9095468; doi:10.1038/s41380-021-01375-9)
Supplement: Supplementary file 1 — Supplemental Material [file 41380_2021_1375_MOESM1_ESM.docx]

**Supplementary information**

**Supplementary Methods**

*Study Design*

The study design for the full deep phenotyping study is given in the table below.

| Study Procedures | Screening (Visit 1) | MRI (Visit 2) | Follow-Up MRI (Visit 3 & 4 at 3 & 12 months |
| --- | --- | --- | --- |
| **Read and sign informed consent** | X |  | As needed |
| **Assess eligibility** | X |  | X |
| **Review concomitant medications** | X | X | X |
| **Weight and vital signs** | X |  | X |
| **Medical history and physical exam** | X |  |  |
| **Psychological interview (MINI+)** | X |  |  |
| **Blood sample** | X |  | X |
| **Review adverse events** |  | X | X |
| **Thermal pain testing** | X |  | X |
| **BIA measure (HC only)** | X |  |  |
| **Online SurveyMonkey questions (Psychosocial Questionnaires)** | X | X | X |
| **fMRI resting-state scans** |  | X | X |
| **Structural MRI** |  | X | X |
| **Diffusion MRI** |  | X | X |
| **MR spectroscopy scans** |  | X | X |
| **Urine pregnancy test** | X | X | X |
| **Stool samples (IBS only)** |  | X | X |

*Questionnaires*

The IBS Symptom Severity Scale (IBS-SSS) was used as the primary measure of symptom severity, which is shown to have good reproducibility and is sensitive to change. It assesses pain, distention, bowel dysfunction, and quality of life.^1^ The Bowel Symptom Questionnaire (BSQ) is a validated questionnaire assessing self-reported gastrointestinal symptom severity, bloating, and abdominal pain on a 20 point scale.^2^ The Hospital Anxiety and Depression Scale (HADS) is a reliable instrument in detecting states of depression and anxiety in an medical outpatient clinic setting.^3^ The State-Trait Anxiety Inventory (STAI) is a 40-item self-report questionnaire deigned to assess both state (20 questions) and trait anxiety (20 questions) in adults.^4^ The Perceived Stress Scale (PSS) is a widely used psychological questionnaire to measure stress perception. It is a 10 question survey that measures the degree life situations are considered appraised as stressful.^5^ The International Personality Item Pool (IPIP) was used to measures personality factors of neuroticism and extraversion.^6^ The Early Trauma Inventory (ETI) is a semi-structured interview assessing the four domains of physical, emotional, sexual abuse and general traumatic experiences. It then addresses the most serious trauma with an additional question.^7^ The Adverse Child Experiences (ACE) is a validated scale that has been used to assess exposure to adversity before the age of 18 and associated later health outcomes. The 10-item questionnaire assess history of emotional, physical or sexual abuse, childhood neglect, and household dysfunction.^8^ The Connor-Davidson Resilience Scale (CD-RISC) is a questionnaire consisting of 25 items that map broadly onto five factors: reliable measure of resilience consisting of 25 items that broadly map onto five factors: (1) personal competence, high standards and tenacity; (2) trust in one's instincts, tolerance of negative affect, and strengthening effects of stress; (3) positive acceptance of change, and secure relationships; (4) control and (5) spiritual influences.^9^

*Hormonal Status and use of Contraceptives*

As hormonal status can also play a role in abdominal symptoms, psychological state, and pain perception ^10^, use of hormonal contraceptives was also recorded (N = 15). 55 women were premenopausal, 1 woman was perimenopausal, and 4 women were postmenopausal.

| Hormonal Contraceptive | Dosage | N |
| --- | --- | --- |
| Mirena IUD | IUD | 2 |
| Kyleena IUD | IUD | 1 |
| Implanon (etonogestrel implant) | Implant | 1 |
| Microgestin (oral) | Once a day | 2 |
| Nuvaring (etonogestrel/ethinyl estradiol vaginal ring) | Inserted for 3 weeks, then 1 week break | 2 |
| Yaz (oral) | Once a day | 1 |
| Provera (oral) | Shot every 3 months | 1 |
| Estarylla | Once a day | 1 |
| Undisclosed - Oral Pill | Once a day | 4 |
| Total |  | 15 |

**Descriptive Statistics**

**Supplementary Table 1**

|  | **Improver (N=24)** | **Non-Improver (N=36)** | **p-value** | **Cohen’s D** |
| --- | --- | --- | --- | --- |
| **SOCIODEMOGRAPHICS** |  |  |  |  |
| **Age** |  |  |  |  |
| Mean (SD) | 25.9 (8.71) | 31.2 (12.7) | 0.061 | 0.469 |
| Median [Min, Max] | 23.5 [18.0, 48.0] | 27.0 [18.0, 60.0] |  |  |
| **BMI** |  |  |  |  |
| Mean (SD) | 23.6 (3.37) | 24.1 (3.91) | 0.592 | 0.138 |
| Median [Min, Max] | 23.4 [18.8, 31.5] | 23.1 [17.4, 34.5] |  |  |
| **Income** |  |  |  |  |
| Mean (SD) | 6.39 (2.74) | 6.21 (2.76) | 0.804 | 0.067 |
| Median [Min, Max] | 7.00 [1.00, 9.00] | 7.00 [1.00, 9.00] |  |  |
| Missing | 1 (4.2%) | 2 (5.6%) |  |  |
| **SYMPTOMOLOGY** |  |  |  |  |
| **Baseline IBS-SSS Score** |  |  |  |  |
| Mean (SD) | 250 (70.0) | 256 (63.5) | 0.784 | 0.084 |
| Median [Min, Max] | 234 [156, 381] | 253 [155, 455] |  |  |
| Missing | 6 (25.0%) | 4 (11.1%) |  |  |
| **3 Month IBS-SSS Change Score** |  |  |  |  |
| Mean (SD) | -85.2 (31.8) | 33.9 (70.0) | <0.001 | 2.055 |
| Median [Min, Max] | -74.5 [-171, -53.0] | 17.0 [-49.0, 223] |  |  |
| **Baseline BSQ Overall Symptoms Score** |  |  |  |  |
| Mean (SD) | 11.6 (3.34) | 9.76 (3.99) | 0.071 | 0.481 |
| Median [Min, Max] | 11.0 [6.00, 16.0] | 10.5 [0, 16.0] |  |  |
| Missing | 1 (4.2%) | 2 (5.6%) |  |  |
| **Baseline BSQ Abdominal Pain Score** |  |  |  |  |
| Mean (SD) | 10.0 (4.56) | 9.03 (3.99) | 0.412 | 0.230 |
| Median [Min, Max] | 10.0 [2.00, 17.0] | 10.0 [0, 16.0] |  |  |
| Missing | 1 (4.2%) | 2 (5.6%) |  |  |
| **PSYCHOSOCIAL QUESTIONNAIRES** |  |  |  |  |
| **Baseline HADS Anxiety** |  |  |  |  |
| Mean (SD) | 8.33 (4.32) | 6.92 (3.48) | 0.187 | 0.369 |
| Median [Min, Max] | 7.00 [3.00, 17.0] | 7.00 [0, 15.0] |  |  |
| **Baseline HADS Depression** |  |  |  |  |
| Mean (SD) | 3.08 (3.02) | 2.53 (2.99) | 0.487 | 0.185 |
| Median [Min, Max] | 2.50 [0, 9.00] | 2.00 [0, 11.0] |  |  |
| **Baseline CDRISC Persistence Score** |  |  |  |  |
| Mean (SD) | 25.0 (5.83) | 25.4 (5.19) | 0.759 | 0.083 |
| Median [Min, Max] | 25.5 [9.00, 32.0] | 26.5 [9.00, 32.0] |  |  |
| **Baseline CDRISC Emotional Cognitive Score** |  |  |  |  |
| Mean (SD) | 19.5 (5.00) | 19.7 (3.37) | 0.862 | 0.050 |
| Median [Min, Max] | 20.5 [7.00, 28.0] | 20.0 [13.0, 25.0] |  |  |
| **Baseline CDRISC Adaptability Score** |  |  |  |  |
| Mean (SD) | 16.8 (2.83) | 16.3 (3.08) | 0.5 | 0.176 |
| Median [Min, Max] | 17.5 [10.0, 20.0] | 16.0 [8.00, 20.0] |  |  |
| **Baseline CDRISC Control/Meaning Score** |  |  |  |  |
| Mean (SD) | 9.74 (2.38) | 8.83 (2.73) | 0.184 | 0.348 |
| Median [Min, Max] | 10.0 [3.00, 12.0] | 9.50 [1.00, 12.0] |  |  |
| Missing | 1 (4.2%) | 0 (0%) |  |  |
| **Baseline CDRISC Meaning Score** |  |  |  |  |
| Mean (SD) | 4.73 (2.21) | 4.59 (2.54) | 0.829 | 0.058 |
| Median [Min, Max] | 5.50 [0, 8.00] | 5.00 [0, 8.00] |  |  |
| Missing | 2 (8.3%) | 2 (5.6%) |  |  |
| **Baseline CDRISC Total Score** |  |  |  |  |
| Mean (SD) | 75.2 (15.4) | 74.7 (13.0) | 0.904 | 0.033 |
| Median [Min, Max] | 78.5 [39.0, 100] | 78.0 [35.0, 95.0] |  |  |
| **Baseline Trait Anxiety** |  |  |  |  |
| Mean (SD) | 50.2 (12.7) | 50.2 (10.2) | 0.993 | 0.002 |
| Median [Min, Max] | 46.0 [35.0, 72.0] | 51.0 [34.0, 77.0] |  |  |
| **Baseline Perceived Stress Score** |  |  |  |  |
| Mean (SD) | 16.7 (6.84) | 15.1 (6.49) | 0.383 | 0.235 |
| Median [Min, Max] | 16.5 [5.00, 30.0] | 14.0 [4.00, 34.0] |  |  |
| **Baseline IPIP Neuroticism** |  |  |  |  |
| Mean (SD) | 24.1 (7.87) | 23.7 (7.01) | 0.835 | 0.057 |
| Median [Min, Max] | 22.0 [10.0, 41.1] | 23.0 [10.0, 38.0] |  |  |
| **Baseline IPIP Extraversion** |  |  |  |  |
| Mean (SD) | 36.4 (8.75) | 34.7 (7.44) | 0.429 | 0.217 |
| Median [Min, Max] | 36.0 [13.3, 48.0] | 35.0 [17.0, 46.0] |  |  |
| **Baseline CMSI 12 Month Score** |  |  |  |  |
| Mean (SD) | 6.26 (6.58) | 7.83 (6.43) | 0.371 | 0.242 |
| Median [Min, Max] | 5.00 [1.00, 27.0] | 5.00 [0, 23.0] |  |  |
| Missing | 1 (4.2%) | 0 (0%) |  |  |
| **Baseline CMSI Lifetime Score** |  |  |  |  |
| Mean (SD) | 7.78 (7.69) | 9.64 (7.87) | 0.375 | 0.238 |
| Median [Min, Max] | 5.00 [1.00, 31.0] | 8.50 [0, 32.0] |  |  |
| Missing | 1 (4.2%) | 0 (0%) |  |  |
| **Baseline ETI General Score** |  |  |  |  |
| Mean (SD) | 1.25 (1.26) | 2.00 (1.77) | 0.06 | 0.471 |
| Median [Min, Max] | 1.00 [0, 4.00] | 2.00 [0, 6.00] |  |  |
| **Baseline ETI Physical Score** |  |  |  |  |
| Mean (SD) | 1.25 (1.62) | 0.944 (1.31) | 0.445 | 0.212 |
| Median [Min, Max] | 0 [0, 5.00] | 0 [0, 5.00] |  |  |
| **Baseline ETI Emotional Score** |  |  |  |  |
| Mean (SD) | 1.33 (1.63) | 1.19 (1.35) | 0.731 | 0.095 |
| Median [Min, Max] | 0.500 [0, 5.00] | 1.00 [0, 5.00] |  |  |
| **Baseline ETI Sexual Score** |  |  |  |  |
| Mean (SD) | 0.333 (0.917) | 0.861 (1.22) | 0.061 | 0.475 |
| Median [Min, Max] | 0 [0, 4.00] | 0 [0, 5.00] |  |  |
| **Baseline ETI Total Score** |  |  |  |  |
| Mean (SD) | 4.17 (3.50) | 5.00 (4.11) | 0.403 | 0.215 |
| Median [Min, Max] | 3.50 [0, 12.0] | 4.00 [0, 15.0] |  |  |
| **QUANTITATIVE SENSORY TESTING** |  |  |  |  |
| **Baseline Pain Threshold** |  |  |  |  |
| Mean (SD) | 41.4 (4.28) | 43.7 (4.11) | 0.044 | 0.551 |
| Median [Min, Max] | 42.9 [33.5, 47.8] | 44.8 [34.9, 49.0] |  |  |
| **Baseline Pain Tolerance** |  |  |  |  |
| Mean (SD) | 46.9 (2.98) | 47.9 (3.66) | 0.25 | 0.294 |
| Median [Min, Max] | 46.9 [36.2, 50.0] | 48.9 [33.3, 51.0] |  |  |
| **Baseline Pain Intensity Rating** |  |  |  |  |
| Mean (SD) | 9.88 (3.55) | 10.8 (4.35) | 0.375 | 0.226 |
| Median [Min, Max] | 10.0 [2.00, 15.0] | 12.0 [1.00, 17.0] |  |  |
| **Baseline Pain Threshold Unpleasantness Rating** |  |  |  |  |
| Mean (SD) | 6.44 (4.32) | 7.50 (3.86) | 0.336 | 0.262 |
| Median [Min, Max] | 5.50 [0, 14.0] | 7.00 [1.00, 16.0] |  |  |
| **Baseline Pain Tolerance Unpleasantness Rating** |  |  |  |  |
| Mean (SD) | 11.6 (4.10) | 12.3 (3.80) | 0.516 | 0.175 |
| Median [Min, Max] | 13.0 [2.00, 16.0] | 12.3 [1.00, 19.0] |  |  |

Table showing descriptive comparisons between improvers and non-improvers after 3 months of behavioral/clinical variables used in the DIABLO analysis.

Abbreviations: BMI – body mass index; IBS-SS – IBS symptom severity; BSQ – Bowel Symptoms Questionnaire; HADS – Hospital Anxiety and Depression Scale; CDRISC – Connor-Davidson Resilience Scale; IPIP – International Personality Item Pool; CMSI – Complex Multi Symptom Inventory; ETI – Early Trauma Inventory; SD – standard deviation

**Supplementary Table 2**

|  | **Improver (N=17)** | **Non-Improver (N=26)** | **p-value** | **Cohen’s D** |
| --- | --- | --- | --- | --- |
| **SOCIODEMOGRAPHICS** |  |  |  |  |
| **Age** |  |  |  |  |
| Mean (SD) | 26.6 (10.9) | 30.2 (12.6) | 0.32 | 0.304 |
| Median [Min, Max] | 23.0 [18.0, 55.0] | 25.5 [19.0, 60.0] |  |  |
| **BMI** |  |  |  |  |
| Mean (SD) | 24.9 (3.09) | 23.8 (3.75) | 0.326 | 0.298 |
| Median [Min, Max] | 25.5 [20.6, 30.4] | 23.9 [17.4, 32.4] |  |  |
| **Income** |  |  |  |  |
| Mean (SD) | 7.13 (2.73) | 5.92 (2.74) | 0.178 | 0.441 |
| Median [Min, Max] | 9.00 [1.00, 9.00] | 6.00 [1.00, 9.00] |  |  |
| Missing | 1 (5.9%) | 1 (3.8%) |  |  |
| **SYMPTOMOLOGY** |  |  |  |  |
| **Baseline IBS-SSS Score** |  |  |  |  |
| Mean (SD) | 272 (70.5) | 235 (48.1) | 0.109 | 0.649 |
| Median [Min, Max] | 268 [167, 373] | 239 [156, 315] |  |  |
| Missing | 4 (23.5%) | 5 (19.2%) |  |  |
| **12 Month IBS-SSS Change Score** |  |  |  |  |
| Mean (SD) | -120 (45.6) | 45.3 (56.1) | <0.001 | 3.159 |
| Median [Min, Max] | -115 [-207, -51.0] | 42.0 [-47.0, 189] |  |  |
| **Baseline BSQ Overall Symptoms Score** |  |  |  |  |
| Mean (SD) | 12.5 (3.22) | 9.00 (3.99) | 0.004 | 0.937 |
| Median [Min, Max] | 13.0 [4.00, 16.0] | 9.00 [0, 16.0] |  |  |
| Missing | 0 (0%) | 1 (3.8%) |  |  |
| **Baseline BSQ Abdominal Pain Score** |  |  |  |  |
| Mean (SD) | 11.0 (3.91) | 7.68 (3.79) | 0.01 | 0.865 |
| Median [Min, Max] | 12.0 [4.00, 16.0] | 8.00 [0, 15.0] |  |  |
| Missing | 0 (0%) | 1 (3.8%) |  |  |
| **PSYCHOSOCIAL QUESTIONNAIRES** |  |  |  |  |
| **Baseline HADS Anxiety** |  |  |  |  |
| Mean (SD) | 7.94 (3.44) | 7.08 (3.07) | 0.407 | 0.269 |
| Median [Min, Max] | 7.00 [3.00, 15.0] | 7.00 [2.00, 15.0] |  |  |
| **Baseline HADS Depression** |  |  |  |  |
| Mean (SD) | 3.12 (3.06) | 1.81 (2.28) | 0.142 | 0.501 |
| Median [Min, Max] | 3.00 [0, 9.00] | 1.00 [0, 11.0] |  |  |
| **Baseline CDRISC Persistence Score** |  |  |  |  |
| Mean (SD) | 25.9 (4.51) | 26.6 (3.49) | 0.609 | 0.170 |
| Median [Min, Max] | 26.0 [18.1, 32.0] | 27.0 [17.0, 32.0] |  |  |
| **Baseline CDRISC Emotional Cognitive Score** |  |  |  |  |
| Mean (SD) | 20.1 (5.34) | 20.3 (2.94) | 0.841 | 0.071 |
| Median [Min, Max] | 22.0 [7.00, 28.0] | 20.5 [15.0, 25.0] |  |  |
| **Baseline CDRISC Adaptability Score** |  |  |  |  |
| Mean (SD) | 16.6 (3.12) | 16.8 (2.69) | 0.831 | 0.069 |
| Median [Min, Max] | 18.0 [10.0, 20.0] | 17.0 [10.0, 20.0] |  |  |
| **Baseline CDRISC Control/Meaning Score** |  |  |  |  |
| Mean (SD) | 9.81 (2.37) | 9.62 (2.06) | 0.786 | 0.090 |
| Median [Min, Max] | 10.0 [5.00, 12.0] | 10.0 [3.00, 12.0] |  |  |
| Missing | 1 (5.9%) | 0 (0%) |  |  |
| **Baseline CDRISC Meaning Score** |  |  |  |  |
| Mean (SD) | 5.00 (2.52) | 4.42 (2.10) | 0.441 | 0.255 |
| Median [Min, Max] | 6.00 [0, 8.00] | 5.00 [1.00, 8.00] |  |  |
| Missing | 0 (0%) | 2 (7.7%) |  |  |
| **Baseline CDRISC Total Score** |  |  |  |  |
| Mean (SD) | 77.0 (14.8) | 77.9 (8.95) | 0.826 | 0.077 |
| Median [Min, Max] | 78.0 [43.6, 100] | 79.0 [57.0, 90.5] |  |  |
| **Baseline Trait Anxiety** |  |  |  |  |
| Mean (SD) | 49.9 (12.0) | 48.5 (9.99) | 0.688 | 0.131 |
| Median [Min, Max] | 46.0 [35.0, 71.0] | 46.0 [35.0, 77.0] |  |  |
| **Baseline Perceived Stress Score** |  |  |  |  |
| Mean (SD) | 15.5 (6.47) | 15.5 (5.57) | 0.972 | 0.114 |
| Median [Min, Max] | 14.0 [5.00, 30.0] | 15.0 [6.00, 34.0] |  |  |
| **Baseline Neuroticism** |  |  |  |  |
| Mean (SD) | 24.9 (8.34) | 22.2 (5.10) | 0.228 | 0.426 |
| Median [Min, Max] | 23.0 [10.0, 41.1] | 21.5 [13.0, 33.0] |  |  |
| **Baseline IPIP Extraversion** |  |  |  |  |
| Mean (SD) | 35.6 (8.69) | 34.9 (8.41) | 0.8 | 0.080 |
| Median [Min, Max] | 36.0 [13.3, 48.0] | 36.0 [17.0, 48.0] |  |  |
| **Baseline CMSI 12 Month Score** |  |  |  |  |
| Mean (SD) | 5.47 (4.03) | 7.80 (6.81) | 0.172 | 0.397 |
| Median [Min, Max] | 5.00 [1.00, 13.0] | 5.00 [1.00, 23.0] |  |  |
| Missing | 0 (0%) | 1 (3.8%) |  |  |
| **Baseline CMSI Lifetime Score** |  |  |  |  |
| Mean (SD) | 7.24 (5.03) | 9.68 (8.71) | 0.257 | 0.328 |
| Median [Min, Max] | 8.00 [1.00, 15.0] | 7.00 [1.00, 32.0] |  |  |
| Missing | 0 (0%) | 1 (3.8%) |  |  |
| **Baseline ETI General Score** |  |  |  |  |
| Mean (SD) | 1.59 (1.42) | 1.50 (1.68) | 0.854 | 0.056 |
| Median [Min, Max] | 1.00 [0, 4.00] | 1.00 [0, 5.00] |  |  |
| **Baseline ETI Physical Score** |  |  |  |  |
| Mean (SD) | 0.706 (1.21) | 1.15 (1.57) | 0.299 | 0.311 |
| Median [Min, Max] | 0 [0, 4.00] | 0 [0, 5.00] |  |  |
| **Baseline ETI Emotional Score** |  |  |  |  |
| Mean (SD) | 0.882 (1.45) | 1.08 (1.32) | 0.66 | 0.141 |
| Median [Min, Max] | 0 [0, 5.00] | 1.00 [0, 5.00] |  |  |
| **Baseline ETI Sexual Score** |  |  |  |  |
| Mean (SD) | 0.353 (0.702) | 0.769 (1.24) | 0.169 | 0.391 |
| Median [Min, Max] | 0 [0, 2.00] | 0 [0, 4.00] |  |  |
| **Baseline ETI Total Score** |  |  |  |  |
| Mean (SD) | 3.53 (3.20) | 4.50 (4.10) | 0.391 | 0.257 |
| Median [Min, Max] | 2.00 [0, 9.00] | 3.50 [0, 15.0] |  |  |
| **QUANTITATIVE SENSORY TESTING** |  |  |  |  |
| **Baseline Pain Threshold** |  |  |  |  |
| Mean (SD) | 42.8 (4.53) | 43.2 (4.00) | 0.785 | 0.088 |
| Median [Min, Max] | 44.0 [35.2, 48.0] | 44.0 [33.5, 48.6] |  |  |
| **Baseline Pain Tolerance** |  |  |  |  |
| Mean (SD) | 48.3 (2.09) | 47.7 (3.00) | 0.415 | 0.238 |
| Median [Min, Max] | 48.9 [44.7, 51.0] | 48.4 [38.1, 51.0] |  |  |
| **Baseline Pain Intensity Rating** |  |  |  |  |
| Mean (SD) | 9.71 (4.25) | 10.4 (4.36) | 0.616 | 0.157 |
| Median [Min, Max] | 10.0 [1.00, 15.0] | 11.0 [2.00, 17.0] |  |  |
| **Baseline Pain Threshold Unpleasantness Rating** |  |  |  |  |
| Mean (SD) | 6.65 (3.98) | 7.65 (4.50) | 0.446 | 0.234 |
| Median [Min, Max] | 7.00 [0, 14.0] | 7.00 [0, 15.0] |  |  |
| **Baseline Pain Tolerance Unpleasantness Rating** |  |  |  |  |
| Mean (SD) | 12.0 (3.87) | 12.2 (4.36) | 0.857 | 0.055 |
| Median [Min, Max] | 13.0 [7.00, 17.0] | 13.3 [1.00, 19.0] |  |  |
|  |  |  |  |  |

Table showing descriptive comparisons between improvers and non-improvers after 12 months of behavioral/clinical variables used in the DIABLO analysis.

Abbreviations: BMI – body mass index; IBS-SS – IBS symptom severity; BSQ – Bowel Symptoms Questionnaire; HADS – Hospital Anxiety and Depression Scale; CDRISC – Connor-Davidson Resilience Scale; IPIP – International Personality Item Pool; CMSI – Complex Multi Symptom Inventory; ETI – Early Trauma Inventory; SD – standard deviation

*Neuroimaging Acquisition*

All neuroimaging was conducted at baseline at the Ahmanson-Lovelace Brain Mapping Center on a 3.0 Tesla Siemens Prisma MRI Scanner and a 20-channel head coil. T1 weighted MP-RAGE scans acquired to assess brain structure (TR: 2300ms, TE: 2.98ms, TI: 900ms, flip angle: 9˚, field of view: 240 x 256 mm, acquisition matrix: 240 x 256, slice thickness: 1 mm, voxel resolution: 1 x 1 x 1 mm). A 10-minute resting-state fMRI scan was acquired to assess resting-state functional connectivity (TR: 2000ms, TE: 28ms, flip angle: 77˚, acquisition matrix: 64 x 64, slice thickness: 4 mm, voxel resolution: 3.44 x 3.44 x 4 mm, 300 volumes). A diffusion weighted image was acquired to assess white matter anatomical connectivity (64 noncollinear directions, b = 1000 s/mm^2^, 9 b = 0 s/mm^2^ images, TR: 9500ms, TE: 88ms, field of view: 2304 x 2304, acquisition matrix: 128 x 128, slice thickness: 2mm, spacing between slices: 2mm).

*Structural Image Processing*

Cortical reconstruction and volumetric segmentation was done using the FreeSurfer 6 analysis suite ^11^. All participants’ T1 structural data was first parcellated using the Human Connectome Project Multimodal cortical atlas ^12^ and the Harvard-Oxford subcortical ^13–16^ atlas. FreeSurfer then computed values of cortical thickness, surface area, mean curvature and volume for cortical ROIs and volume for subcortical ROIs (**Table 2)**.

*Functional Image Processing*

All functional data was run through a preprocessing pipeline for surface-based resting-state functional connectivity (rs-FC) analyses in CONN ^17^. All functional data went through realignment and unwarping, slice-timing correction, outlier identification (ART-based identification of outlier scans for scrubbing), indirect co-registration to structural data using a non-linear transformation ^18,19^, resampling of functional data to the location of the subject-specific structural cortical surface, and functional surface space smoothing using iterative diffusion smoothing with 40 iterations, approximately a 8mm FWHM kernel ^20^. Data was then denoised by using ordinary least squares (OLS) regression of potential confounding effects and temporal band-pass filtering. The default anatomical component-based noise correction procedure (aCompCor) include noise components from white-matter, cerebrospinal fluid ^21^, estimated subject-motion parameters ^22^, outlier scans or scrubbing based on framewise displacement ^23^, and effect of rest representing potential ramping effects at the start of the session ^24^. A temporal band-pass filter between 0.008 – 0.09 Hz after regression was used to minimize the influence of physiological, head-motion and other noise sources ^25^. Fisher transformed correlations were computed (Z) in CONN between the functional time series of all of the parcellated regions done in FreeSurfer to derive a 377 x 377 matrix for each participant. The bottom half of the undirected matrix was then concatenated into one vector for each subject representing every ROI pair.

*Diffusion Processing*

All diffusion weighted images were first corrected for eddy current-induced distortions and movement using FSL’s *eddy_correct* tool ^26^. Images along with b-vectors and b-values were then converted from FSL to Camino data formats using *fsl2scheme* and *image2voxel* in Camino ^27^. A diffusion tensor was then fit on the voxel order data in Camino (*wdtfit)* using weighted linear least squares regression ^27^. Deterministic and probabilistic tensor based approaches have been shown to perform similarly ^28^. Whole-brain deterministic tractography was then performed using the *track* command in Camino euler algorithm with a step size of 0.5 and curve threshold of 76. Connectivity matrices were the constructed using the *conmat* command in Camino, which computes a matrix counting the number of streamlines connecting each pair of ROIs computed in the aforementioned FreeSurfer and CONN analyses. This resulted in a 377 x 377 matrix for each participant. Every subject’s matrix went through within-subject normalization by taking a sum of all counts between each ROI, then dividing each pair’s count by that total. The bottom half of the undirected matrix was then concatenated into one vector for each subject representing every ROI pair.

*Neuroimaging data preparation for DIABLO*

In the morphometry datasets, all data was residualized by the estimated total intercranial volume (eTIV) to control for effects driven by brain size ^29,30^. As the nature of the diffusion dataset can consist of many variables with zeros (i.e., regions that do not share anatomic connections), near zero variance predictors were removed (with the cutoff for the ratio of the most common value to the second most common value being 95/5 and the cutoff for the percentage of distinct values out of the number of samples being 50%), reducing the dataset from 70880 features to 2966. The final morphometry dataset had 1466 features, anatomical connectivity dataset had 2966 features, resting-state connectivity dataset had 70876 features, and clinical dataset had 26 features (**Table 1**). All four datasets (including clinical data) were then scaled and centered separately by calculating mean and SD of each vector, then “scaling” each element by subtracting the mean and dividing by the SD. These datasets were entered into subsequent analyses.

*Training and Testing Sets*

In the analysis looking at 3-month symptom changes, 60 women (mean age = 29.08, SD = 11.46) had data from baseline to 3 months due to attrition from baseline. Data were split for training and test sets using an 80/20% split with the same proportion of improvers and non-improvers going in both datasets (training: N = 49 (29 non-improvers), testing: N = 11 (7 non-improvers)).

In the analysis looking at 12-month symptom changes, 43 women (mean age = 28.79, SD = 11.96) had data from baseline to 12 months due to attrition from baseline. Data were split for training and test sets using an 70/30 % split (due to the smaller sample size) with the same proportion of improvers and non-improvers going in both datasets (training: N = 31 (19 non-improvers), testing: N = 12 (7 non-improvers)). An independent t-test was done to confirm that age was similar in train (M = 27.00, SD = 10.05) and test (M = 33.42, SD = 15.45) datasets (*t* = -1.33, *p* = 0.20, *d* = 0.55). There was no significant difference in age between improvers and non-improvers in the training dataset (*t* = 0.97, *p* = 0.34, *d* = 0.38).

*DIABLO Analysis*

To determine the design matrix for the DIABLO analysis, a data-driven approach was used. Since the correlation between the first component of each dataset was greater than 0.8, all the weights in the 3-month and 12-month design matrices were set to 1.^31,32^ (Fig. S1). Once the design matrix was determined, A DIABLO model with 5 components was first fit without any variable selection, and global performance was assessed using leave-one-out-cross validation (LOOCV). The number of components chosen based on the lowest balanced error rate (BER) and distance metric (maximum distance vs centroids distance vs mahalanobis distance) across number of components. After determining the number of components to use, the optimal number of variables to be kept per component, one component at a time, by defining a grid of values – in this case from 2 to 300 - for each component. LOOCV was run with the distance metric defined above to give the lowest BER. The classification error rate was then extracted averaged across every LOOCV model for each tested grid value. The optimal number of components, and features per component was then extracted. Since this process may lead to overfitting, manual tuning of number of features per component was then conducted to get the lowest balanced error rate and best accuracy on a holdout testing dataset. The main output measures for DIABLO are a set of components (i.e. latent variables) chosen in the model, a set of loading vectors (i.e. coefficients assigned to each variable to define each component), and a list of selected variables from each dataset and associated to each component. Loadings are the coefficients assigned to each variable to define each component, and their absolute value represent the importance of each variable in DIABLO. It is important to note that each loading vector is assigned to a particular component, and the loading vectors are obtained so that the covariance between a linear combination of X variables and Y is maximized. Individual sample plots represent each individual projected onto a space that is defined by the components. The coordinates for each individual is determined by their component values/scores. Loading plots help visualize each coefficient (i.e. importance) assigned to the variables in each component of each dataset. Circos diagrams are built on a similarity matrix ^33^ and represent the correlation between variables from different datasets, and a cutoff was chosen as r = 0.7 as this is universally considered a “strong” correlation. Relevance networks are graphs where the nodes represent the variables chosen by DIABLO and the edges represent variable associations. Edges between nodes were only drawn of the association was 0.7 or higher. Nodes/variables extracted from DIABLO that did not have any associations were not represented on the network. The area under the receiver operating characteristic (ROC) curve was calculated for each dataset separately by component, and p-values were calculated using the Wilcoxon test comparing improvers vs non-improvers. The area under the ROC curve is a way to summarize the overall diagnostic accuracy of the model. The values range from 0 to 1, where 1 represents a perfectly accurate model. A value of 0.5 represents no discriminatory ability. Values between 0.7-0.8 are considered acceptable, while between 0.8-0.9 is considered excellent, and greater values are considered outstanding ^34^.

**
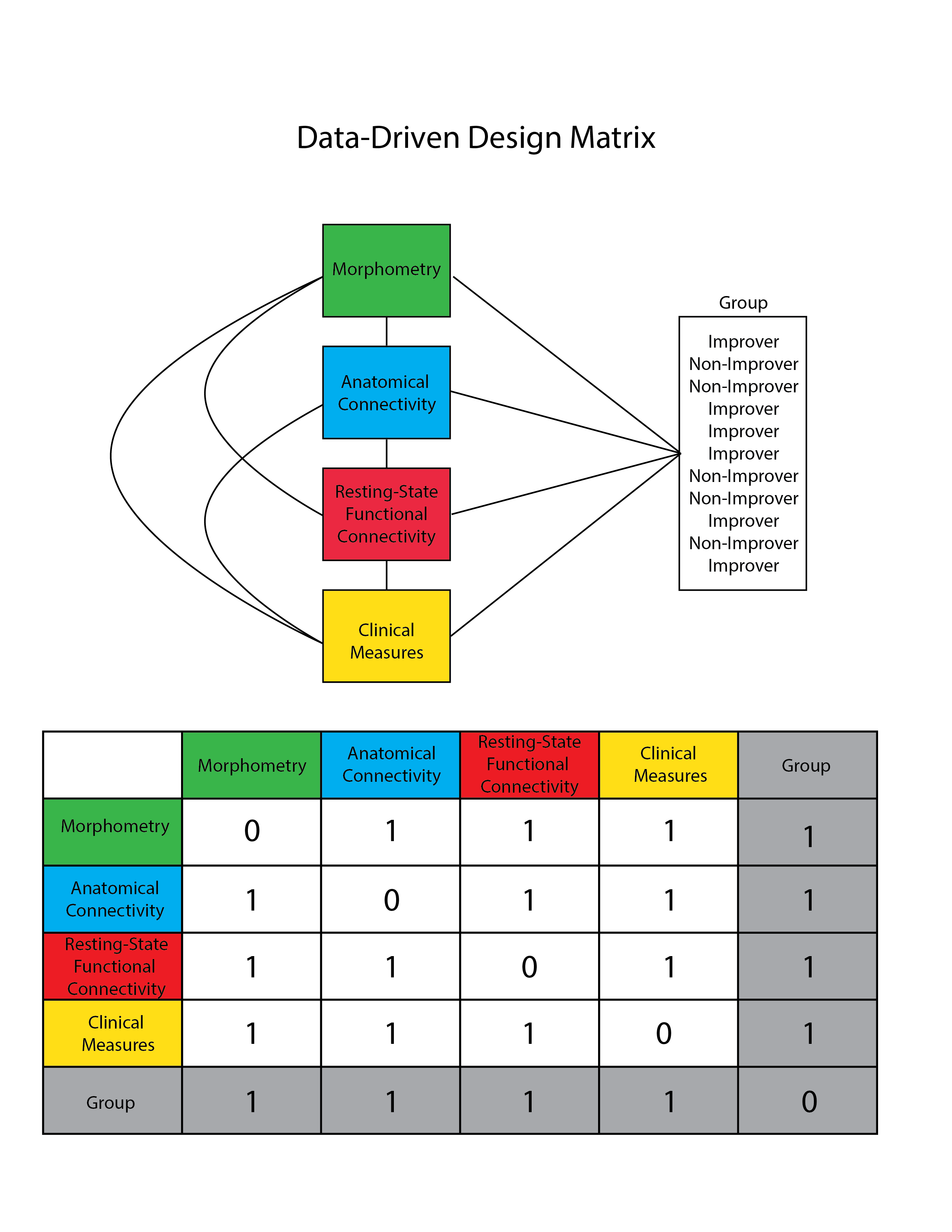
Supplementary Figure 1**

Design matrix for DIABLO analysis for classifying 3 month and 12 month symptom improvers and non-improvers on the training set. Weights between datasets represent the connectedness between datasets. Values are determined by taking the correlation between the first component from each dataset’s sPLS analysis. All values were above r = 0.8, so the values were set to 1. Lines represent which datasets are connected together which together are trying to predict which participants are improvers vs. non-improvers.

*sPLS on between datasets for 3- and 12-month analyses*

In order to understand what features would be useful in classifying improvers and non-improvers at both 3 and 12 months, as well as to guide data integration, a sPLS analysis^35^ was completed with each dataset in a pairwise manner (i.e. morphometry vs. anatomical connectivity, morphometry vs. resting-state functional connectivity, anatomical connectivity vs. resting-state functional connectivity, clinical vs. morphometry, clinical vs. anatomical connectivity, clinical vs. resting-state functional connectivity). A canonical mode was used in the sPLS to model a bi-directional relationship between the two datasets.^36^ The output from the sPLS includes a set of components (i.e. latent variables), a set of loading vectors, and a list of selected variables. Clustered image maps, arrow plots, and loadings plots are shown to ease the interpretation between the two types of variables from each dataset. Clustered image maps show the correlation structure between the types of variables. Arrow plots project the samples onto a space which is an overlap of the X and Y (i.e. both datasets) representation plots. Each arrow joins the individual sample from X space to Y space. A short arrow represents a good agreement with both datasets via sPLS. Loading plots help visualize each coefficient (i.e. importance) assigned to the variables in each component of each dataset. Loadings are the coefficients assigned to each variable to define each component, and their absolute value represent the importance of each variable in the sPLS. It is important to note that each loading vector is assigned to a particular component, and the loading vectors are obtained so that the covariance between a linear combination of X variables and Y is maximized.

The design matrices for data integration using DIABLO were determined via this sPLS approach by modelling pairwise associations. The correlation between the first components between both datasets in each model were computed. As all correlations were above r = 0.8, then the connection between the datasets was set to 1 in the design matrix for both DIABLO models (**Supplementary Figure 7**).^32^

**Supplementary sPLS Results**

*sPLS between morphometry and anatomical connectivity data for 3-month improvers and non-improvers*

A two component sPLS model was used with 50 features from each dataset on each component. The correlation between the first components of the two datasets was *r_(47)_* = 0.94,  *p* < 2.2 x 10^-16^, and thus the weight of the design matrix in the DIABLO model classifying improvers vs non-improvers was set to 1. Plots to guide data integration are shown below in **Fig S2**.


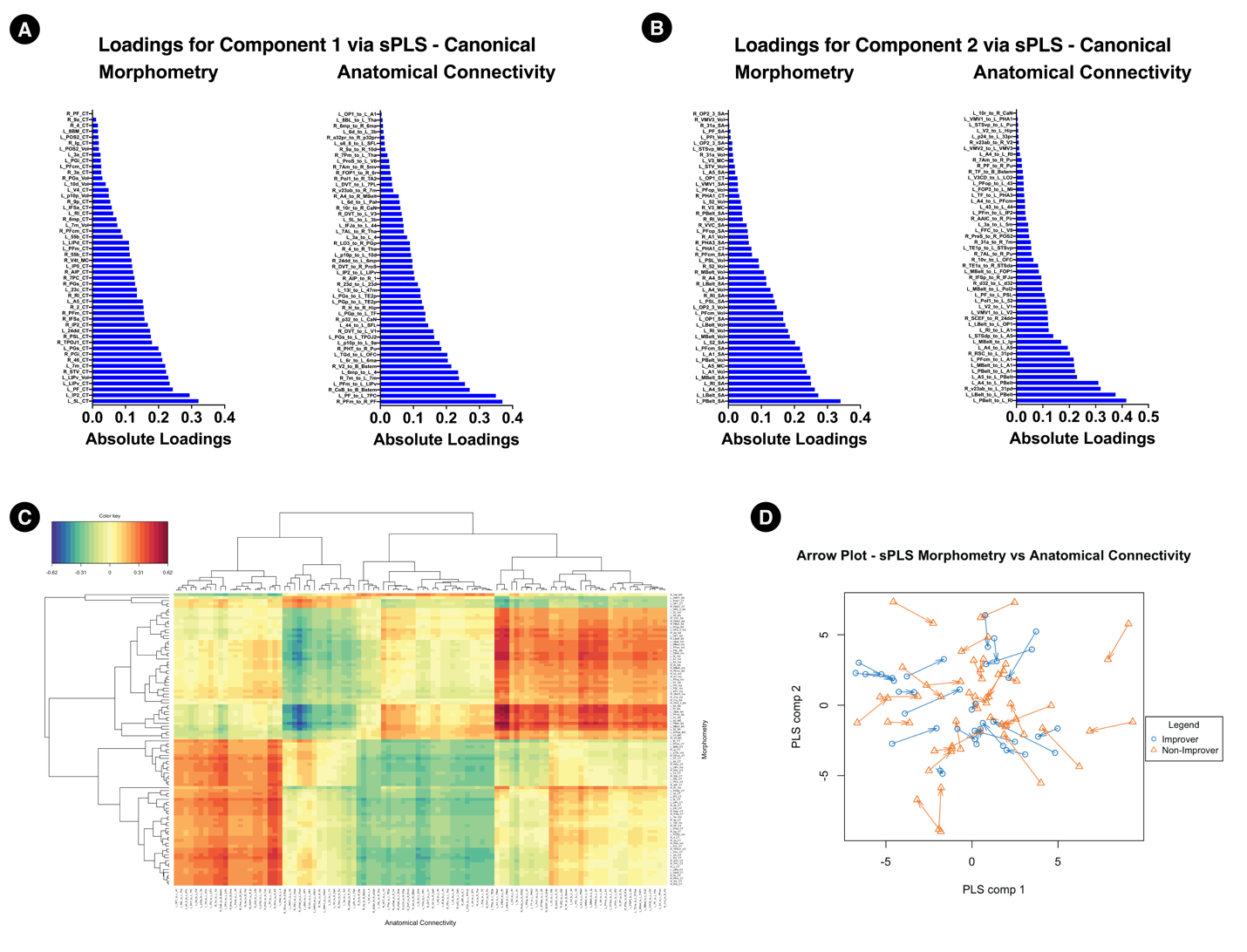


Fig S2: (A) Loadings plots for component 1 per data type, (B) Loadings plots for component 2 per data type, (C) Clustered image map showing the correlation structure between morphometry and anatomical connectivity, (D) Arrow plot showing agreeableness between datasets for each sample. Reference Supplementary Table 1 for regions of interest. sPLS shows strong correlation between datasets.

Abbreviations: Vol – volume, CT – cortical thickness, SA – surface area, MC – mean curvature

*sPLS between morphometry and resting-state functional connectivity data for 3-month improvers and non-improvers*

A two component sPLS model was used with 50 features from each dataset on each component. The correlation between the first components of the two datasets was *r_(47)_* = 0.93,  *p* < 2.2 x 10^-16^, and thus the weight of the design matrix in the DIABLO model classifying improvers vs non-improvers was set to 1. Plots to guide data integration are shown below in **Fig S3**.


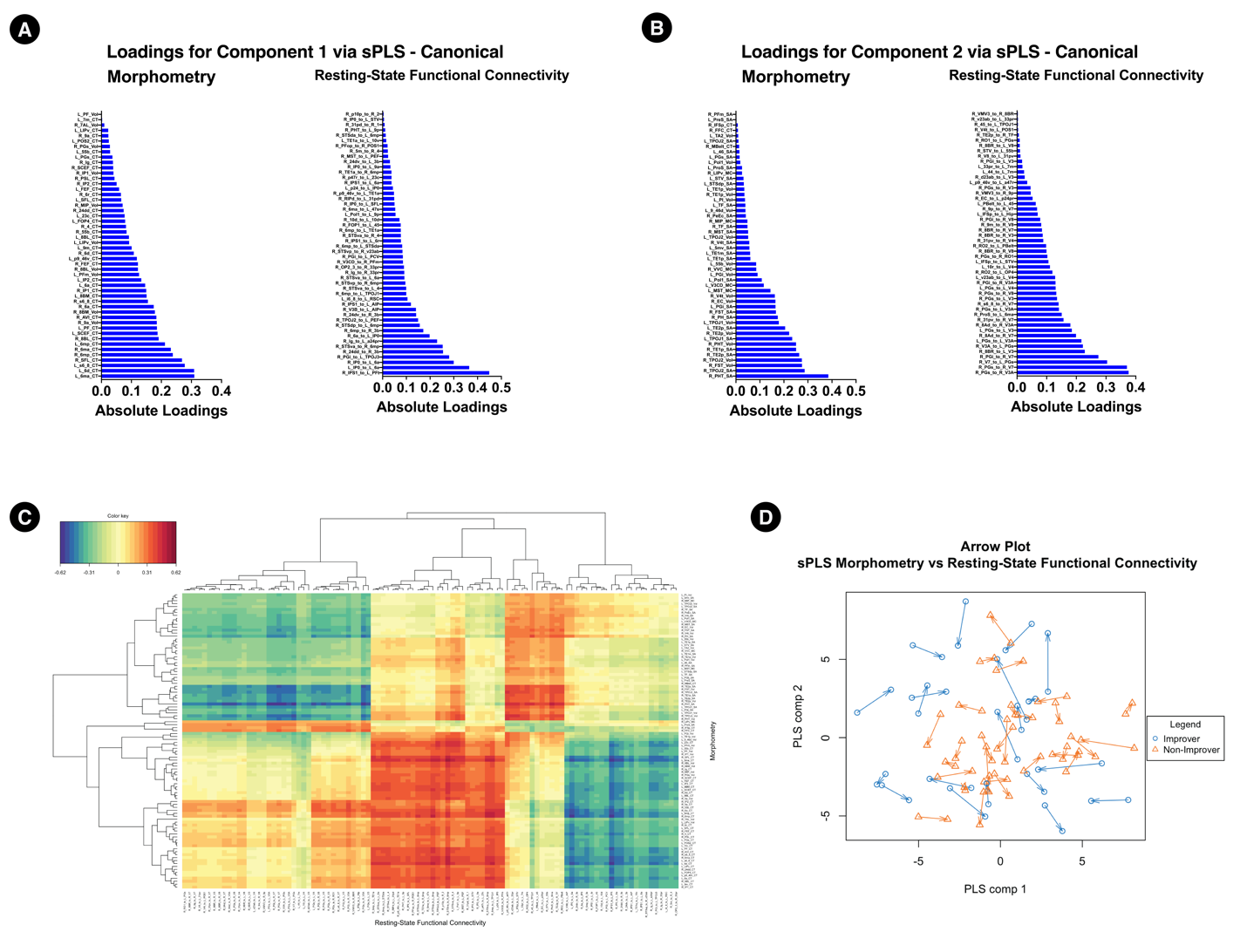


Fig S3: (A) Loadings plots for component 1 per data type, (B) Loadings plots for component 2 per data type, (C) Clustered image map showing the correlation structure between morphometry and resting-state functional connectivity, (D) Arrow plot showing agreeableness between datasets for each sample. Reference Supplementary Table 1 for regions of interest. sPLS shows strong correlation between datasets.

Abbreviations: Vol – volume, CT – cortical thickness, SA – surface area, MC – mean curvature

*sPLS between anatomical connectivity and resting-state functional connectivity data for 3-month improvers and non-improvers*

A two component sPLS model was used with 50 features from each dataset on each component. The correlation between the first components of the two datasets was *r_(47)_* = 0.94,  *p* < 2.2 x 10^-16^, and thus the weight of the design matrix in the DIABLO model classifying improvers vs non-improvers was set to 1. Plots to guide data
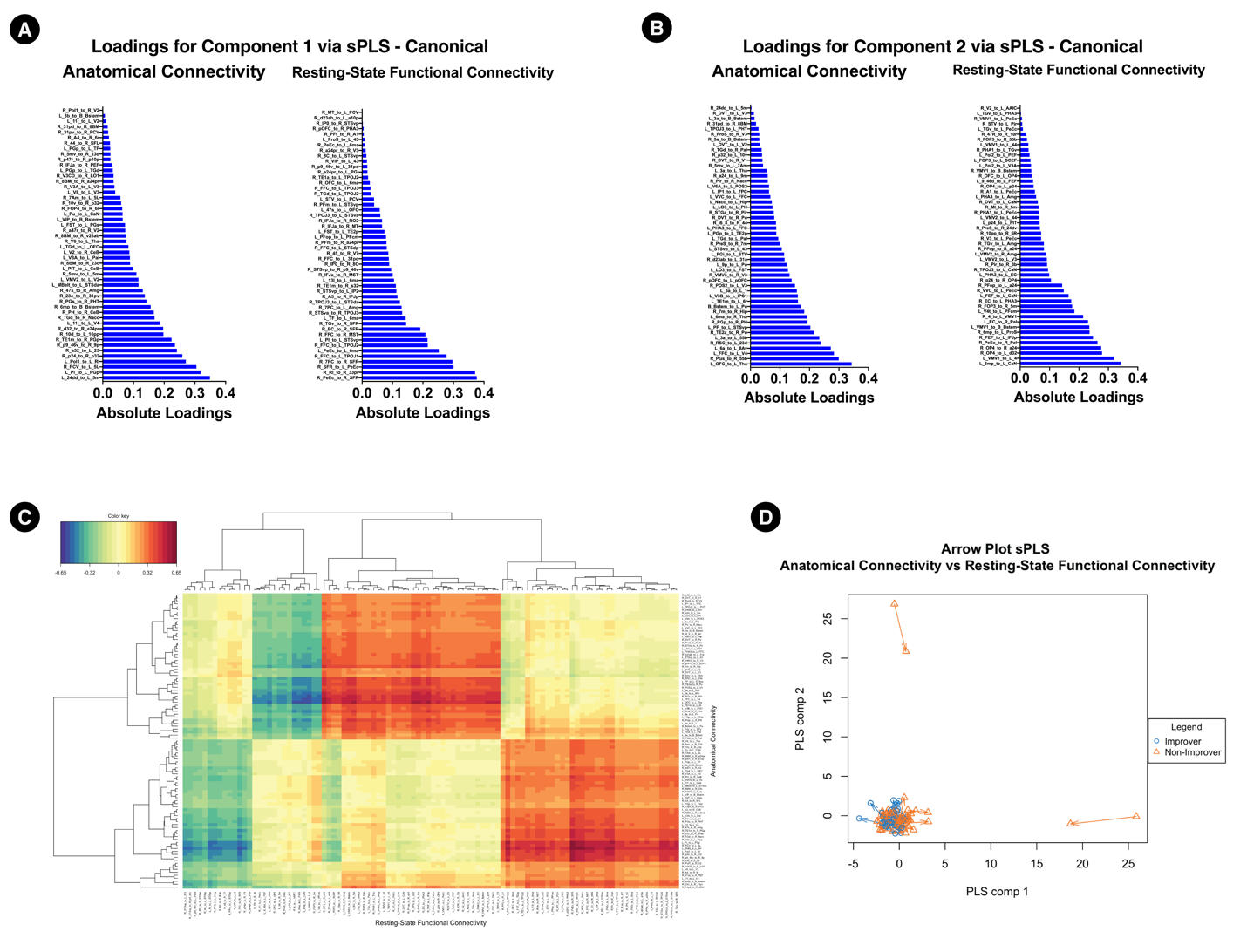
integration are shown below in **Fig S4**.

Fig S4: (A) Loadings plots for component 1 per data type, (B) Loadings plots for component 2 per data type, (C) Clustered image map showing the correlation structure between anatomical connectivity and resting-state functional connectivity, (D) Arrow plot showing agreeableness between datasets for each sample. Reference Supplementary Table 1 for regions of interest. sPLS shows strong correlation between datasets.

*sPLS between morphometry and clinical data for 3-month improvers and non-improvers*

A two component sPLS model was used with 50 features from the morphometry dataset and 10 features from the clinical dataset on each component. The correlation between the first components of the two datasets was *r_(47)_* = 0.85,  *p* < 8.5 x 10^-15^, and thus the weight of the design matrix in the DIABLO model classifying improvers vs non-improvers was set to 1. Plots to guide data integration are shown below in **Fig S5**.


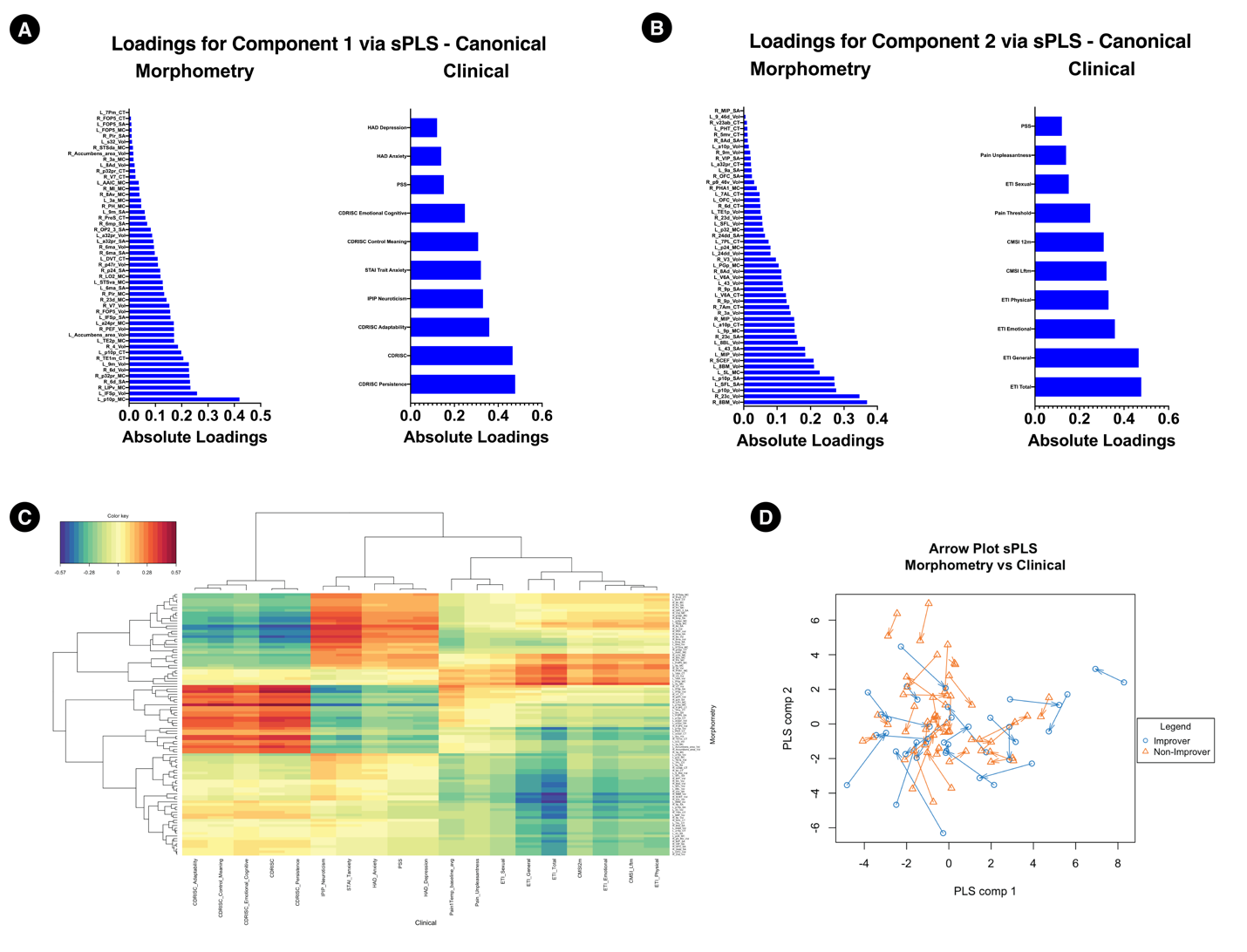


Fig S5: (A) Loadings plots for component 1 per data type, (B) Loadings plots for component 2 per data type, (C) Clustered image map showing the correlation structure between morphometry and clinical data, (D) Arrow plot showing agreeableness between datasets for each sample. Reference Supplementary Table 1 for regions of interest. sPLS shows strong correlation between datasets.

Abbreviations: Vol – volume, CT – cortical thickness, SA – surface area, MC – mean curvature

*sPLS between anatomical connectivity and clinical data for 3-month improvers and non-improvers*

A two component sPLS model was used with 50 features from the anatomical connectivity dataset and 10 features from the clinical dataset on each component. The correlation between the first components of the two datasets was *r_(47)_* = 0.91,  *p* < 2.2 x 10^-16^, and thus the weight of the design matrix in the DIABLO model classifying improvers vs non-improvers was set to 1. Plots to guide data integration are shown below in **Fig S6**.


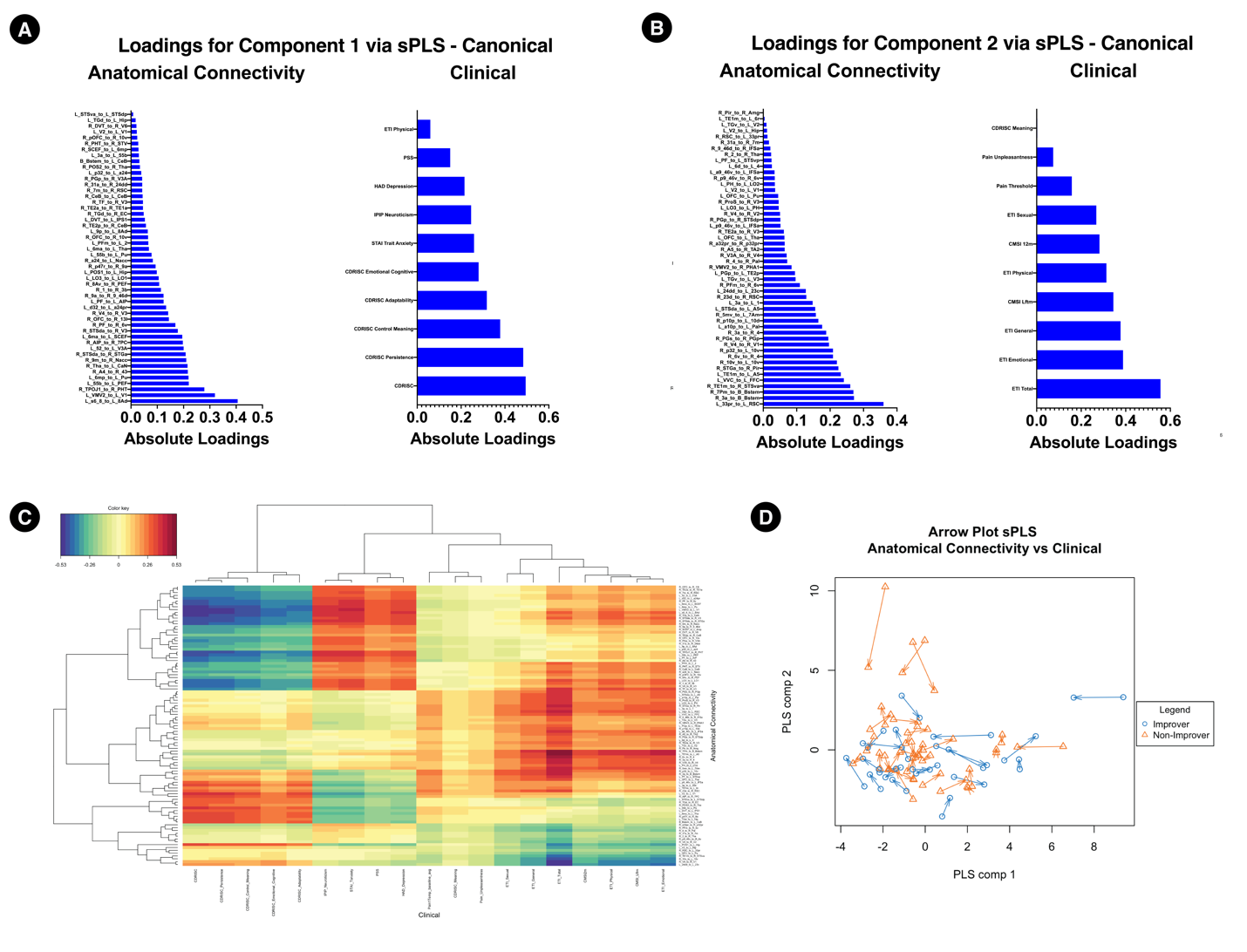


Fig S6: (A) Loadings plots for component 1 per data type, (B) Loadings plots for component 2 per data type, (C) Clustered image map showing the correlation structure between anatomical connectivity and clinical data, (D) Arrow plot showing agreeableness between datasets for each sample. Reference Supplementary Table 1 for regions of interest. sPLS shows strong correlation between datasets.

*sPLS between resting-state connectivity and clinical data for 3-month improvers and non-improvers*

A two component sPLS model was used with 50 features from the resting-state functional connectivity dataset and 10 features from the clinical dataset on each component. The correlation between the first components of the two datasets was *r_(47)_* = 0.96,  *p* < 2.2 x 10^-16^, and thus the weight of the design matrix in the DIABLO model classifying improvers vs non-improvers was set to 1. Plots to guide data integration are shown below in **Fig S7**.


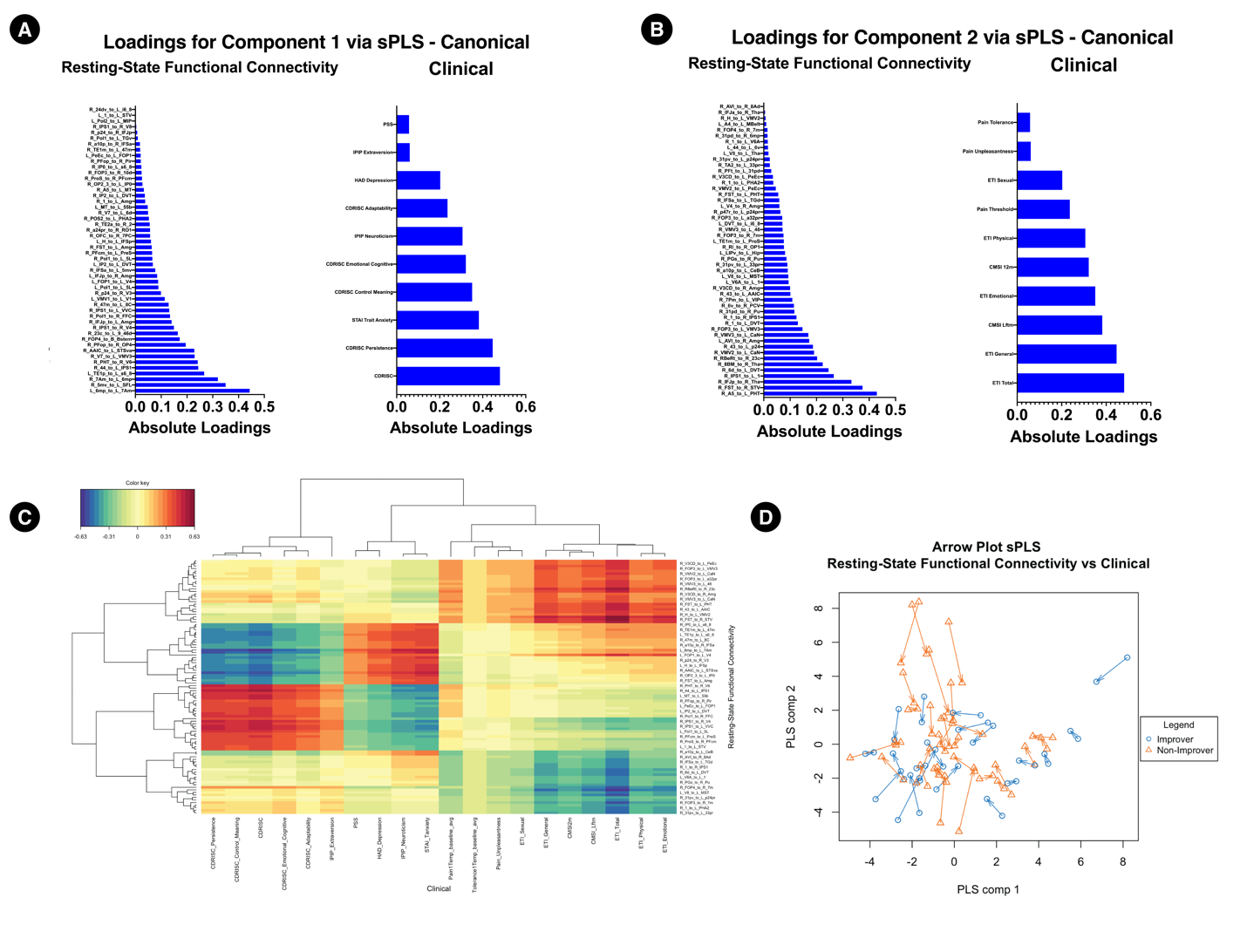


Fig S7: (A) Loadings plots for component 1 per data type, (B) Loadings plots for component 2 per data type, (C) Clustered image map showing the correlation structure between resting-state functional connectivity and clinical data, (D) Arrow plot showing agreeableness between datasets for each sample. Reference Supplementary Table 1 for regions of interest. sPLS shows strong correlation between datasets.

*sPLS between morphometry and anatomical connectivity data for 12-month improvers and non-improvers*

A two component sPLS model was used with 50 features from each dataset on each component. The correlation between the first components of the two datasets was *r_(29)_* = 0.91,  *p* < 6.7 x 10^-13^, and thus the weight of the design matrix in the DIABLO model classifying improvers vs non-improvers was set to 1. Plots to guide data integration are shown below in **Fig S8**.


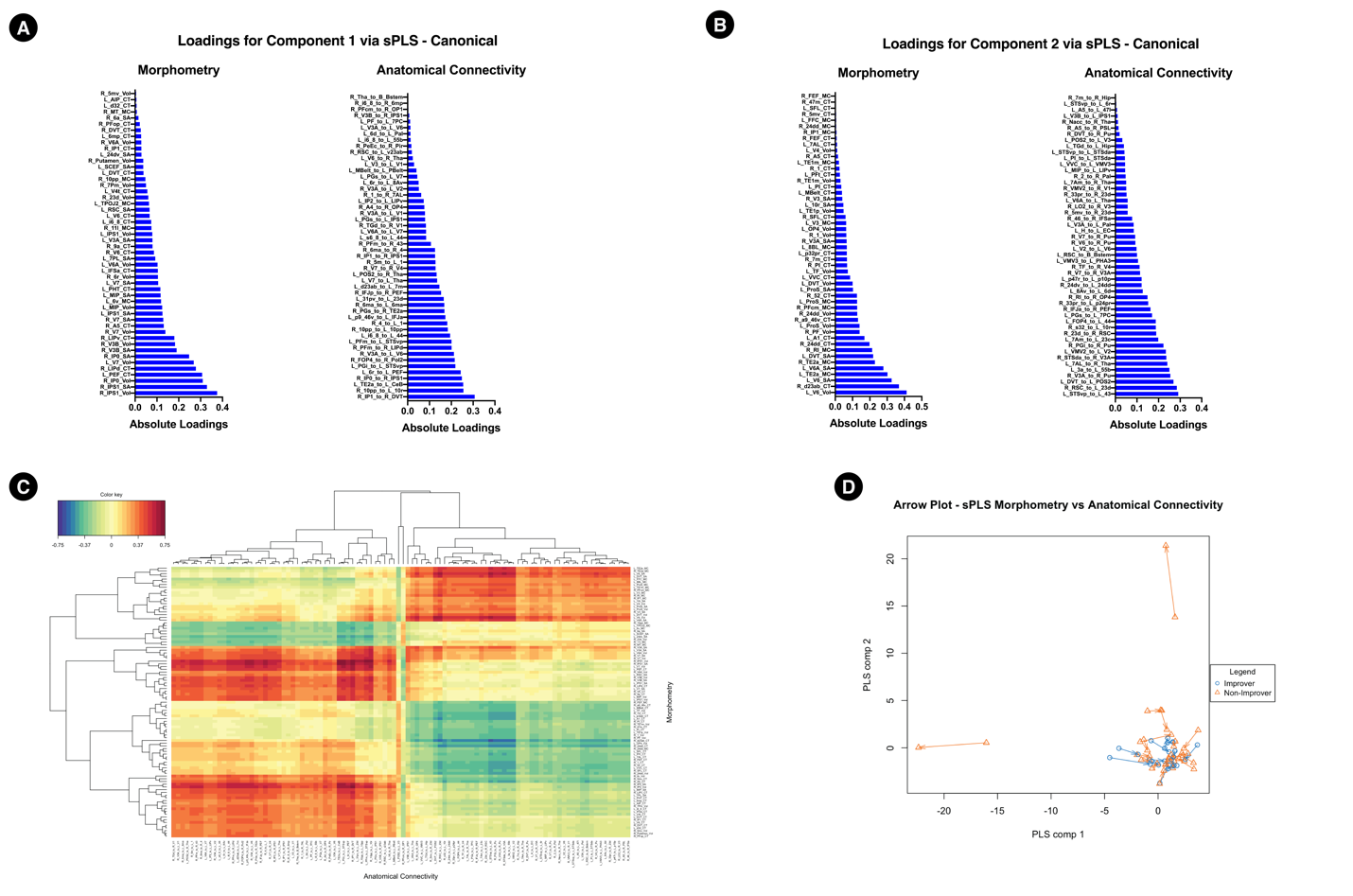


Fig S8: (A) Loadings plots for component 1 per data type, (B) Loadings plots for component 2 per data type, (C) Clustered image map showing the correlation structure between morphometry and anatomical connectivity, (D) Arrow plot showing agreeableness between datasets for each sample. Reference Supplementary Table 1 for regions of interest. sPLS shows strong correlation between datasets.

*sPLS between morphometry and resting-state functional connectivity data for 12-month improvers and non-improvers*

A two component sPLS model was used with 50 features from each dataset on each component. The correlation between the first components of the two datasets was *r_(29)_* = 0.98,  *p* < 2.2 x 10^-16^, and thus the weight of the design matrix in the DIABLO model classifying improvers vs non-improvers was set to 1. Plots to guide data integration are shown below in **Fig S9**.


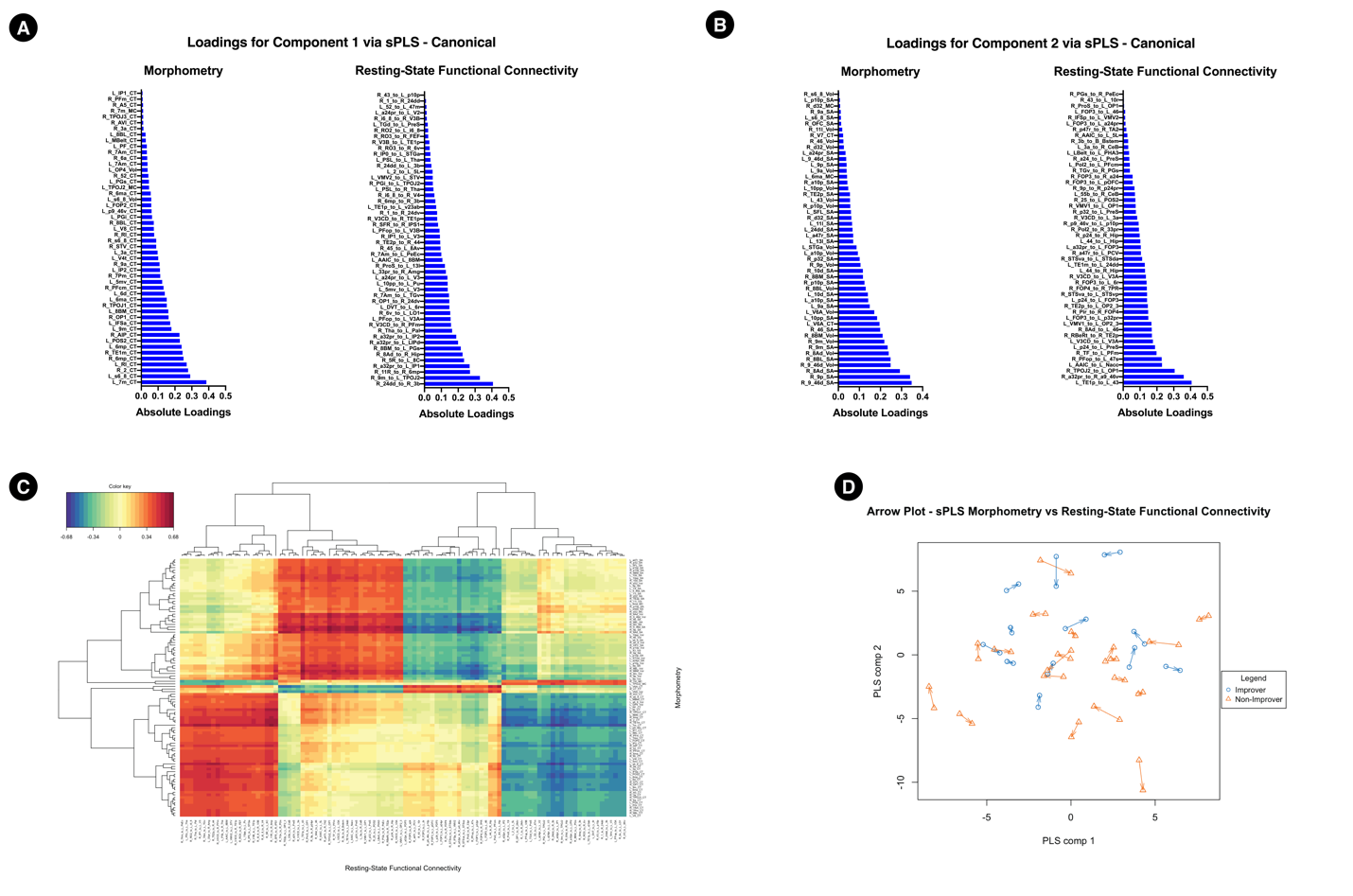


Fig S9: (A) Loadings plots for component 1 per data type, (B) Loadings plots for component 2 per data type, (C) Clustered image map showing the correlation structure between morphometry and resting-state functional connectivity, (D) Arrow plot showing agreeableness between datasets for each sample. Reference Supplementary Table 1 for regions of interest. sPLS shows strong correlation between datasets.

*sPLS between anatomical connectivity and resting-state functional connectivity data for 12-month improvers and non-improvers*

A two component sPLS model was used with 50 features from each dataset on each component. The correlation between the first components of the two datasets was *r_(29)_* = 0.98,  *p* < 2.2 x 10^-16^, and thus the weight of the design matrix in the DIABLO model classifying improvers vs non-improvers was set to 1. Plots to guide data integration are shown below in **Fig S10**.

*
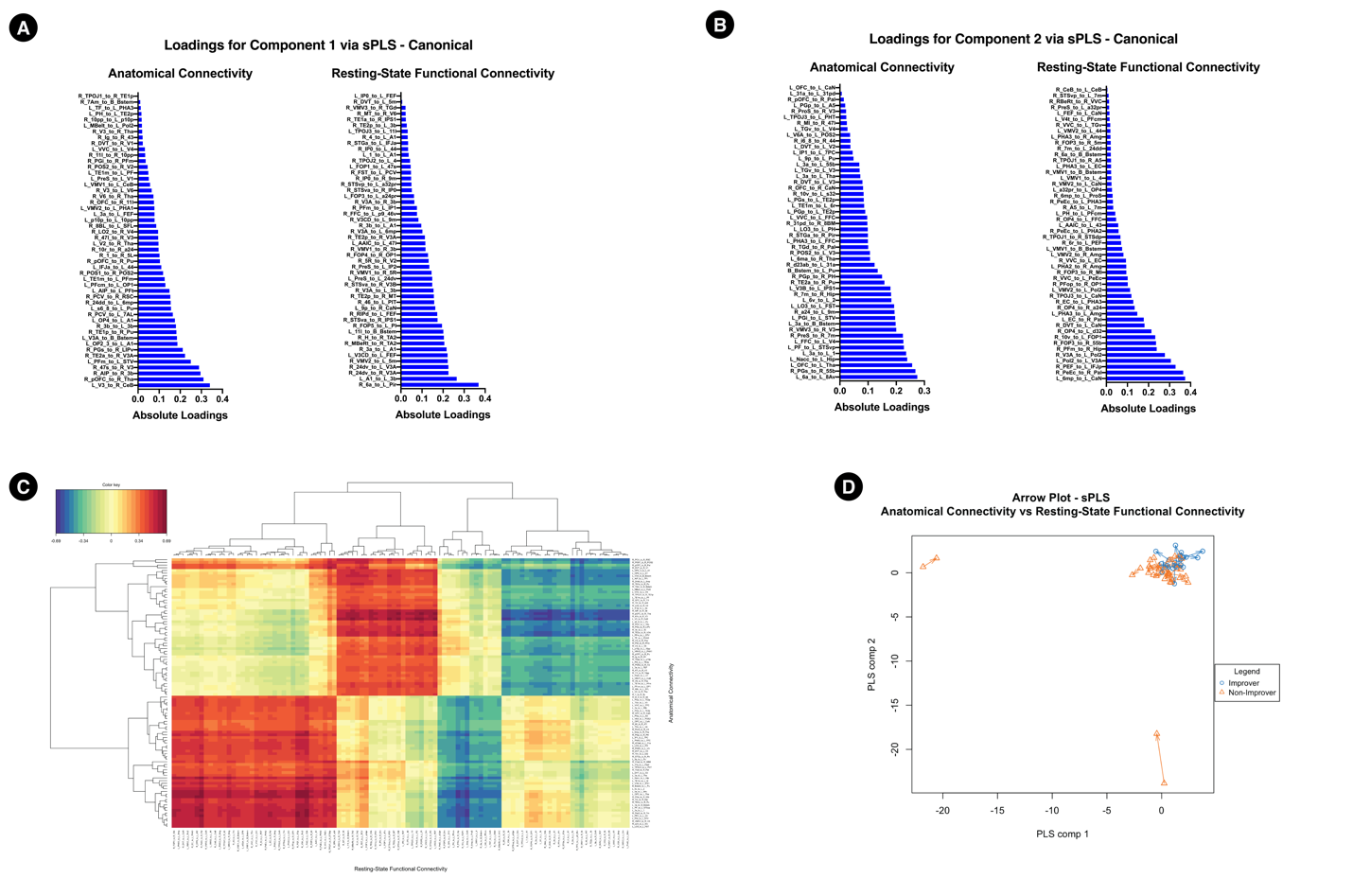
*

Fig S10: (A) Loadings plots for component 1 per data type, (B) Loadings plots for component 2 per data type, (C) Clustered image map showing the correlation structure between anatomical connectivity and resting-state functional connectivity, (D) Arrow plot showing agreeableness between datasets for each sample. Reference Supplementary Table 1 for regions of interest. sPLS shows strong correlation between datasets.

*sPLS between morphometry and clinical data for 12-month improvers and non-improvers*

A two component sPLS model was used with 50 features from the morphometry dataset and 10 features from the clinical dataset on each component. The correlation between the first components of the two datasets was *r_(29)_* = 0.90,  *p* < 9.5 x 10^-12^, and thus the weight of the design matrix in the DIABLO model classifying improvers vs non-improvers was set to 1. Plots to guide data integration are shown below in **Fig S11**.

*
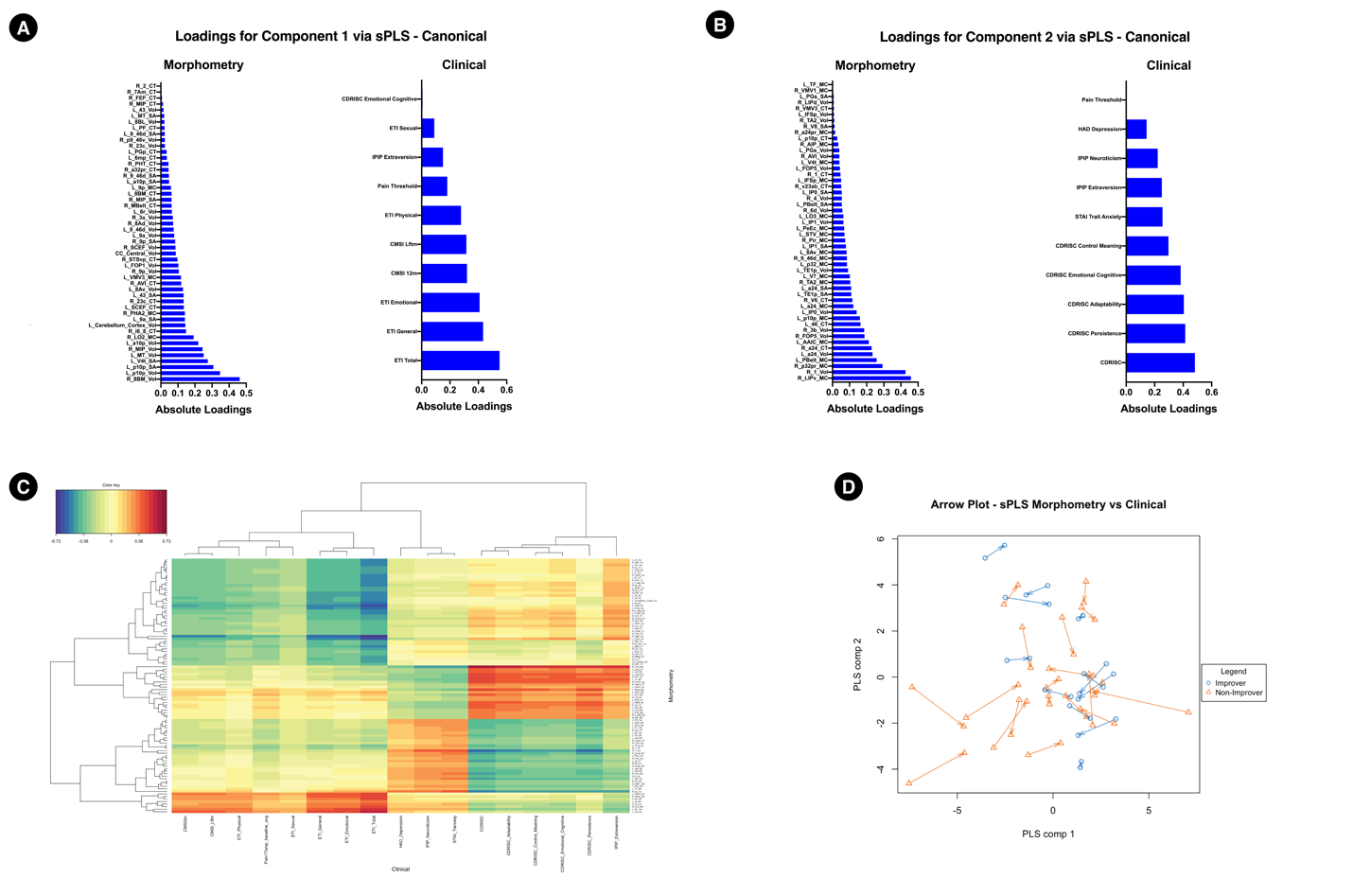
*

Fig S11: (A) Loadings plots for component 1 per data type, (B) Loadings plots for component 2 per data type, (C) Clustered image map showing the correlation structure between morphometry and clinical data, (D) Arrow plot showing agreeableness between datasets for each sample. Reference Supplementary Table 1 for regions of interest. sPLS shows strong correlation between datasets.

*sPLS between anatomical connectivity and clinical data for 12-month improvers and non-improvers*

A two component sPLS model was used with 50 features from the anatomical connectivity dataset and 10 features from the clinical dataset on each component. The correlation between the first components of the two datasets was *r_(29)_* = 0.92,  *p* < 2.3 x 10^-13^, and thus the weight of the design matrix in the DIABLO model classifying improvers vs non-improvers was set to 1. Plots to guide data integration are shown below in **Fig S12**.

*
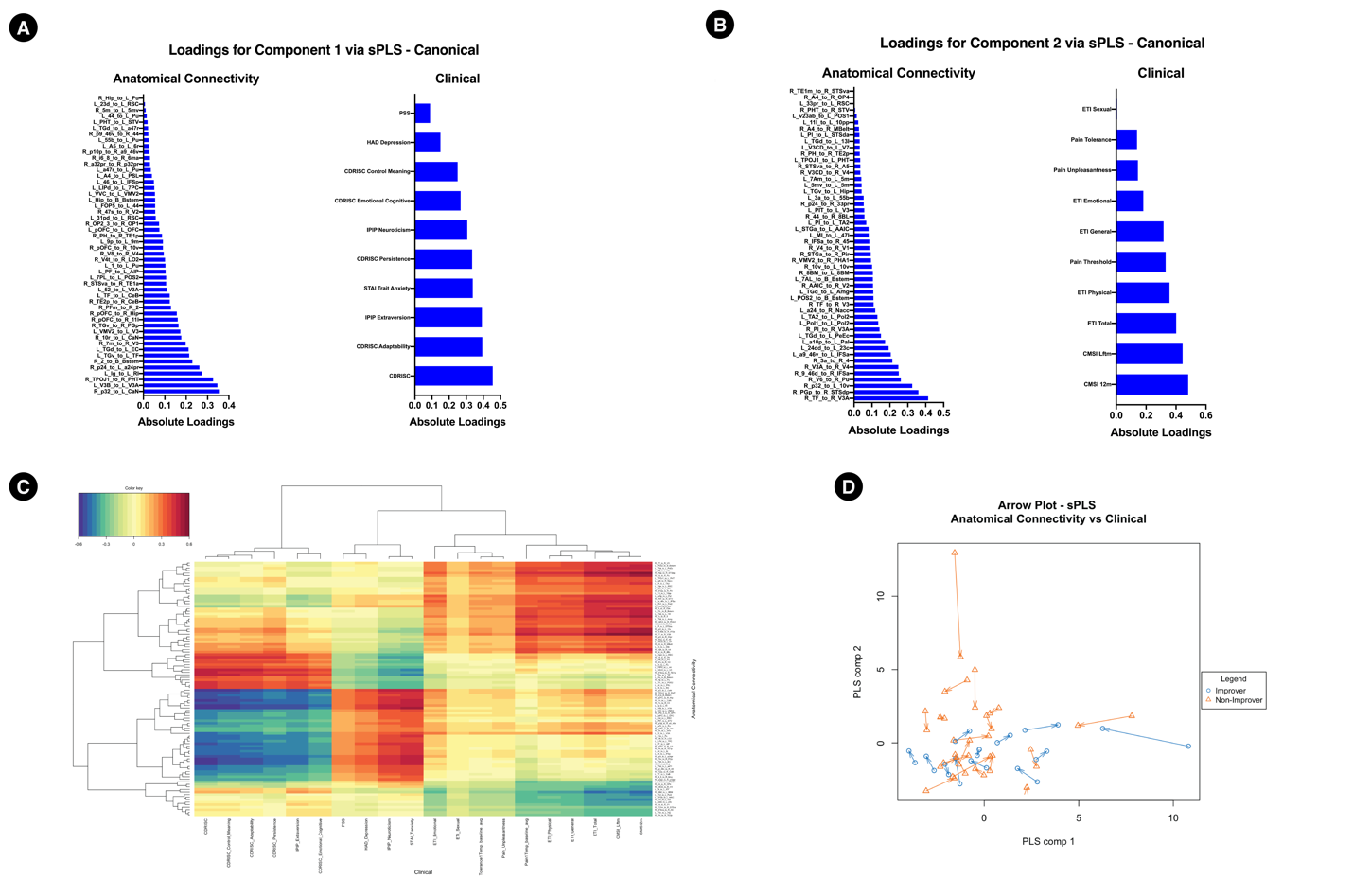
*

Fig S12: (A) Loadings plots for component 1 per data type, (B) Loadings plots for component 2 per data type, (C) Clustered image map showing the correlation structure between anatomical connectivity and clinical data, (D) Arrow plot showing agreeableness between datasets for each sample. Reference Supplementary Table 1 for regions of interest. sPLS shows strong correlation between datasets.

*sPLS between resting-state connectivity and clinical data for 12-month improvers and non-improvers*

A two component sPLS model was used with 50 features from the resting-state functional connectivity dataset and 10 features from the clinical dataset on each component. The correlation between the first components of the two datasets was *r_(29)_* = 0.96,  *p* < 2.2 x 10^-16^, and thus the weight of the design matrix in the DIABLO model classifying improvers vs non-improvers was set to 1. Plots to guide data integration are shown below in **Fig S13**.

*
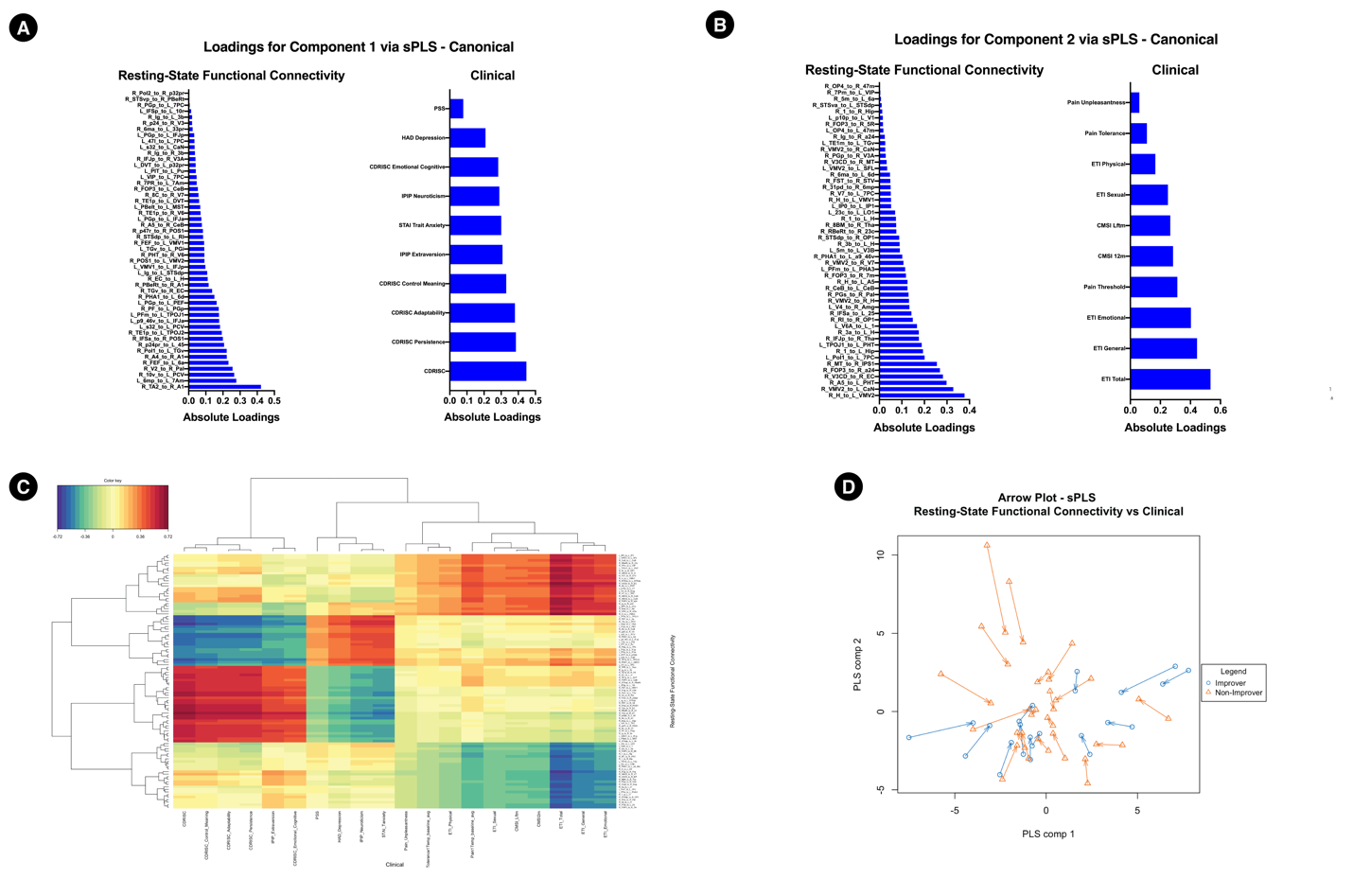
*

Fig S13: (A) Loadings plots for component 1 per data type, (B) Loadings plots for component 2 per data type, (C) Clustered image map showing the correlation structure between resting-state functional connectivity and clinical data, (D) Arrow plot showing agreeableness between datasets for each sample. Reference Supplementary Table 1 for regions of interest. sPLS shows strong correlation between datasets.

**Supplementary Table 3**

| **Block** | **Component** | **AUC** | **p-value** |
| --- | --- | --- | --- |
| Morphometry | 1 | 0.750 | 0.003 |
|  | 2 | 0.812 | 0.0002 |
|  | 3 | 0.840 | 6.140e-05 |
| Anatomical Connectivity | 1 | 0.736 | 0.005 |
|  | 2 | 0.757 | 0.002 |
|  | 3 | 0.869 | 1.342e-05 |
| Resting-State Functional Connectivity | 1 | 0.812 | 0.0002 |
|  | 2 | 0.912 | 1.050e-06 |
|  | 3 | 0.967 | 3.534e-08 |
| Behavioral/Clinical | 1 | 0.616 | 0.170 |
|  | 2 | 0.681 | 0.033 |
|  | 3 | 0.745 | 0.004 |
| Combined | 1 | 0.790 | 0.013 |
|  | 2 | 0.845 | 0.003 |
|  | 3 | 0.887 | 0.001 |

Area under the curve (AUC) for the receiving operating characteristic per block and component, along with the full combined DIABLO model predicting 3-month symptom changes. The p-value results from a Wilcoxon test comparing the classes improvers and non-improvers.

**Supplementary Table 4**

| **Block** | **Component** | **AUC** | **p-value** |
| --- | --- | --- | --- |
| Morphometry | 1 | 0.675 | 0.105 |
|  | 2 | 0.873 | 0.0006 |
| Anatomical Connectivity | 1 | 0.838 | 0.002 |
|  | 2 | 0.895 | 0.0003 |
| Resting-State Functional Connectivity | 1 | 0.790 | 0.007 |
|  | 2 | 0.877 | 0.0005 |
| Behavioral/Clinical | 1 | 0.561 | 0.570 |
|  | 2 | 0.719 | 0.043 |
| Combined | 1 | 0.784 | 0.116 |
|  | 2 | 0.883 | 0.023 |

Area under the curve for the receiving operating characteristic per block and component, along with the full combined DIABLO model predicting 12-month symptom changes. The p-value results from a Wilcoxon test comparing the classes improvers and non-improvers.

***Distribution of Selected Markers by DIABLO: 3 months***

The figures below represent the distribution of the features selected by DIABLO per group. All features were scaled and centered.


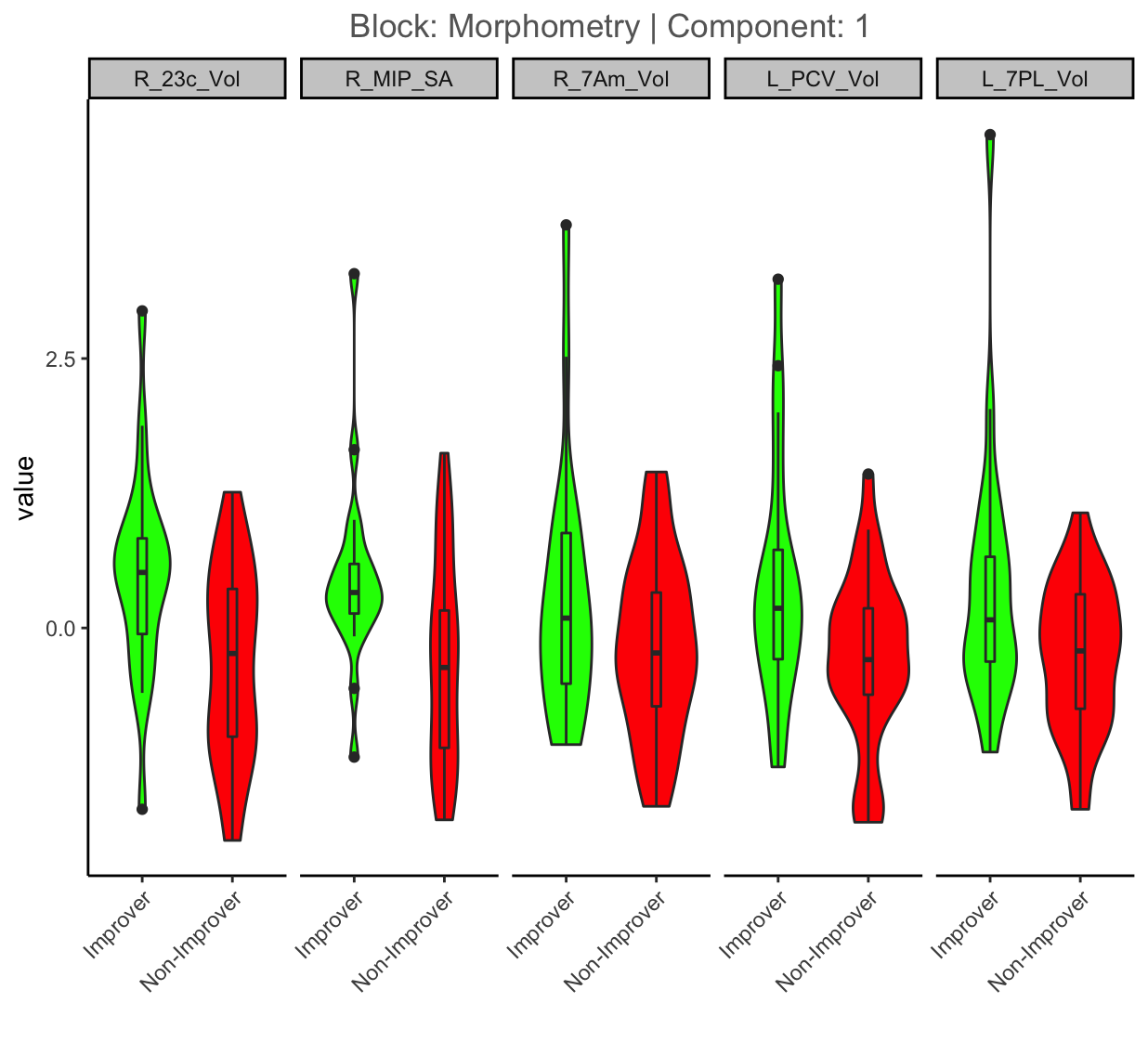


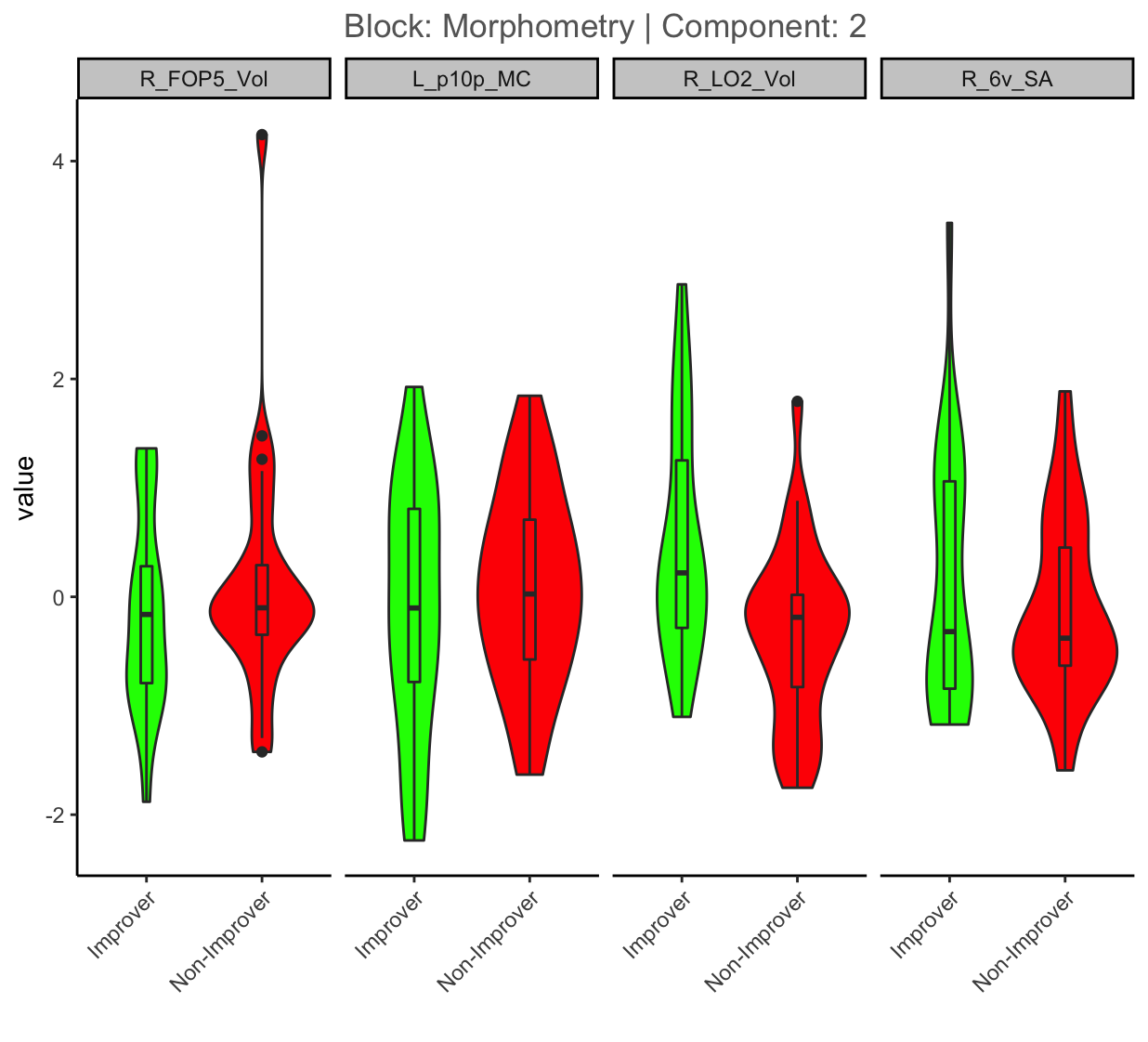

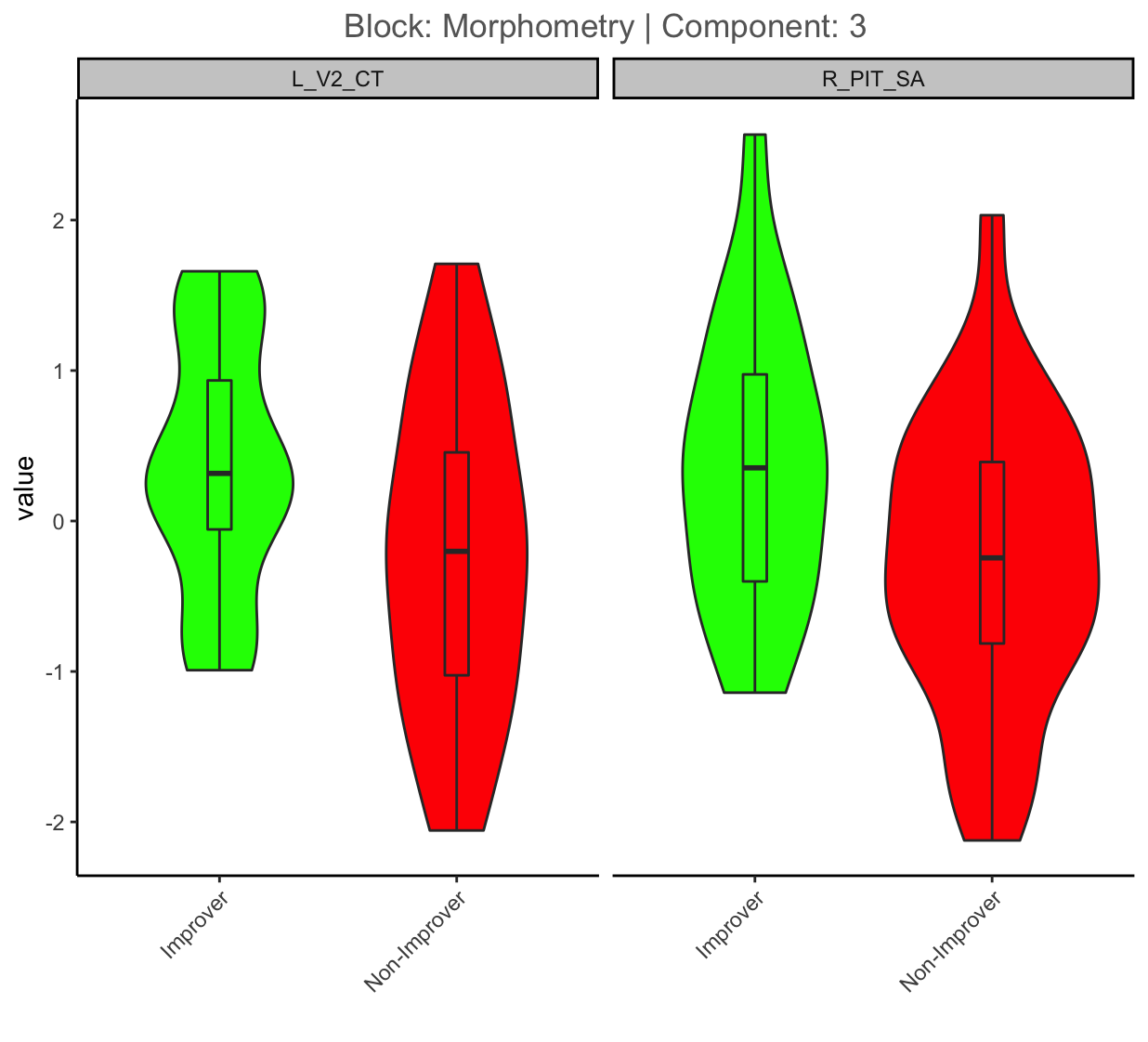


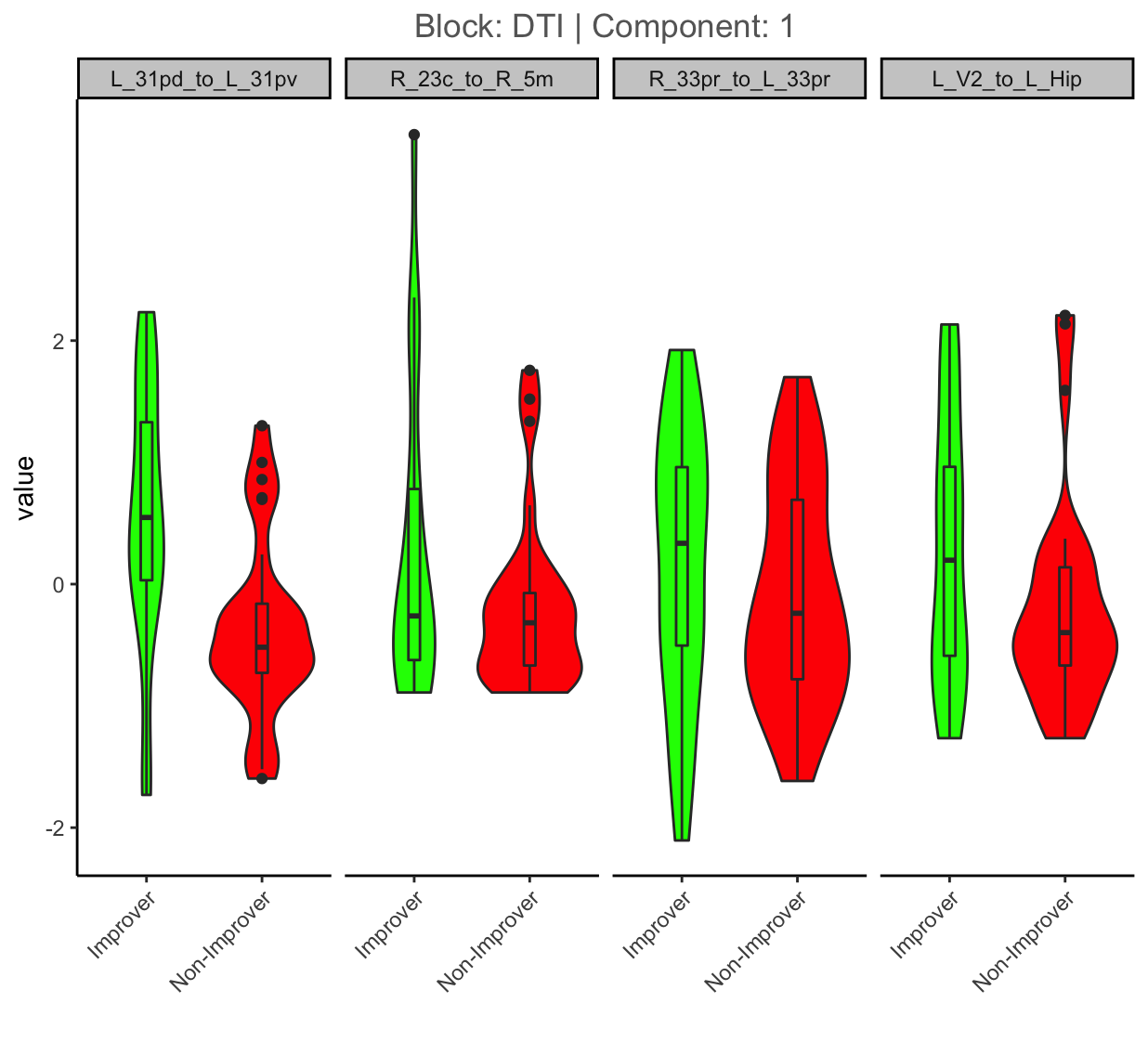

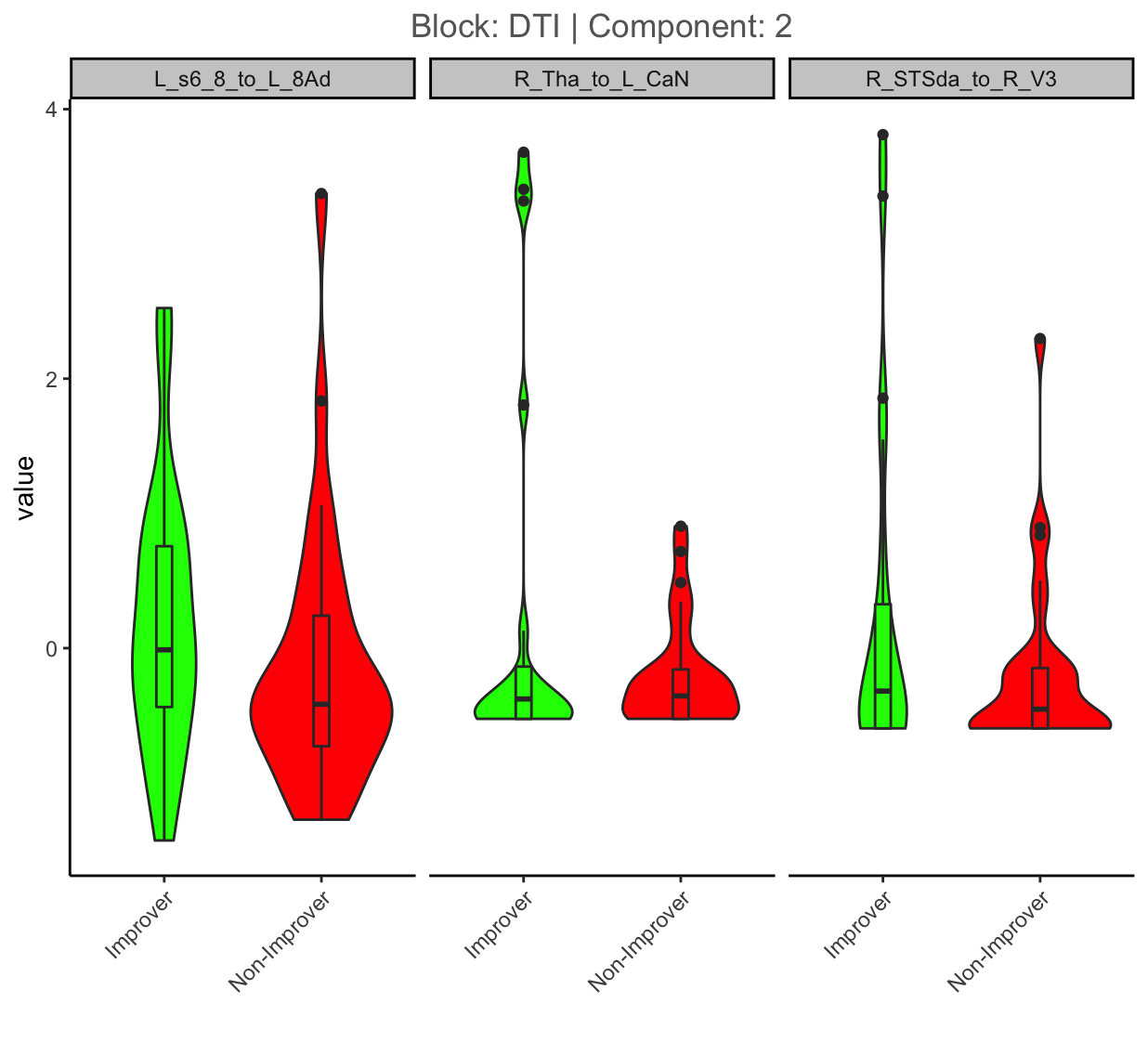

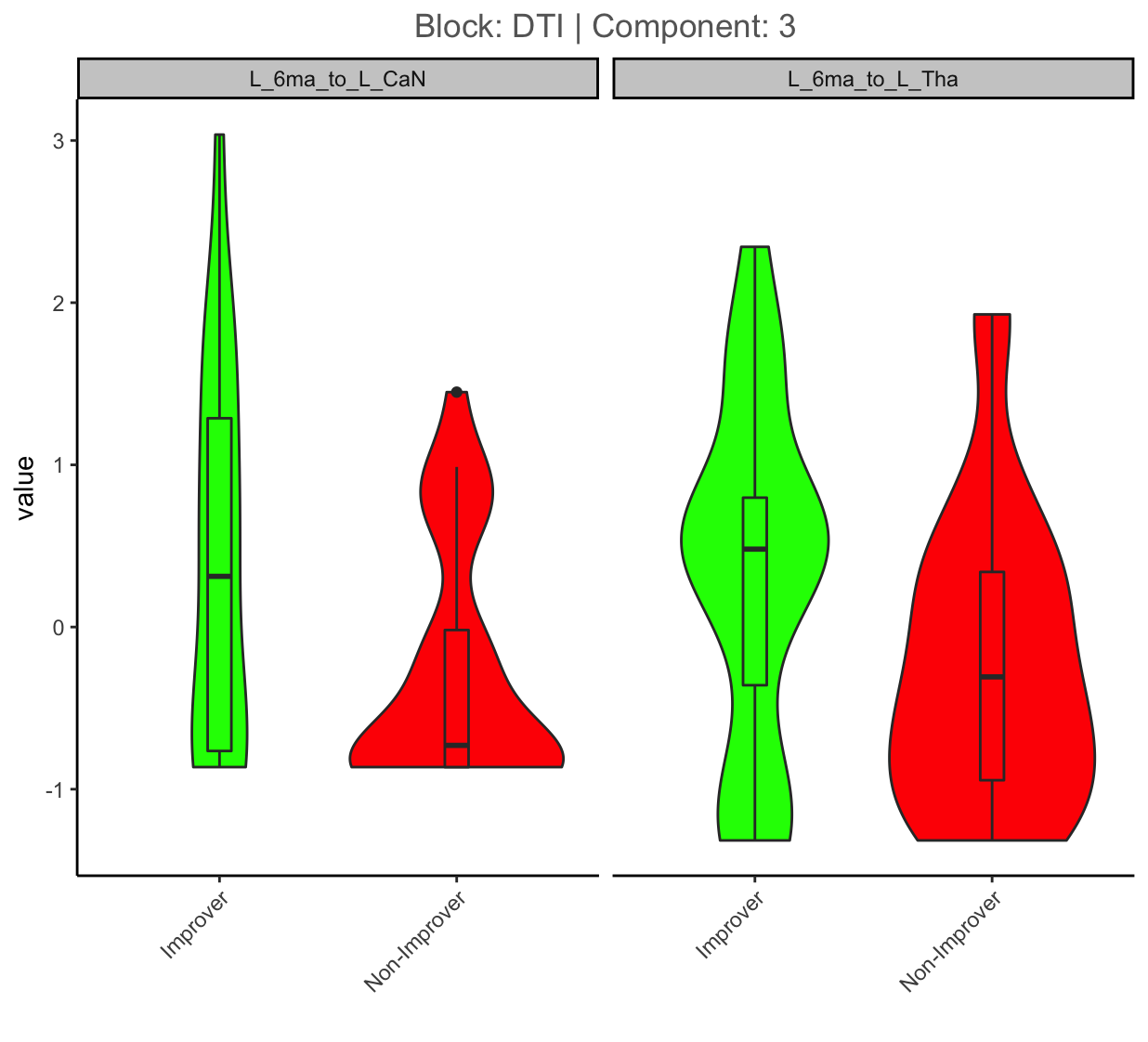

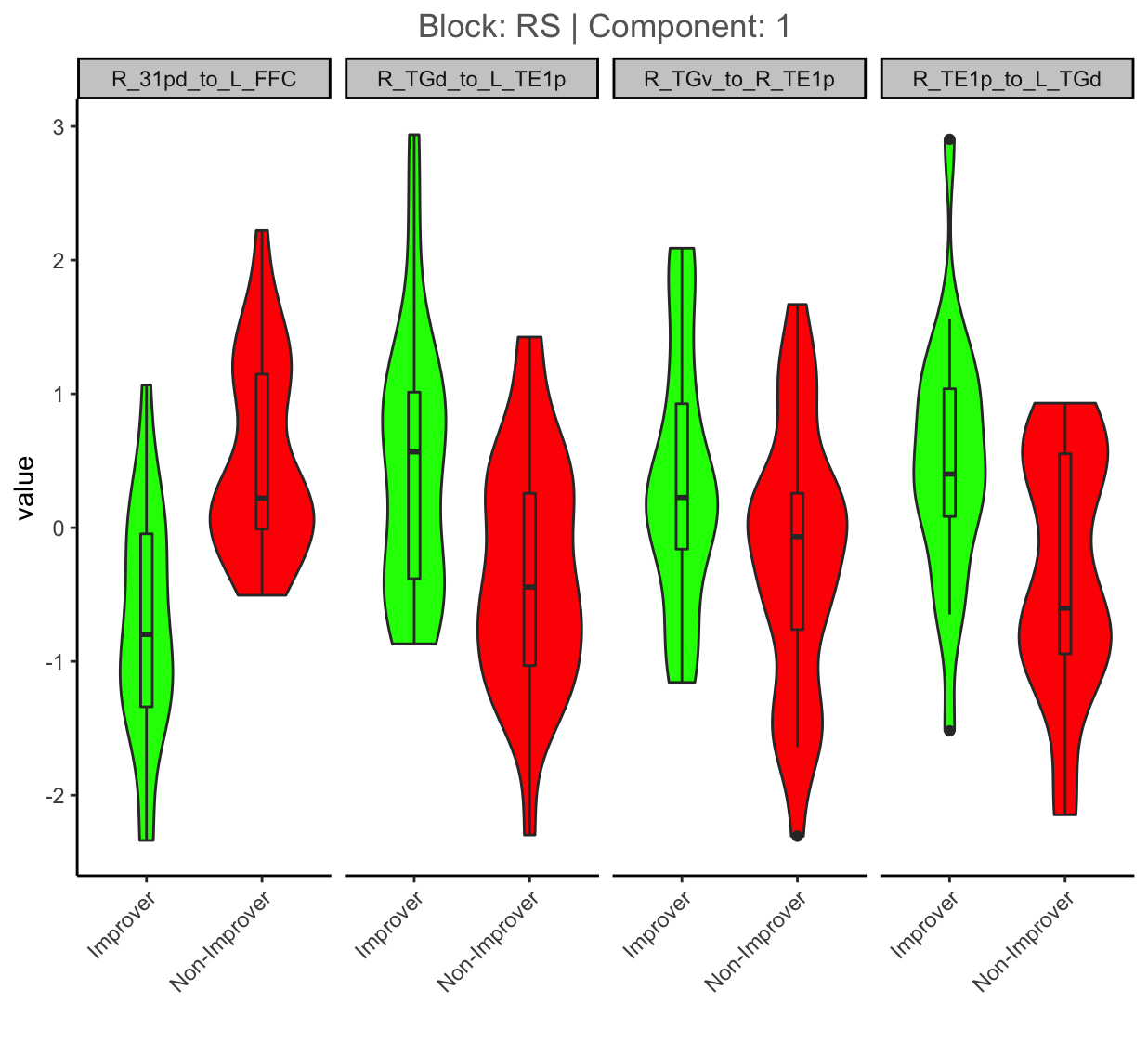

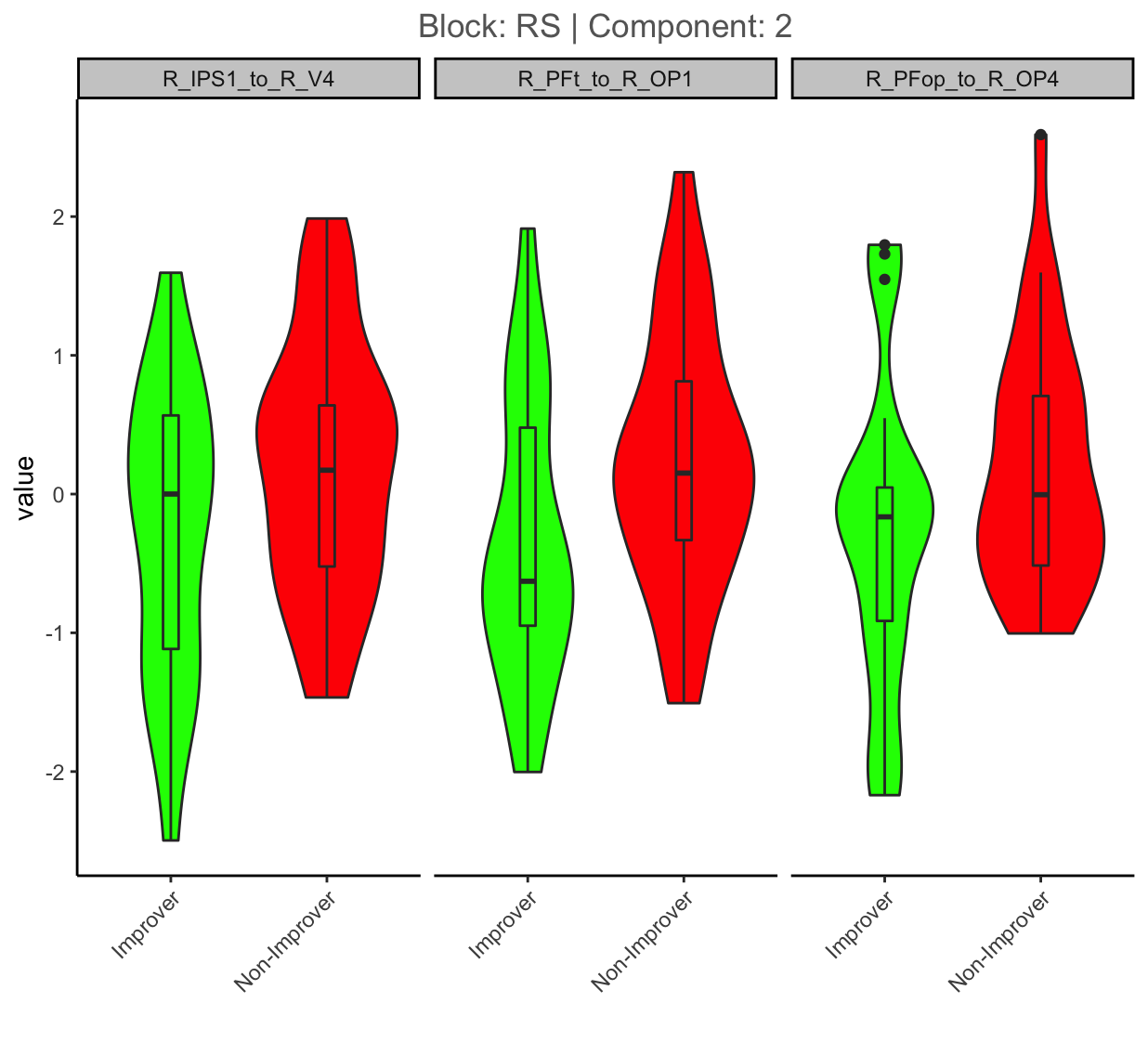

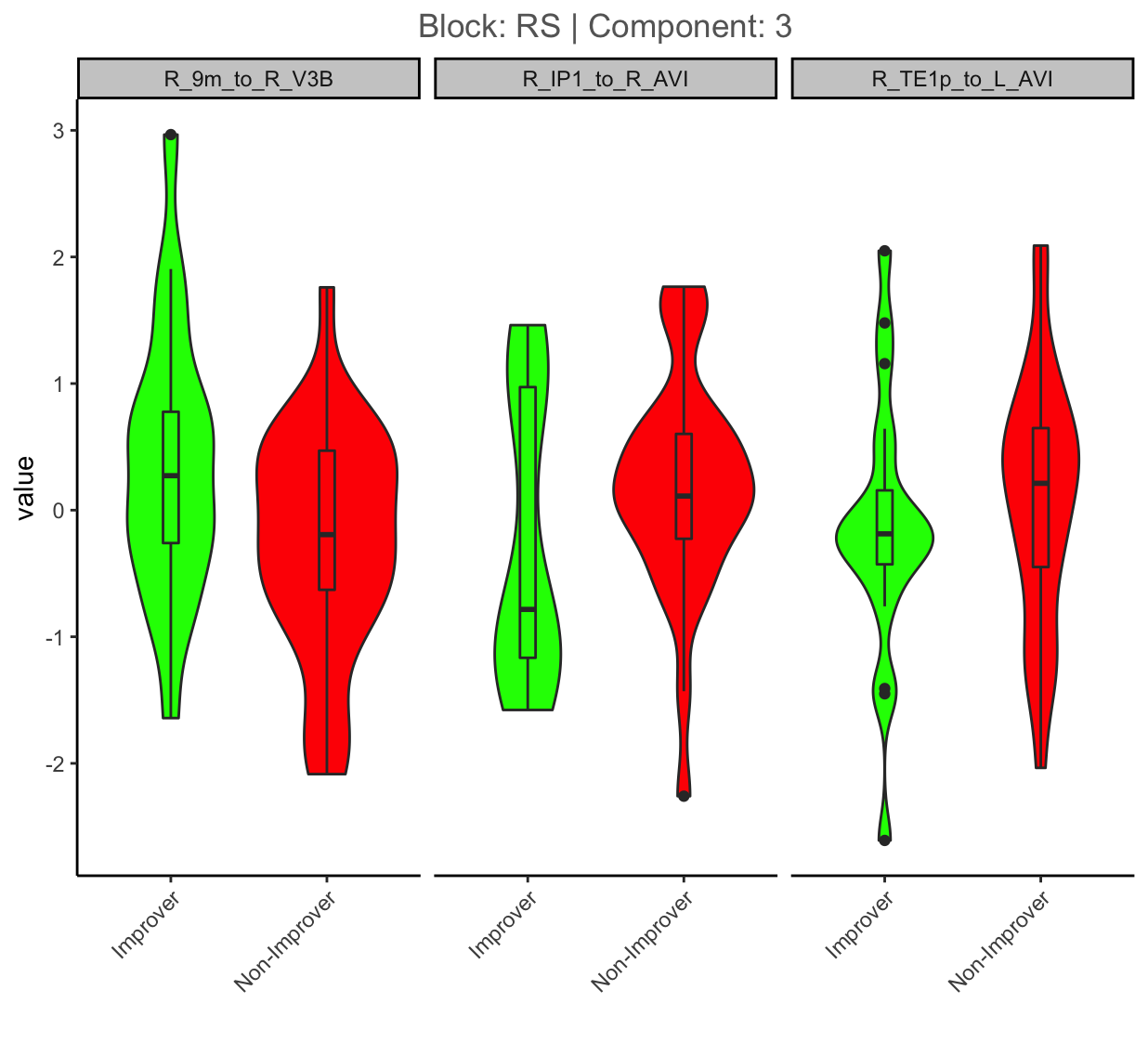

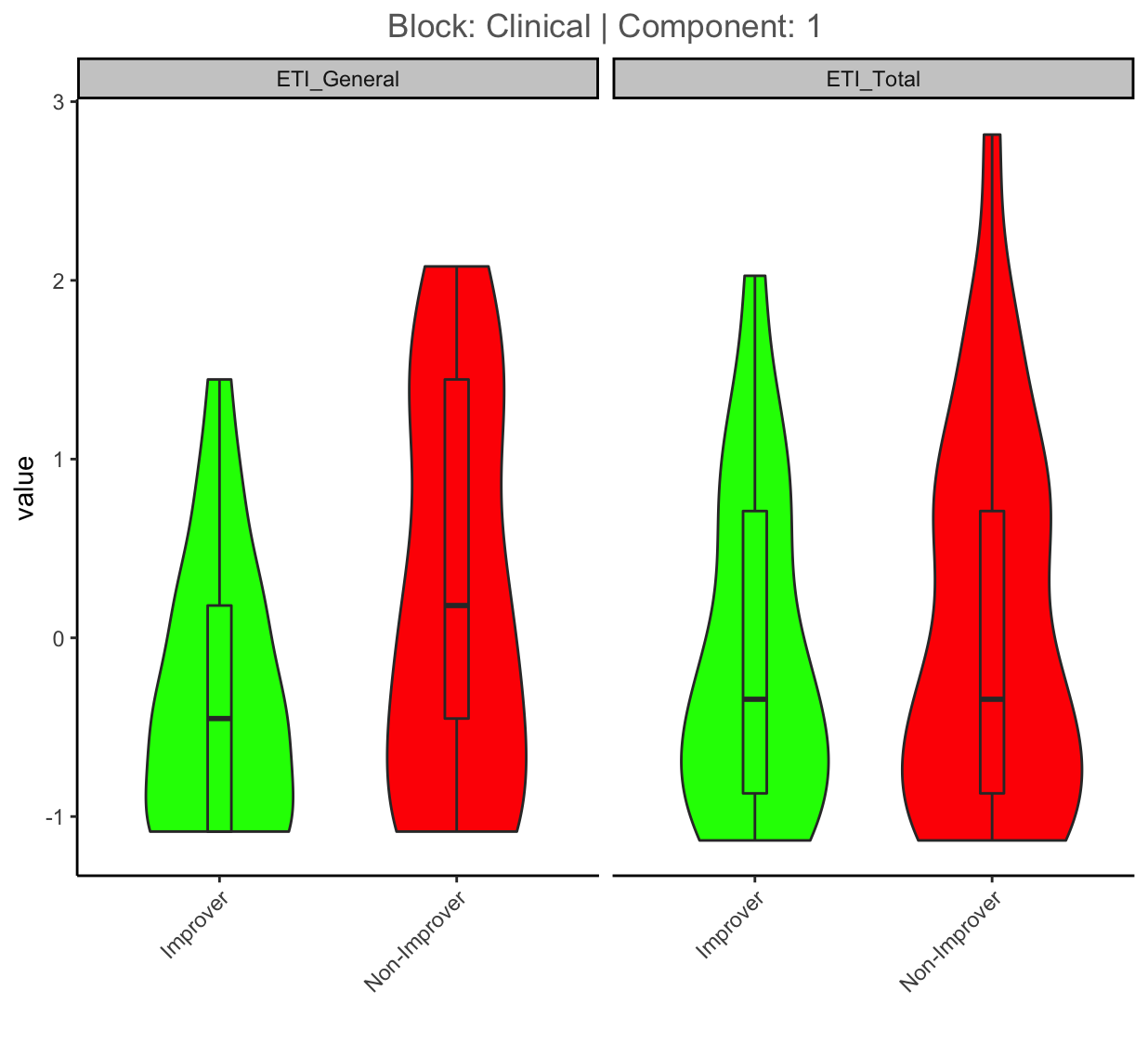

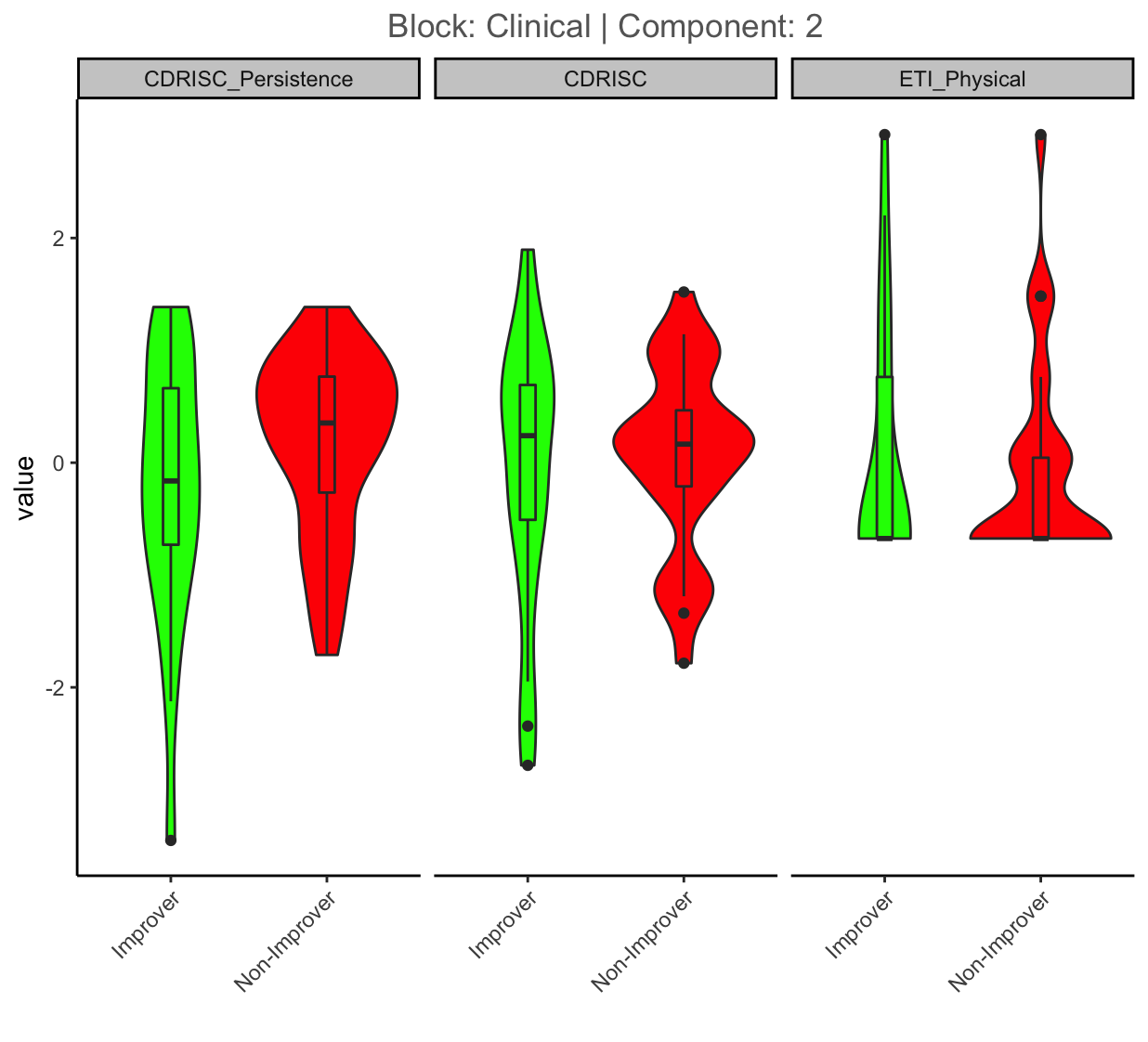

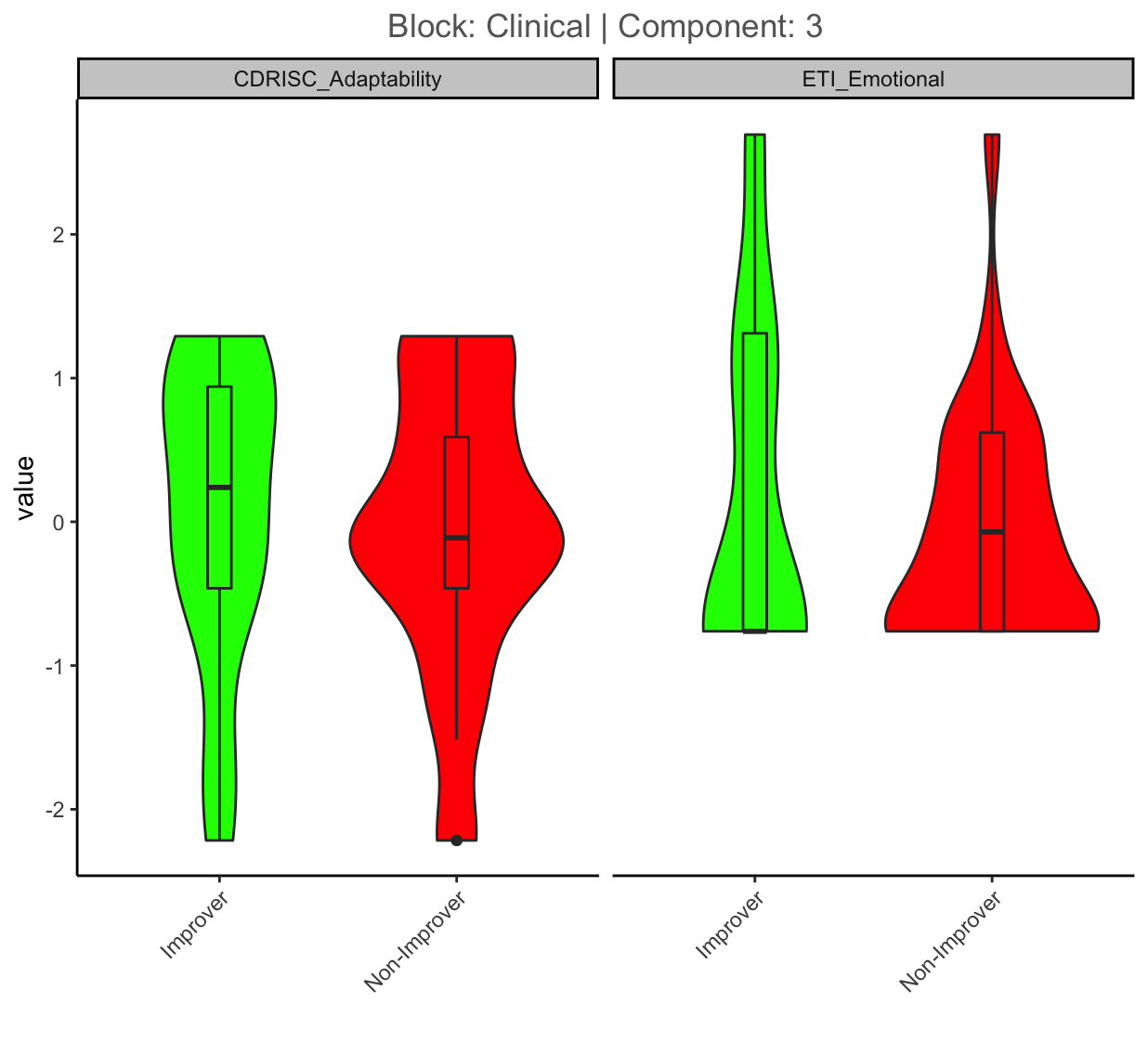


***Distribution of Selected Markers by DIABLO: 12 months***


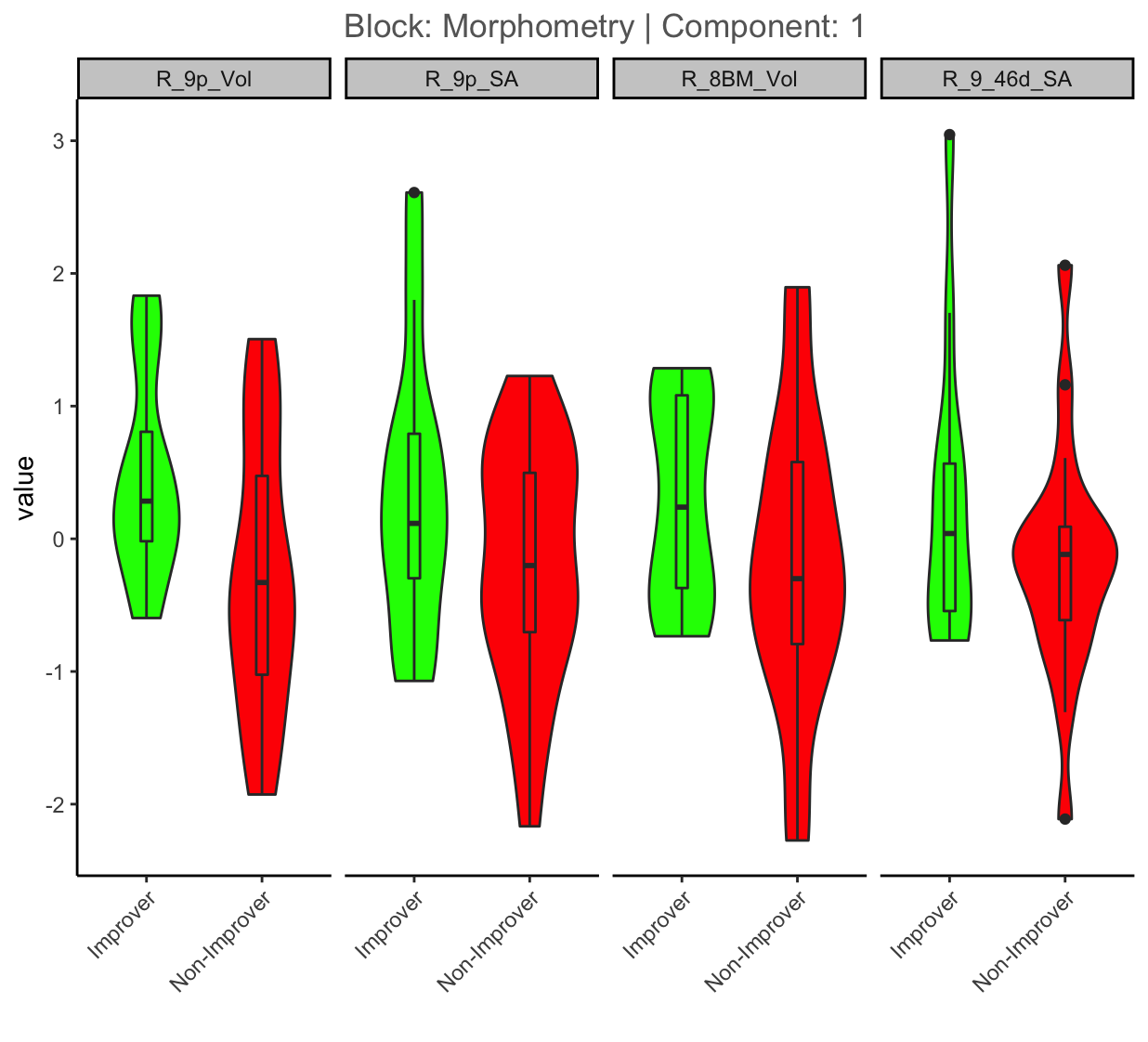

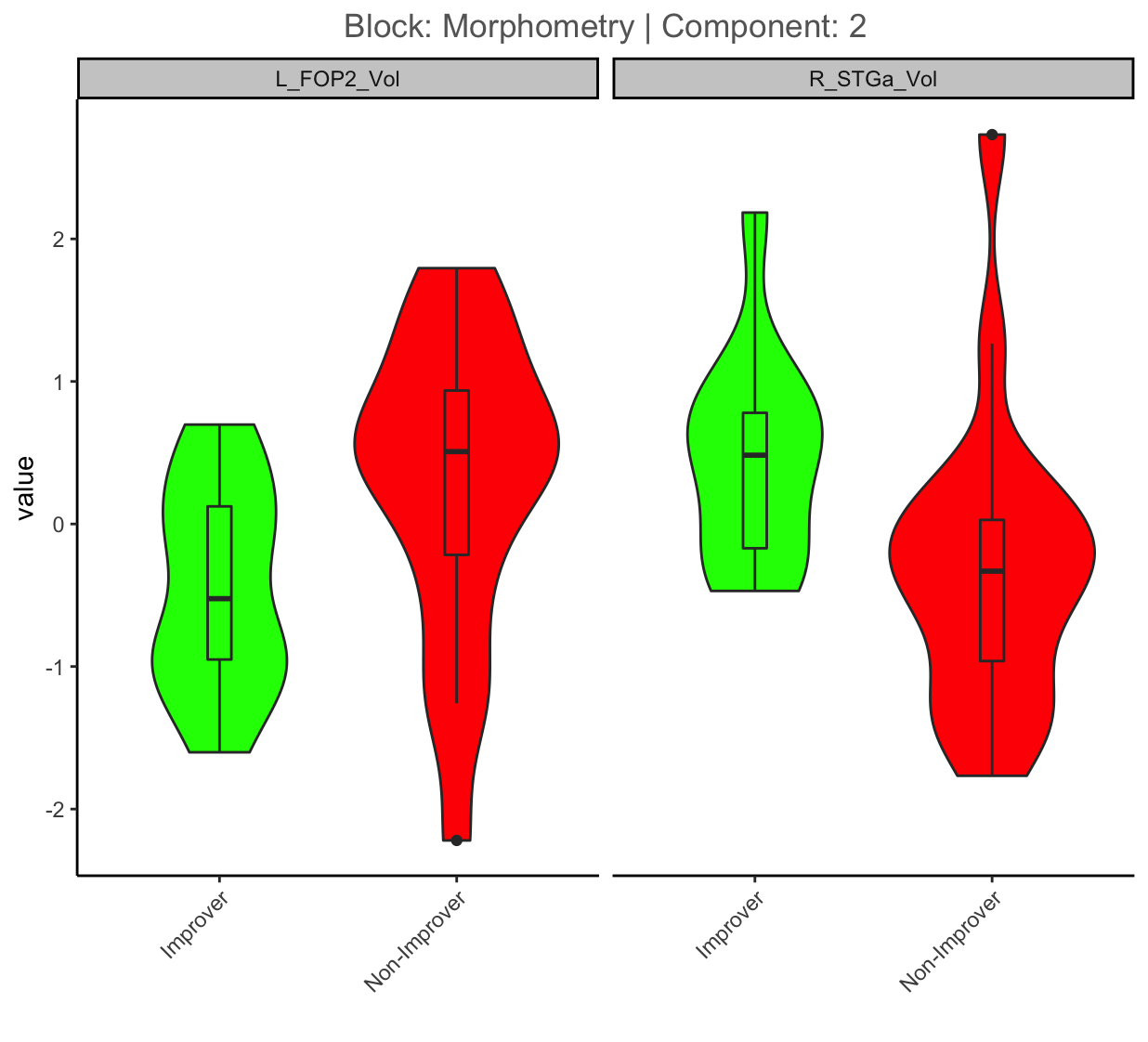

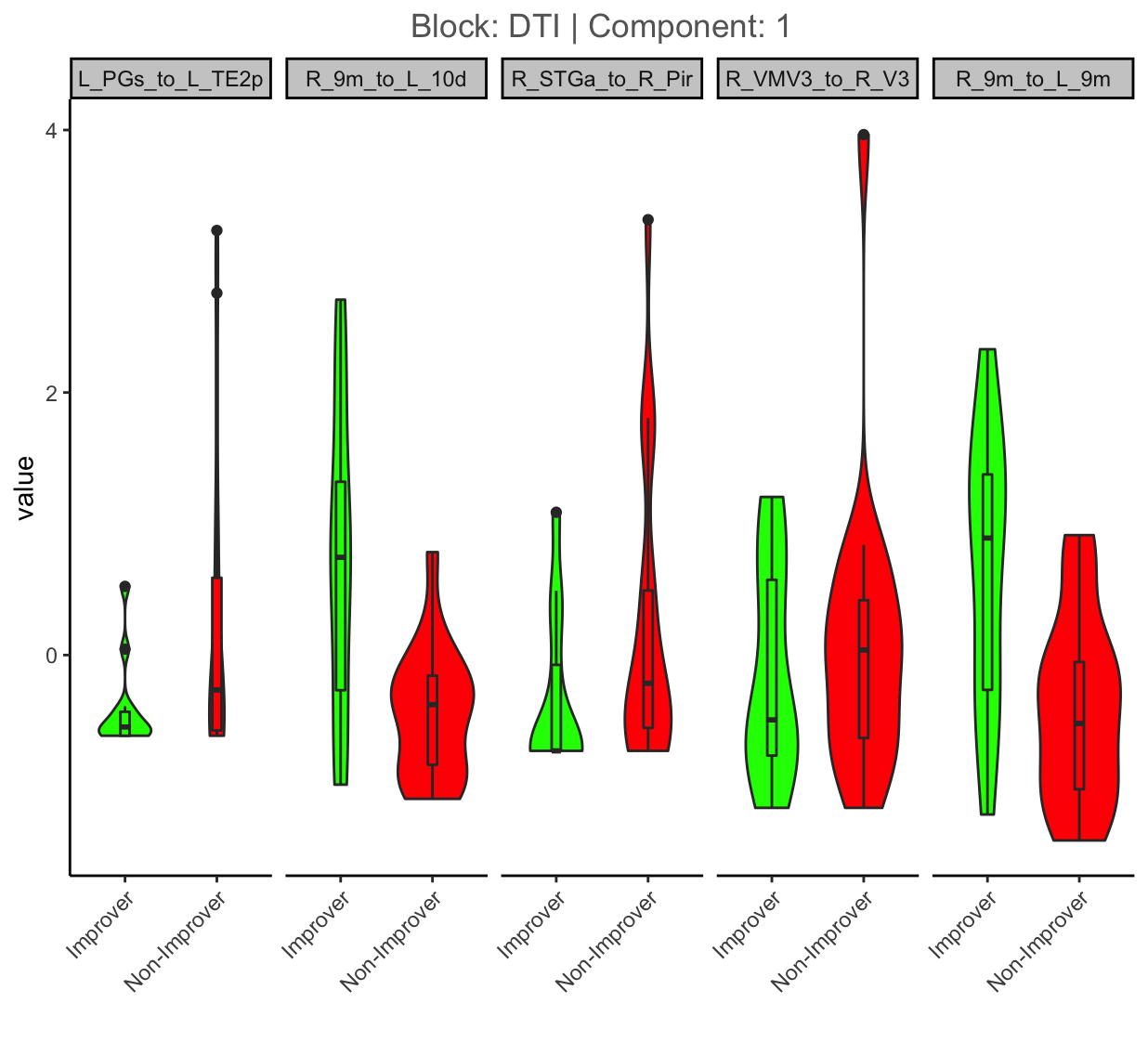

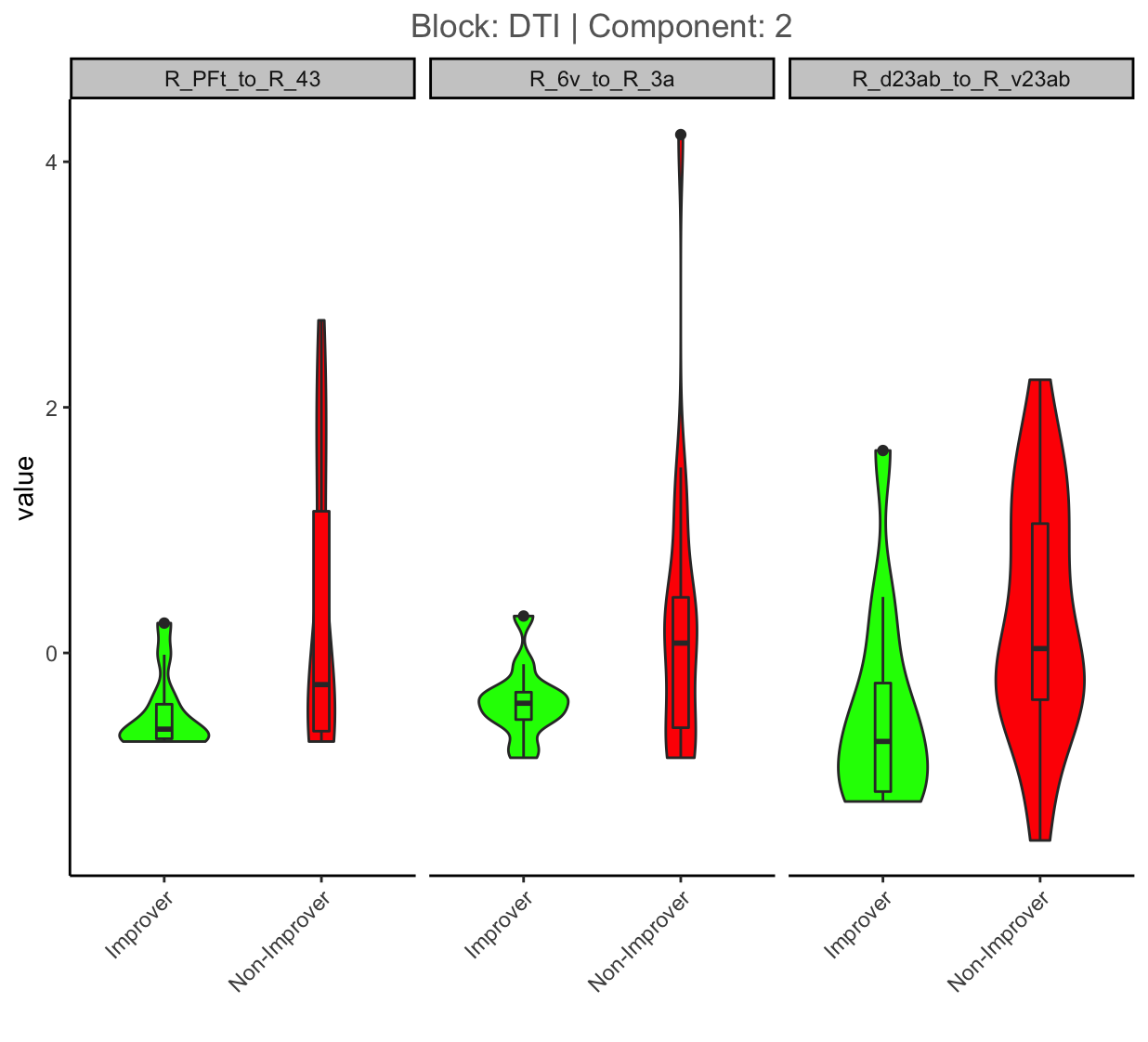

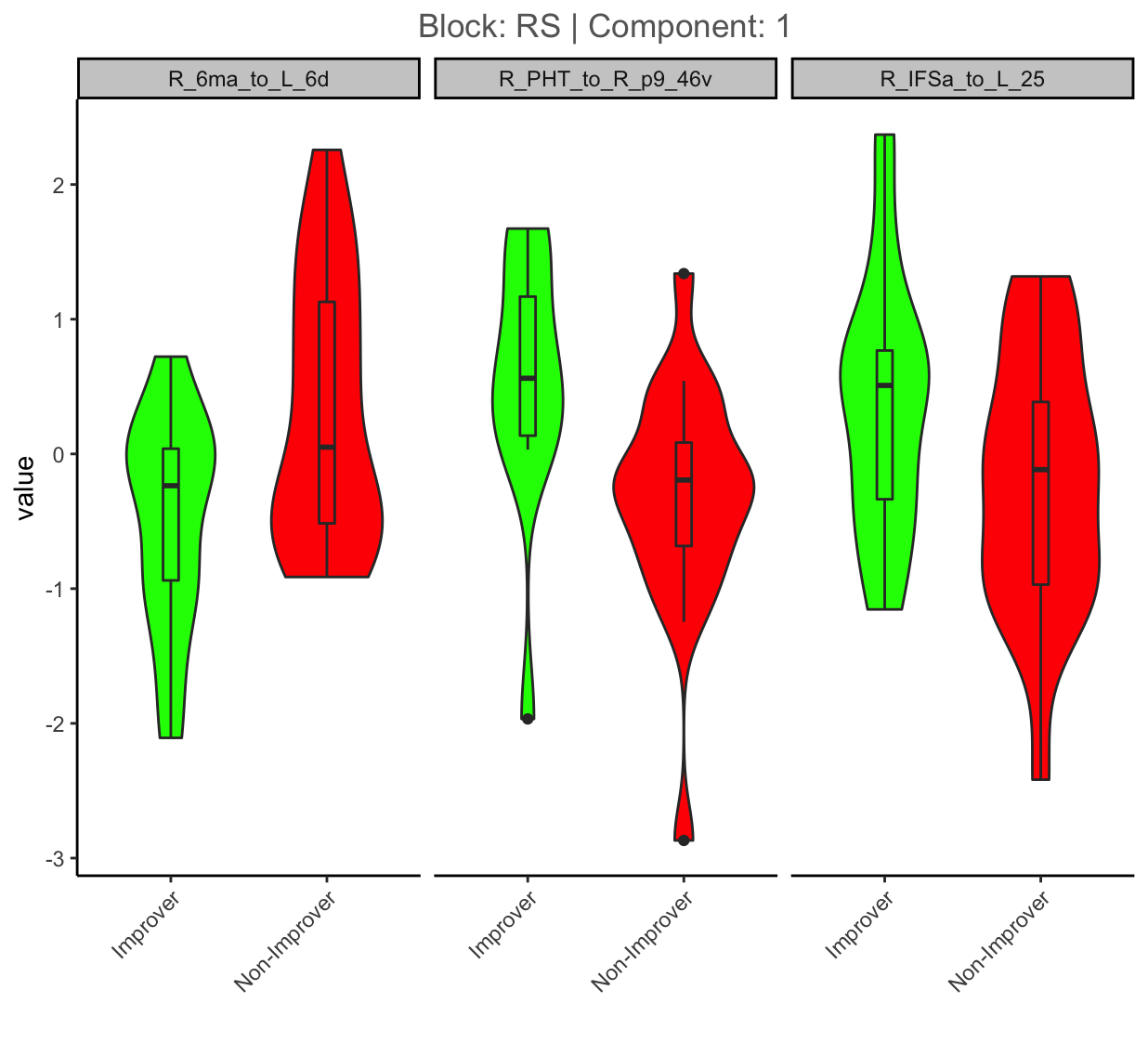

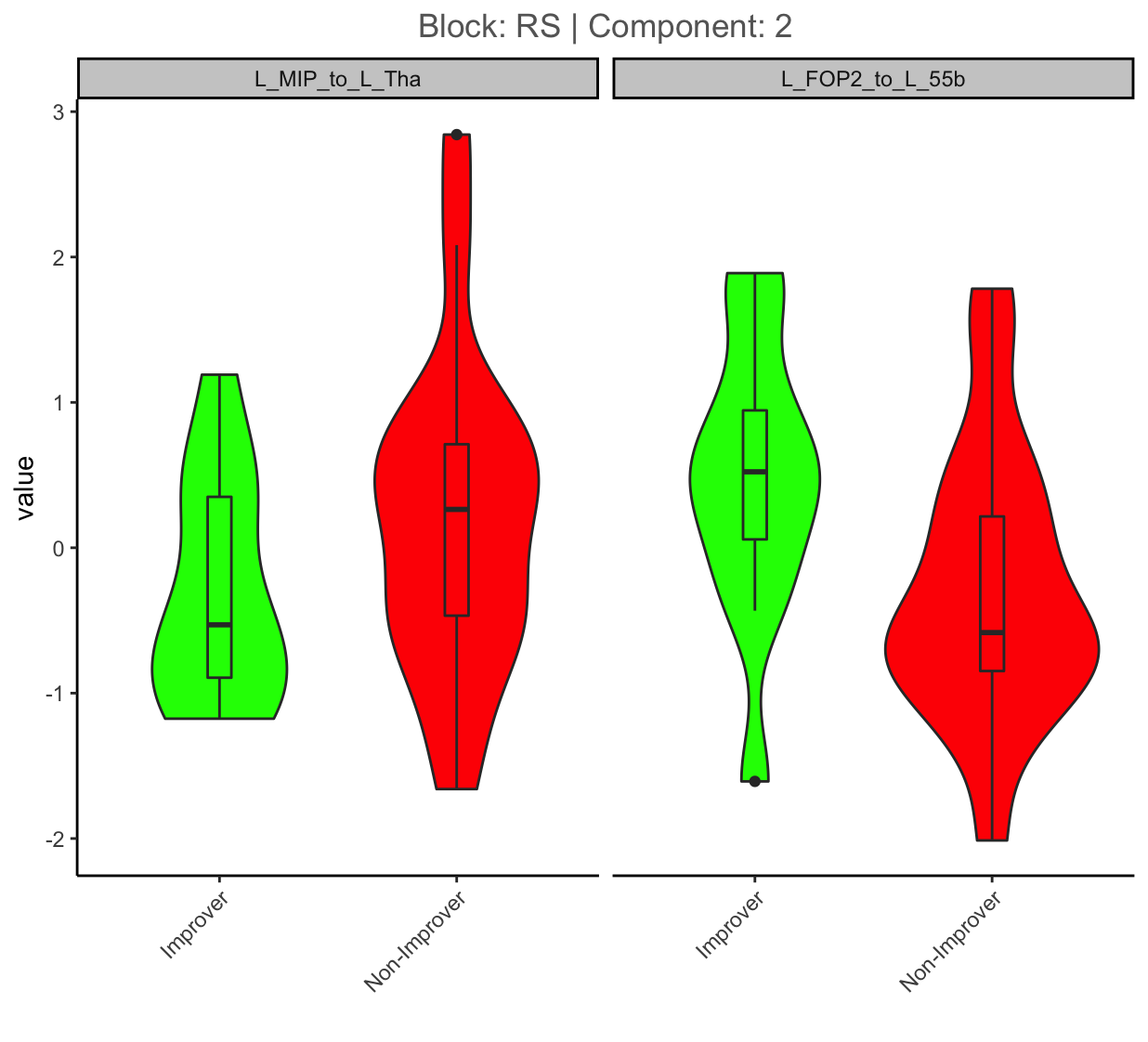

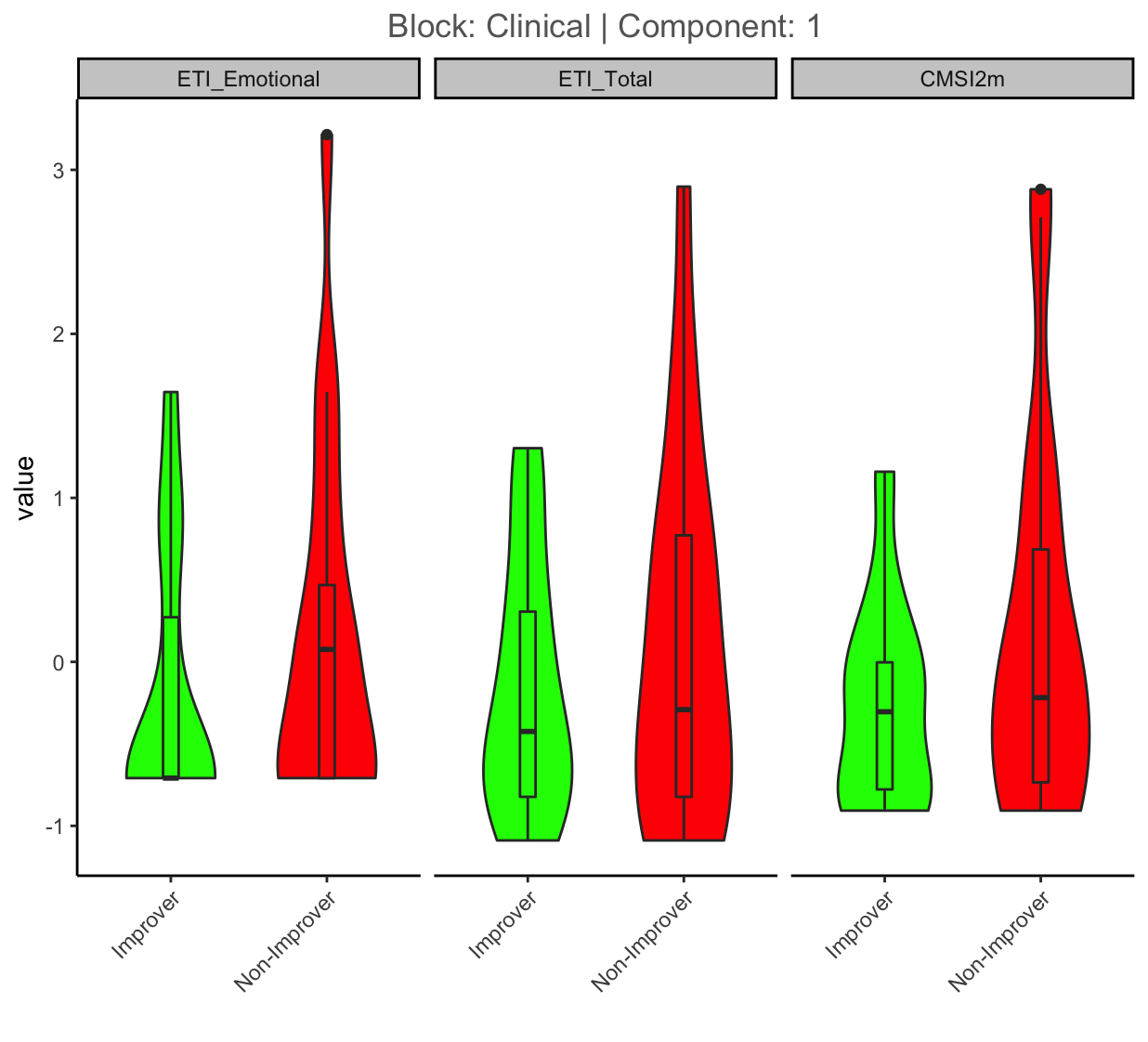

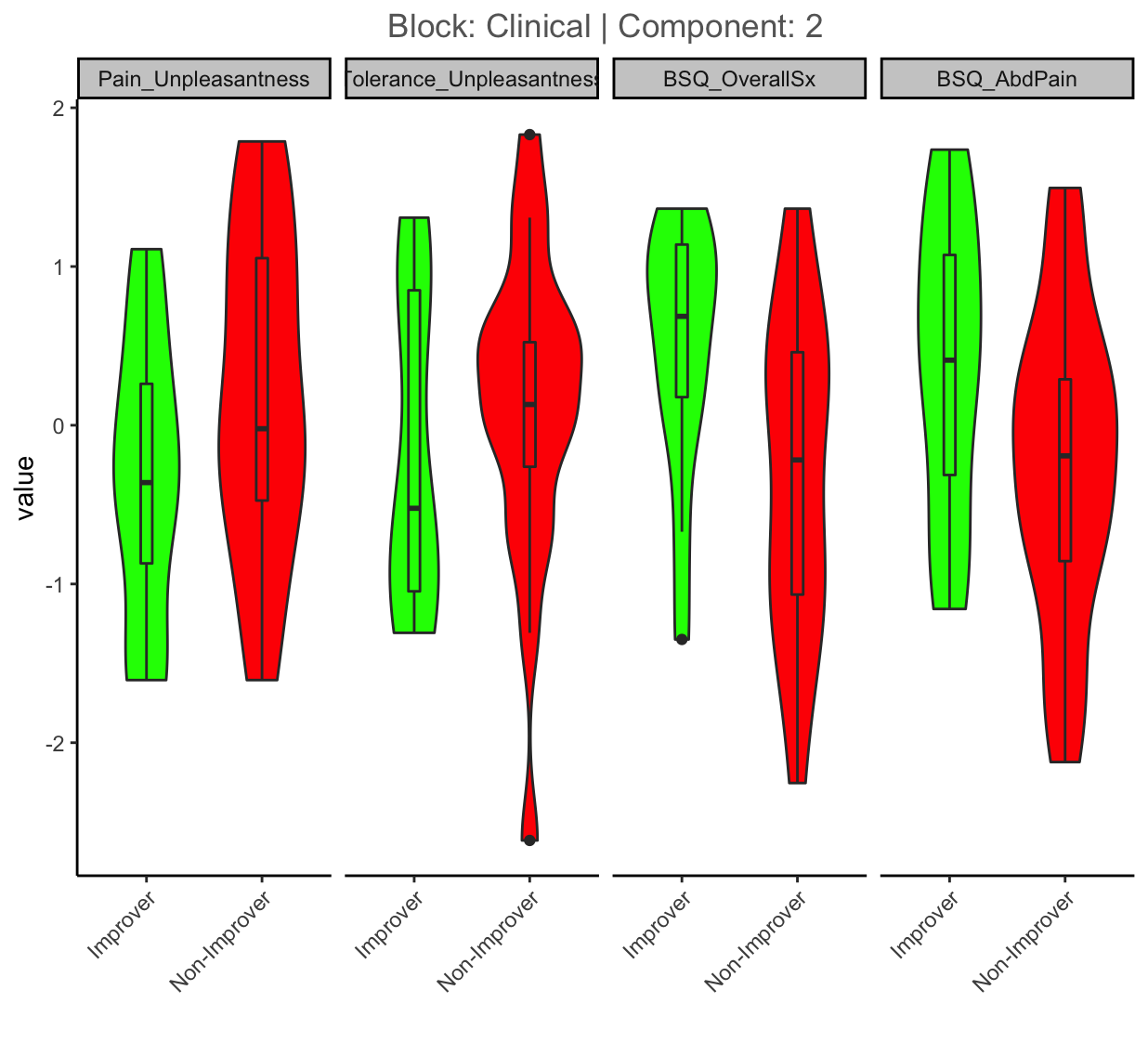


**References**

1. Francis CY, Morris J, Whorwell PJ. The irritable bowel severity scoring system: A simple method of monitoring irritable bowel syndrome and its progress. *Alimentary Pharmacology and Therapeutics*. 1997;11(2):395-402. doi:10.1046/j.1365-2036.1997.142318000.x

2. Talley NJ, Newman P, Boyce PM, Paterson KJ, Owen BK. Initial validation of a bowel symptom questionnaire and measurement of chronic gastrointestinal symptoms in Australians. *Australian and New Zealand Journal of Medicine*. 1995;25(4):302-308. doi:10.1111/j.1445-5994.1995.tb01894.x

3. Zigmond AS, Snaith RP. The Hospital Anxiety and Depression Scale. *Acta Psychiatrica Scandinavica*. 1983;67(6):361-370. doi:10.1111/j.1600-0447.1983.tb09716.x

4. Spielberger CD. State-Trait Anxiety Inventory (STAI). *Mind Garden*. 1983;94061(650):261-3500.

5. Cohen S, Kamarck T, Mermelstein R. A Global Measure of Perceived Stress. *Journal of Health and Social Behavior*. 1983;24:385-396. doi:10.2307/2136404

6. Goldberg LR, Johnson JA, Eber HW, et al. The international personality item pool and the future of public-domain personality measures. *Journal of Research in Personality*. 2006;40:84-96. doi:10.1016/j.jrp.2005.08.007

7. Bremner JD, Bolus R, Mayer EA. Psychometric properties of the early trauma inventory-self report. *Journal of Nervous and Mental Disease*. 2007;195(3):211-218. doi:10.1097/01.nmd.0000243824.84651.6c

8. Felitti VJ, Anda RF, Nordenberg D, et al. Relationship of childhood abuse and household dysfunction to many of the leading causes of death in adults: The adverse childhood experiences (ACE) study. *American Journal of Preventive Medicine*. 1998;14(4):245-258. doi:10.1016/S0749-3797(98)00017-8

9. Connor KM, Davidson JRT. Development of a new Resilience scale: The Connor-Davidson Resilience scale (CD-RISC). *Depression and Anxiety*. 2003;18(2):76-82. doi:10.1002/da.10113

10. Lovell RM, Ford AC. Effect of gender on prevalence of irritable bowel syndrome in the community: Systematic review and meta-analysis. *American Journal of Gastroenterology*. 2012;107(7):991-1000. doi:10.1038/ajg.2012.131

11. Fischl B. FreeSurfer. *NeuroImage*. 2012;62(2):774-781. doi:10.1016/j.neuroimage.2012.01.021

12. Glasser MF, Coalson TS, Robinson EC, et al. A multi-modal parcellation of human cerebral cortex. *Nature*. 2016;536(7615):171-178. doi:10.1038/nature18933

13. Makris N, Goldstein JM, Kennedy D, et al. Decreased volume of left and total anterior insular lobule in schizophrenia. *Schizophrenia Research*. 2006;83(2-3):155-171. doi:10.1016/j.schres.2005.11.020

14. Frazier JA, Chiu S, Breeze JL, et al. Structural Brain Magnetic Resonance Imaging of Limbic and Thalamic Volumes in Pediatric Bipolar Disorder. *Am J Psychiatry*. 2005;162(7):1256-1265.

15. Desikan RS, Ségonne F, Fischl B, et al. An automated labeling system for subdividing the human cerebral cortex on MRI scans into gyral based regions of interest. *NeuroImage*. 2006;31(3):968-980. doi:10.1016/j.neuroimage.2006.01.021

16. Goldstein JM, Seidman LJ, Makris N, et al. Hypothalamic Abnormalities in Schizophrenia: Sex Effects and Genetic Vulnerability. *Biological Psychiatry*. 2007;61:935-945. doi:10.1016/j.biopsych.2006.06.027

17. Nieto-Castanon A. *Handbook of Functional Connectivity Magnetic Resonance Imaging Methods in CONN*. Hilbert Press; 2020.

18. Collignon A, Maes F, Delaere D, Vandermeulen D, Suetens P, Marchal G. Automated multi-modality image registration based on information theory. *Information processing in medical imaging*. 1995;3(6):263-274. doi:10.1007/11784012_16

19. Studholme, Hill, Hawkes. A normalized entropy measure of 3-D medical image alignment. *Proc Medical Imaging*. 1998;3338:132-143.

20. Hagler DJ, Saygin AP, Sereno MI. Smoothing and cluster thresholding for cortical surface-based group analysis of fMRI data. *NeuroImage*. 2006;33(4):1093-1103. doi:10.1016/j.neuroimage.2006.07.036

21. Behzadi Y, Restom K, Liau J, Liu TT. A component based noise correction method (CompCor) for BOLD and perfusion based fMRI. *NeuroImage*. 2007;37(1):90-101.

22. Friston KJ, Williams S, Howard R, Frackowiak RSJ, Turner R. Movement-related effects in fMRI time-series. *Magnetic Resonance in Medicine*. 1996;35(3):364-355. doi:10.1002/mrm.1910350312

23. Power JD, Mitra A, Laumann TO, Snyder AZ, Schlaggar BL, Petersen SE. Methods to detect, characterize, and remove motion artifact in resting state fMRI. *NeuroImage*. 2014;84:320-341. doi:10.1016/j.neuroimage.2013.08.048

24. Whitfield-Gabrieli S, Nieto-Castanon A. Conn: A Functional Connectivity Toolbox for Correlated and Anticorrelated Brain Networks. *Brain Connectivity*. 2012;2(3):125-141. doi:10.1089/brain.2012.0073

25. Hallquist MN, Hwang K, Luna B. The nuisance of nuisance regression: Spectral misspecification in a common approach to resting-state fMRI preprocessing reintroduces noise and obscures functional connectivity. *NeuroImage*. 2013;82:208-225. doi:10.1016/j.neuroimage.2013.05.116

26. Andersson JLR, Sotiropoulos SN. An integrated approach to correction for off-resonance effects and subject movement in diffusion MR imaging. *NeuroImage*. 2016;125:1063-1078. doi:10.1016/j.neuroimage.2015.10.019

27. Cook P a, Bai Y, Seunarine KK, Hall MG, Parker GJ, Alexander DC. Camino: Open-Source Diffusion-MRI Reconstruction and Processing. *14th Scientific Meeting of the International Society for Magnetic Resonance in Medicine*. Published online 2006:2759.

28. Sarwar T, Ramamohanarao K, Zalesky A. Mapping connectomes with diffusion MRI: deterministic or probabilistic tractography? *Magnetic Resonance in Medicine*. 2019;81(2):1368-1384. doi:10.1002/mrm.27471

29. Voevodskaya O, Simmons A, Nordenskjöld R, et al. The effects of intracranial volume adjustment approaches on multiple regional MRI volumes in healthy aging and Alzheimer’s disease. *Frontiers in Aging Neuroscience*. 2014;6(264). doi:10.3389/fnagi.2014.00264

30. Barnes J, Ridgway GR, Bartlett J, et al. Head size, age and gender adjustment in MRI studies: A necessary nuisance? *NeuroImage*. 2010;53(4):1244-1255. doi:10.1016/j.neuroimage.2010.06.025

31. Rohart F, Gautier B, Singh A, Lê Cao KA. mixOmics: An R package for ‘omics feature selection and multiple data integration. *PLoS Computational Biology*. 2017;13(11):1-19. doi:10.1371/journal.pcbi.1005752

32. Singh A, Shannon CP, Gautier B, et al. DIABLO: An integrative approach for identifying key molecular drivers from multi-omics assays. *Bioinformatics*. 2019;35(17):3055-3062. doi:10.1093/bioinformatics/bty1054

33. González I, Cao KAL, Davis MJ, Déjean S. Visualising associations between paired “omics” data sets. *BioData Mining*. 2012;5(19):1-23. doi:10.1186/1756-0381-5-19

34. Hosmer DW, Lemeshow S, Sturdivant RX. *Applied Logistic Regression*. Third Edit. John Wiley & Sons; 2013. doi:10.1002/9781118548387

35. Lê Cao KA, Boitard S, Besse P. Sparse PLS discriminant analysis: Biologically relevant feature selection and graphical displays for multiclass problems. *BMC Bioinformatics*. 2011;12(253). doi:10.1186/1471-2105-12-253

36. Lê Cao KA, Martin PGP, Robert-Granié C, Besse P. Sparse canonical methods for biological data integration: Application to a cross-platform study. *BMC Bioinformatics*. 2009;10:1-17. doi:10.1186/1471-2105-10-34
